# Remote Participation during Glycosylation Reactions of Galactose Building Blocks: Direct Evidence from Cryogenic Vibrational Spectroscopy

**DOI:** 10.1002/anie.201916245

**Published:** 2020-03-02

**Authors:** Mateusz Marianski, Eike Mucha, Kim Greis, Sooyeon Moon, Alonso Pardo, Carla Kirschbaum, Daniel A. Thomas, Gerard Meijer, Gert von Helden, Kerry Gilmore, Peter H. Seeberger, Kevin Pagel

**Affiliations:** ^1^ Department of Chemistry and Biochemistry Hunter College 695 Park Ave 10065 New York NY USA; ^2^ The Ph.D. Program in Chemistry The Graduate Center of the City University of New York 365 5th Ave New York NY 10016 USA; ^3^ Fritz-Haber-Institut der Max-Planck-Gesellschaft Faradayweg 4–6 14195 Berlin Germany; ^4^ Max-Planck-Institut für Kolloid und Grenzflächenforschung Am Mühlenberg 1 14476 Potsdam Germany; ^5^ Institut für Chemie und Biochemie Freie Universität Berlin Takustrasse 3 14195 Berlin Germany

**Keywords:** glycosyl cations, glycosylation, mass spectrometry, reaction Intermediates, spectroscopy

## Abstract

The stereoselective formation of 1,2‐*cis*‐glycosidic bonds is challenging. However, 1,2‐*cis*‐selectivity can be induced by remote participation of C4 or C6 ester groups. Reactions involving remote participation are believed to proceed via a key ionic intermediate, the glycosyl cation. Although mechanistic pathways were postulated many years ago, the structure of the reaction intermediates remained elusive owing to their short‐lived nature. Herein, we unravel the structure of glycosyl cations involved in remote participation reactions via cryogenic vibrational spectroscopy and first principles theory. Acetyl groups at C4 ensure α‐selective galactosylations by forming a covalent bond to the anomeric carbon in dioxolenium‐type ions. Unexpectedly, also benzyl ether protecting groups can engage in remote participation and promote the stereoselective formation of 1,2‐*cis*‐glycosidic bonds.

## Graphical abstract

**Remote control**: The structure of glycosyl cations involved in remote participation is unraveled by using cryogenic ion IR spectroscopy and density‐functional theory. The acetyl groups at C4 promote the formation of α‐products by forming a covalent bond to the anomeric carbon atom in dioxolenium‐type ions.

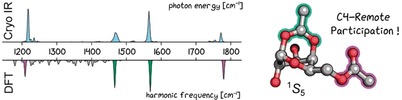

Glycans are biopolymers that consist of many different building blocks and exhibit complex regio‐ and stereochemistry such that their chemical synthesis has traditionally been very time consuming. Although regiocontrol is readily exerted using orthogonal protecting group strategies,^1^ stereocontrol during glycosidic bond formation can be challenging. In many cases, custom‐tailored approaches require empirical optimizations. For instance, 1,2‐*trans* glycosidic bonds are most reliably obtained using C2‐participating 2‐*O*‐ester protecting groups (PGs).^2^ In these systems, the cleavage of a leaving group at the anomeric carbon promotes the formation of a cyclic 1,2‐*cis* dioxolenium intermediate that facilitates a nucleophilic attack from the *trans*‐side.^3^ Stereocontrol during the formation of 1,2‐*cis* glycosides, however, requires shielding of the *trans*‐side. Various strategies are used to aid a nucleophilic attack from the *cis*‐side, such as chiral auxiliaries,^4^ intramolecular rearrangements,^5^ or specific activators.^6^ A more widely used alternative involves the remote participation of acyl protecting groups at the C4 or C6 position.^7^ Similar to C2‐participation, the reaction mechanism is believed to proceed via a key ionic intermediate, the glycosyl cation, where the *trans*‐side of the anomeric carbon is shielded by remote ester groups (Figure 1).^8^ Remote participation can, via a range of different possible intermediates, induce a nucleophilic attack and lead to *cis*‐glycosidic bonds, but the reactive and short‐lived nature of these intermediates has greatly impeded their direct experimental characterization.

**Figure 1:**
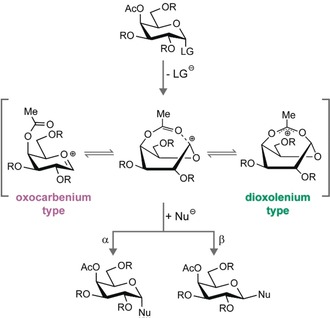
Stereoselective formation of 1,2‐*cis*‐galactosides involving remote participation of acetyl groups. While the reaction mechanism is believed to proceed via a glycosyl cation, the exact structure of the intermediate remains unclear. The intermediate ion structure could range from oxocarbenium‐type to hybrid and dioxolenium‐type featuring a covalent bond to the anomeric carbon. The exact intermediate structure impacts the stereochemistry of the newly formed glycosidic bond. LG: leaving group; Nu: nucleophile.

A series of reactions (Figure 2) served as the basis to systematically investigate the effect of remote participation on 1,2‐*cis* glycosylations. The nucleophilic substitution of isopropanol on the four galactose building blocks 2,3,4,6‐tetra‐*O*‐benzyl‐d‐galactopyranoside (**Bn**), 4‐*O*‐acetyl‐2,3,6‐tri‐*O*‐benzyl‐d‐galactopyranoside (**4Ac**), 6‐*O*‐acetyl‐2,3,4‐tri‐*O*‐benzyl‐d‐galactopyranoside (**6Ac**), and 4,6‐di‐*O*‐acetyl‐2,3‐di‐*O*‐benzyl‐d‐galactopyranoside (**4,6Ac**) was carried out at five different temperatures between −50 °C and 30 °C under well‐defined reaction conditions using flow chemistry (see Supporting Information for details). These glycosylating agents fall into two groups that either yield mainly α‐glycosides (**4Ac** and **4,6Ac**), or little α‐glycosides (**Bn** and **6Ac**). The difference in stereoselectivity between the two groups is consistent for all measured temperatures and suggests that different reaction intermediates are responsible for the reaction outcome. If the reactions would solely proceed via an S_N_2‐mechanism, the β‐glycoside would be formed exclusively, because α‐acetimidate donors are used. However, the relative abundance of α‐products between 20 % and 90 % suggests dissociative S_N_1‐mechanisms involving glycosyl cations. All building blocks show a consistent increase of α‐selectivity with increasing temperature, which is in agreement with the general assumption that a dissociative S_N_1‐mechanism is favored at higher temperatures due to a gain in entropy.^9^

**Figure 2:**
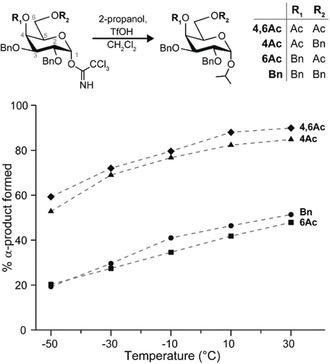
Reactions of galactose building blocks to assess the impact of different protecting group combinations (Bn=benzyl, Ac=acetyl) on the stereochemical outcome of the glycosylation reaction. The reactions were carried out at various temperatures using 2‐propanol as a nucleophile and the reaction outcome was determined by normal phase HPLC. With increasing temperature, the relative yield of α‐glycosides increases, indicating that the mechanistic continuum is effectively shifted towards S_N_1‐type reactions. Building blocks that carry an acetyl group at C4 (**4**,**6Ac** and **4Ac**) consistently show a higher α‐selectivity than the building blocks carrying an acetyl group at C6 (**6Ac**) or carrying no acetyl group (**Bn**).

The reaction pathway leading to the formation of α‐products is believed to proceed through a short‐lived and reactive glycosyl cation intermediate. The underlying reaction mechanism is difficult to study using classical condensed‐phase techniques such as NMR spectroscopy. In recent studies, various glycosyl cations were stabilized in superacids and studied via NMR spectroscopy. However, in the superacid medium, the protecting groups are protonated, such that results cannot be directly translated to classical reaction conditions.^10^ In another approach, the isolation of side products provided strong, but indirect evidence for remote participation by acetyl esters at the C4‐position of glycosyl donors.8c, 8d Alternatively, mass spectrometry‐based techniques can be used to isolate and characterize the glycosylation reaction intermediates in the gas phase.3a, 3b, 11, 12 Previously, we applied a combination of cryogenic ion spectroscopy and first‐principles theory to determine the exact structures of glycosyl cations of custom‐tailored model glycans carrying C2‐participating protecting groups.3a Boltje et al., on the other hand, applied infrared multiphoton dissociation (IRMPD) to investigate structures of a series of mannosyl donors.3b Recently, the same technique was applied to investigate whether remote participation promotes high β‐selectivity of bicyclic 6,3‐uronic acid lactones.^12^ Herein, we use more common, unconstrained glycosyl donors to unravel the structure of key intermediates involved in remote participation.^7^ It is important to note that acetyl protecting groups at the C3 position are also hypothesized to participate during glycosylation reactions via dioxolenium intermediates. For brevity, however, the investigation of C3‐acetylated donors was omitted and will be reported elsewhere.

The experimental setup to investigate the structure of glycosyl cations using vibrational spectroscopy in helium nanodroplets was described in detail before.^13^ Briefly, glycosyl cations were generated by nano‐electrospray ionization (nESI) and in‐source fragmentation of imidate or thioglycoside precursors (see Supporting Information). The *m*/*z*‐selected ions were accumulated inside a cryogenic (78 K) hexapole ion trap, which thermalizes the trapped ions by buffer gas cooling. Next, the ions were picked up by superfluid helium nanodroplets with an average size of 10^5^ helium atoms traversing the ion trap. The helium environment inside the droplets “shock‐freezes” the trapped ions to the droplet's equilibrium temperature of 0.4 K before they are irradiated with infrared radiation (between 1000–1800 cm^−1^), produced by the Fritz Haber Institute free‐electron Laser (FHI‐FEL^14^). The resonant absorption of multiple photons leads to the release of the bare intact ions from the droplet, this release is used as a messenger for photon absorption. Plotting the ion count of released ions as a function of IR wavelength yields a highly resolved IR signature of the investigated molecular ion.

To identify the structures responsible for the IR fingerprint, the molecular ions’ conformational space was explored using an evolutionary algorithm.15a The dispersion‐corrected PBE+vdW^TS [16]^ density functional, implemented in FHI‐aims,^17^ was chosen as a potential‐energy function because of its excellent accuracy for carbohydrates.15b Atomic connectivity is not constrained during the structural search which allows for proton transfers or formation of new bonds. This exhaustive conformational search included all rotatable bonds and ring puckers and yielded around 300 unique conformations for each glycosyl cation. These ensembles included various structural isomers, particularly those that feature a covalent bond between the anomeric carbon and distinct protecting groups. The structures were sorted according to the distance between the carbonyl oxygen of the acetyl group and the anomeric carbon into dioxolenium ions (distance less than 2 Å) and other structures (distance greater than 2 Å; see Supporting Information). For each type, a subset of all conformers within an energy window of 5 kcal mol^−1^ above the lowest energy structure was further optimized and frequencies calculated at the dispersion‐corrected hybrid density‐functional theory (DFT) PBE0‐D3/6‐311+G(d,p) level.^18^ For each reoptimized structure, we computed RI‐MP2‐level single‐point energies, extrapolated to the complete‐basis set.^19^ The harmonic IR spectra and free‐energy corrections at 78 K were derived from the frequency analysis. Ring puckers were assigned using Cremer–Pople coordinates.^20^

The IR spectra of the glycosyl cations show several absorption bands between 1000 cm^−1^ and 1800 cm^−1^, which can be grouped into two main regions. Below 1200 cm^−1^, complex C−O and C−C stretching modes dominate the spectrum. These mostly overlapping bands, however, are not sufficient for an unambiguous structural assignment. The region between 1200 cm^−1^ and 1800 cm^−1^, on the other hand, features characteristic modes, such as C=O stretch, C‐O‐C stretch, and O‐C‐O stretch vibrations. The exact position of these bands is strongly dependent on the type of interaction and therefore enables the structural identification. Because all hydroxy groups are protected, the spectral region around 3000 cm^−1^ does not carry any analytical bands.

The **4,6Ac** building block results in the highest selectivity for α‐glycosidic bond formation. The infrared spectrum of the corresponding glycosyl cation (Figure 3 a) displays six well‐resolved absorption bands. The low‐energy structure **A** of this species is predicted to adopt a ^1^ *S*_5_ ring pucker featuring a covalent bond between the carbonyl oxygen of the C4‐acetyl group and the anomeric carbon (1.52 Å). The characteristic bands associated with this bridging dioxolenium motif are the symmetric and antisymmetric O‐C‐O stretch modes predicted at 1463 cm^−1^ and 1566 cm^−1^, both in very good agreement with the experiment. Two additional characteristic bands for the non‐interacting C6‐acetyl group are predicted at 1217 cm^−1^ and 1772 cm^−1^, in line with the experimental absorption bands. The alternative structure **B**, where the dioxolenium motif originates from a covalent bond between the C6‐acetyl and the anomeric carbon, has a free‐energy of around 5 kcal mol^−1^ larger with respect to structure **A**. Predicted symmetric and antisymmetric O‐C‐O stretch modes in structure **B** at 1459 cm^−1^ and 1592 cm^−1^ agree less well with the experimental spectrum. Furthermore, a free‐energy difference of more than 14 kcal mol^−1^, and a poor spectral match eliminates oxocarbenium‐type structure **C** with two non‐participating acetyl groups from consideration. In summary, the IR signature of the **4,6Ac** galactosyl donor provides direct evidence that the C4‐acetyl group participates through a dioxolenium‐type intermediate that ensures α‐selectivity, whereas participation via the C6‐acetyl group is not observed.

**Figure 3:**
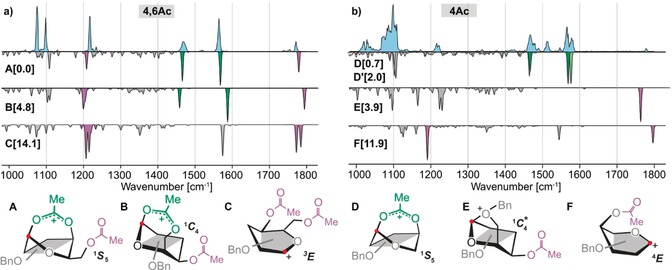
Infrared spectra of the glycosyl cations generated from a) the **4,6Ac** and b) the **4Ac** precursor. Blue traces are experimental IR spectra while gray inverted spectra are calculated corresponding to the low‐energy conformers shown below in a simplified representation. Numbers in square brackets indicate free energies in kcal mol^−1^. Full structures are shown in the Supporting Information. The highlighted absorption bands indicate vibrations from free acetyl groups (purple) and participating acetyl groups in dioxolenium‐type structures (green). The light green band indicates a second dioxolenium‐type conformer **D′**. For both building blocks, the majority of ions adopt dioxolenium structures that exhibit a covalent bond between the C4‐acetyl group and the anomeric carbon (highlighted with a red dot). Structure **E** shows a distorted ^1^ *C*_4_ ring pucker, indicated by a star (*).

The IR spectrum of **4Ac** (Figure 3 b) displays characteristic bands between 1450 cm^−1^ and 1600 cm^−1^, similar to those observed for the **4,6Ac** cation. The calculated vibrations at 1465 cm^−1^ and 1568 cm^−1^ for dioxolenium structure **D** featuring a ^1^ *S*_5_ ring pucker agree well with the experiment. The lowest free‐energy structure is more stable by 0.7 kcal mol^−1^, but shows a slightly worse agreement to the experimental data (see Supporting Information). The presence of multiple bands around 1500 cm^−1^ indicates that a structurally very similar dioxolenium ion coexists (Δ*F*=2.0 kcal mol^−1^, **D′**). The IR spectrum furthermore contains two additional low intensity bands at 1220 cm^−1^ and 1780 cm^−1^, indicating that a fraction of glycosyl cations adopt other structures. These bands can be assigned to the oxonium structure **E** that exhibits an interaction between C6‐OBn and the anomeric carbon. This structure has a free‐energy of around 4 kcal mol^−1^ larger, relative to the lowest energy structure, and the vibrations of the free acetyl group at 1227 cm^−1^ and 1763 cm^−1^ align well with the experimentally resolved bands. Observing this mode of remote participation is particularly surprising, because synthetic chemists consider benzyl protecting groups as non‐participating. The most stable oxocarbenium structure **F** has a much higher free energy (Δ*F*=11.9 kcal mol^−1^) and the predicted vibrations agree less well with the experiment. The spectroscopic data provides evidence that the **4Ac** cation predominantly adopts an α‐selective dioxolenium structure, similar to the one observed for **4,6Ac**.

The glycosyl donors **6Ac** and **Bn** consistently produce a low to medium abundance of α‐products, suggesting that the underlying intermediates are structurally different from those observed for **4,6Ac** and **4Ac**. The IR spectrum of the glycosyl cation corresponding to **6Ac** (Figure 4 a) displays a variety of absorption bands above 1200 cm^−1^. The lowest free‐energy structure **G** shows a dioxolenium motif with a ^1^ *C*_4_ ring pucker and is predicted to have two characteristic modes at 1457 cm^−1^ and 1593 cm^−1^. Although matching vibrations can be found in the experimental spectrum, the higher energy band shows very low intensity. Another low free‐energy structure is oxonium structure **H**, which is characterized by a covalent bond between the C4‐oxygen and the anomeric carbon in a ^1,4^ *B* ring‐pucker. This mode of remote participation was recently reported by Boltje et al. after observing a 1,4‐anhydro‐3,6‐lactone as a side product during the stereoselective synthesis of 1,2‐*cis* mannosides. This side product strongly suggests the presence of a glycosyl oxonium ion with remote participation of the oxygen atom of the C4 benzyl group.^12^ The predicted vibrations of the free acetyl group at 1220 cm^−1^ and 1762 cm^−1^ match the two prominent bands in the experimental spectrum. A different, energetically very similar oxocarbenium structure **I** features two bands of the free acetyl group at 1222 cm^−1^ and 1749 cm^−1^, with an additional distinct vibration associated to the oxocarbenium [C_1_=O_5_^+^] stretch at 1567 cm^−1^ that can be found in the experimental spectrum. In this structure, the oxocarbenium motif is stabilized by a long‐range interaction (2.8 Å) with the C6‐oxygen. Although both structures **H** and **I** are energetically less favored by around 4 kcal mol^−1^, the match between theory and experiment shows that either one or both of these intermediates are present. None of these structures, however, explain the three bands at 1336 cm^−1^, 1412 cm^−1^, and 1464 cm^−1^. An expanded conformational search, that included structural rearrangements, such as a proton shift towards an acetyl or benzyl‐group did not identify the origin of these bands (see Supporting Information). Either yet another (unknown) structure is present, or the harmonic approximation cannot resolve the position and intensity of anharmonic absorption bands involving a shared proton present in some structures. Nevertheless, the lack of diagnostic bands for the dioxolenium‐motif and the low to medium abundance of α‐products in the test reactions suggest that the α‐selective dioxolenium‐type ion **G** is a minor reaction channel that is suppressed by oxonium structure **H** and oxocarbenium structure **I**.

**Figure 4:**
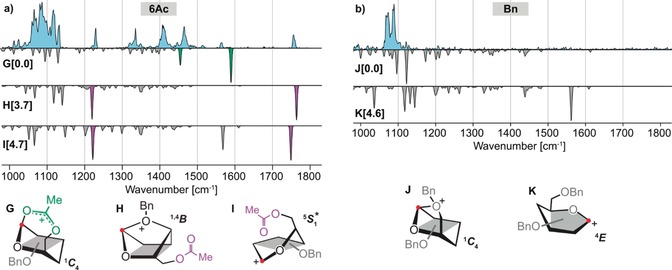
Infrared spectra of the glycosyl cations generated from a) the **6Ac** and b) the **Bn** precursors. The blue traces show the experimental IR spectra and the gray inverted spectra are calculated spectra corresponding to the low‐energy conformers shown below in a simplified representation. Numbers in square brackets indicate free energies in kcal mol^−1^. The complete structures are shown in the Supporting Information. The highlighted absorption bands indicate vibrations from free acetyl groups (purple) and participating acetyl groups in dioxolenium‐type structures (green). Interestingly, both structures show non‐classical remote participation of benzyl groups at C4 or C6, leading to α‐selective oxonium intermediates. Structure **I** shows a heavily distorted ring pucker, closest to a ^5^ *S*_1_ and ^5^ *H*_4_ conformation, indicated by a star (*).

The low abundance of α‐glycosides for **6Ac** is comparable to the fully benzylated building block **Bn**. The IR spectrum of the corresponding glycosyl cation (Figure 4 b) does not feature any significant absorption bands above 1200 cm^−1^. Low‐energy structure **K**, however, is expected to show a strong diagnostic band at 1550 cm^−1^ that is associated to the oxocarbenium motif. A better match for the experimental spectrum is found for oxonium structure **J** that is characterized by a covalent bond between the anomeric carbon and the C6‐oxygen in a ^1^ *C*_4_ ring pucker. Interestingly, the remote participation via the C6 benzyl group renders this intermediate energetically more stable than structure **K** by 4.6 kcal mol^−1^. Although glycosylation reactions through this intermediate should be highly α‐selective, the test reactions yield of α‐product is below 50 %. This finding indicates that the gain in energy owing to entropy promoting an S_N_1‐type mechanism through structure **J** is weakened by an enthalpic penalty, which effectively shifts the glycosylation reaction to the S_N_2‐end of the mechanistic continuum. Note, however, that the strong nucleophile used in the reactions generally leads to more S_N_2‐type reactions.^21^ In oligosaccharide synthesis, the nucleophiles (glycosyl acceptors) are usually weaker and highly α‐selective reactions for this building block (α:β‐ratio of 14:1) have been reported.^7^ This suggests that the α‐selectivity of this building block is based on remote participation of the C6‐benzyl group.

In summary, we provide direct evidence for remote participation in galactose building blocks routinely used in oligosaccharide syntheses. α‐Selectivity is achieved by a C4 acetyl group, promoting energetically preferred dioxolenium ions with a covalent bond between the carbonyl oxygen and the anomeric carbon. Such a dioxolenium structure is energetically less favored for acetyl groups at the C6‐position, leading to the formation of oxocarbenium or oxonium structures observed for building block **6Ac**. The presence of oxonium structures is particularly surprising, because they involve the remote participation of benzyl protecting groups that promote the formation of α‐glycosides. For the fully benzylated **Bn** building block, remote participation via the C6‐benzyl group is energetically preferred and leads to an α‐selective oxonium intermediate. Our observations provide a structural basis for the different modes of remote participation, which are essential during the stereoselective formation of 1,2‐*cis*‐glycosidic bonds. By removing the influence of solvent and counter ions, the intrinsic stereoselectivity of glycosyl cations can be studied as a basis to design glycosyl donors by tuning the electronic properties of participating groups.

## Conflict of interest

The authors declare no conflict of interest.

## Supporting information

As a service to our authors and readers, this journal provides supporting information supplied by the authors. Such materials are peer reviewed and may be re‐organized for online delivery, but are not copy‐edited or typeset. Technical support issues arising from supporting information (other than missing files) should be addressed to the authors.

Supplementary
