# Supplementary material for: Remote Participation during Glycosylation Reactions of Galactose Building Blocks: Direct Evidence from Cryogenic Vibrational Spectroscopy
Source: Angew Chem Int Ed Engl. 2020 Mar 2;59(15):6166–71. doi: 10.1002/anie.201916245 (PMC7187407; doi:10.1002/anie.201916245)
Supplement: Supplementary file 1 — Supplementary [file ANIE-59-6166-s001.pdf]

Angewandte Chemie
Eine Zeitschrift der Gesellschaft Deutscher Chemiker

Supporting Information

Remote Participation during Glycosylation Reactions of Galactose Building Blocks: Direct Evidence from Cryogenic Vibrational Spectroscopy

Mateusz Marianski, Eike Mucha, Kim Greis, Sooyeon Moon, Alonso Pardo, Carla Kirschbaum, Daniel A. Thomas, Gerard Meijer, Gert von Helden, Kerry Gilmore, Peter H. Seeberger, and Kevin Pagel**

anie_201916245_sm_miscellaneous_information.pdf

Calculated Low Energy Structures

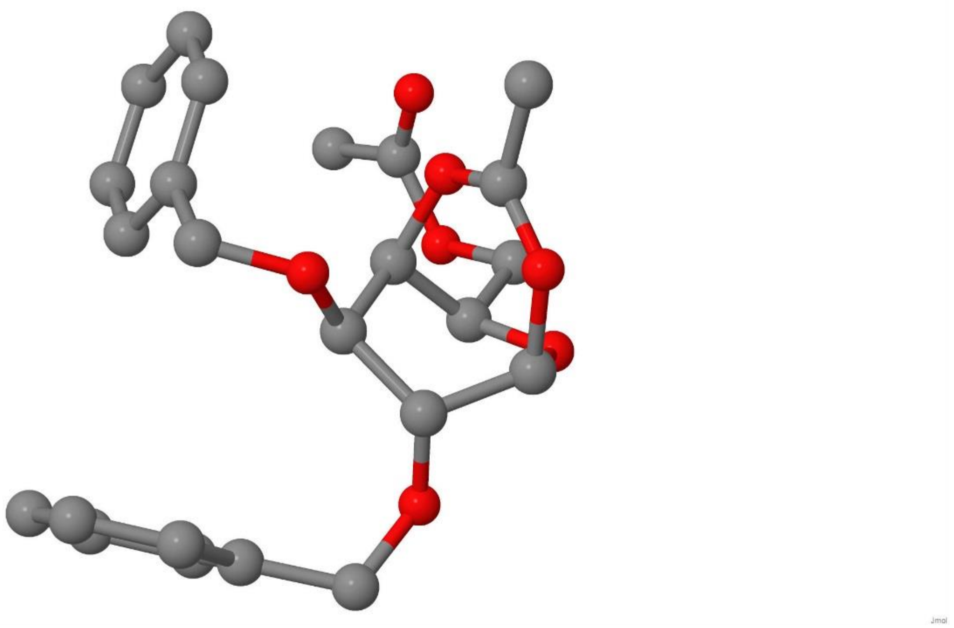

Figure S1: Calculated structure of glycosyl cation **A** generated from the **4,6Ac** building block. Hydrogens are omitted for clarity.

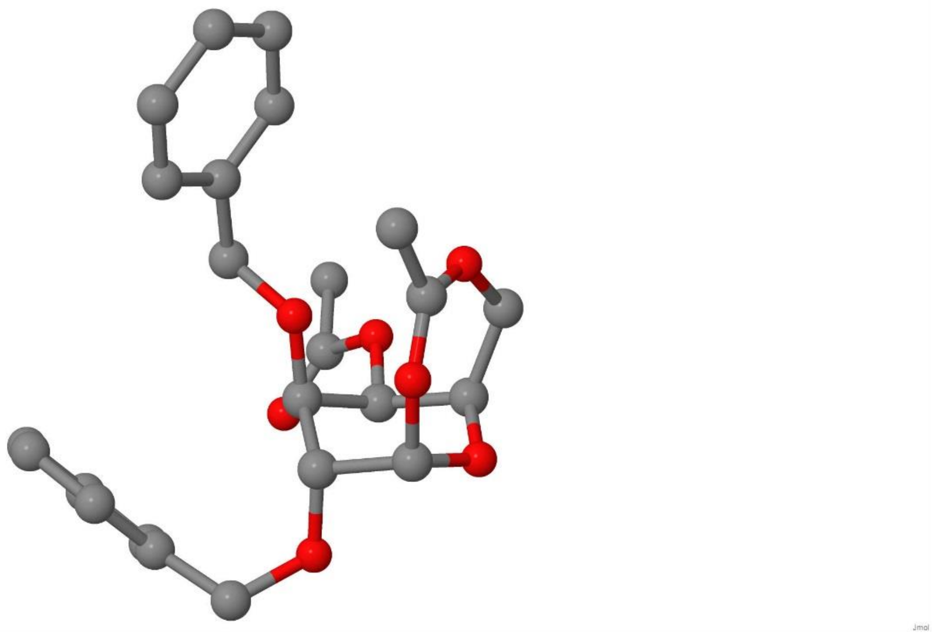

Figure S2: Calculated structure of glycosyl cation **B** generated from the **4,6Ac** building block. Hydrogens are omitted for clarity.

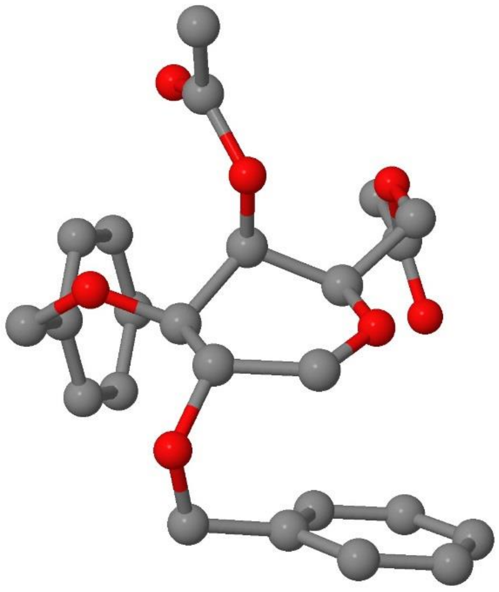

Figure S3: Calculated structure of glycosyl cation **C** generated from the **4,6Ac** building block. Hydrogens are omitted for clarity.

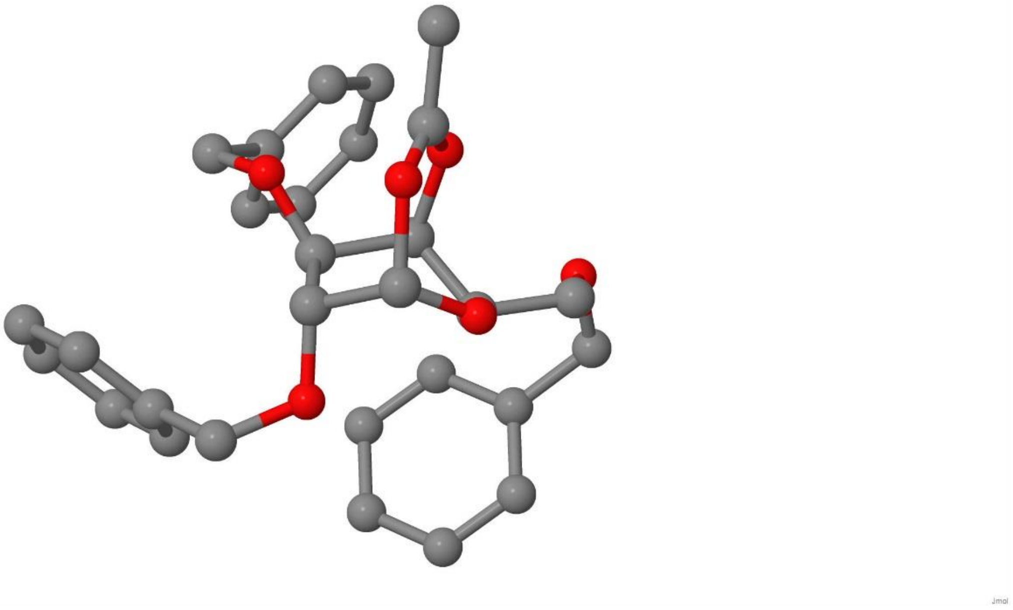

Figure S4: Calculated structure of glycosyl cation **D** generated from the **4Ac** building block. Hydrogens are omitted for clarity.

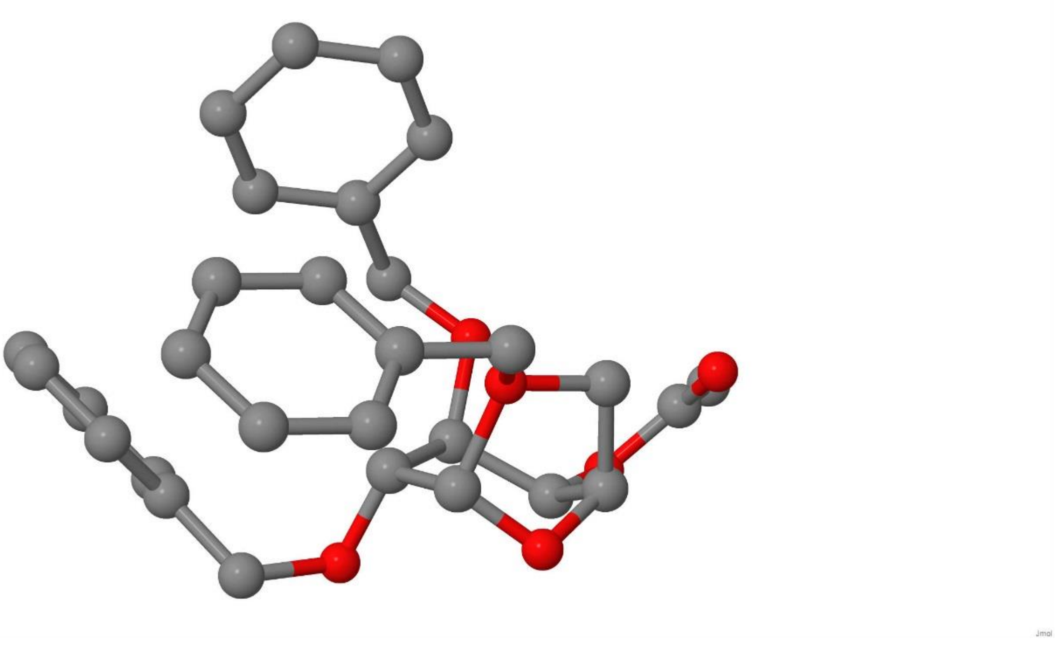

Figure S5: Calculated structure of glycosyl cation **E** generated from the **4Ac** building block. Hydrogens are omitted for clarity.

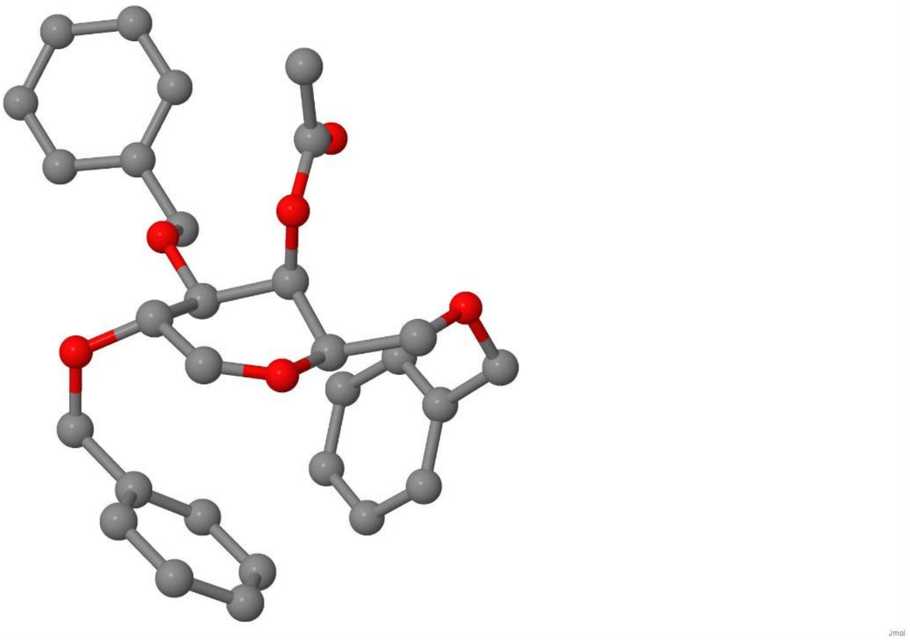

Figure S6: Calculated structure of glycosyl cation **F** generated from the **4Ac** building block. Hydrogens are omitted for clarity.

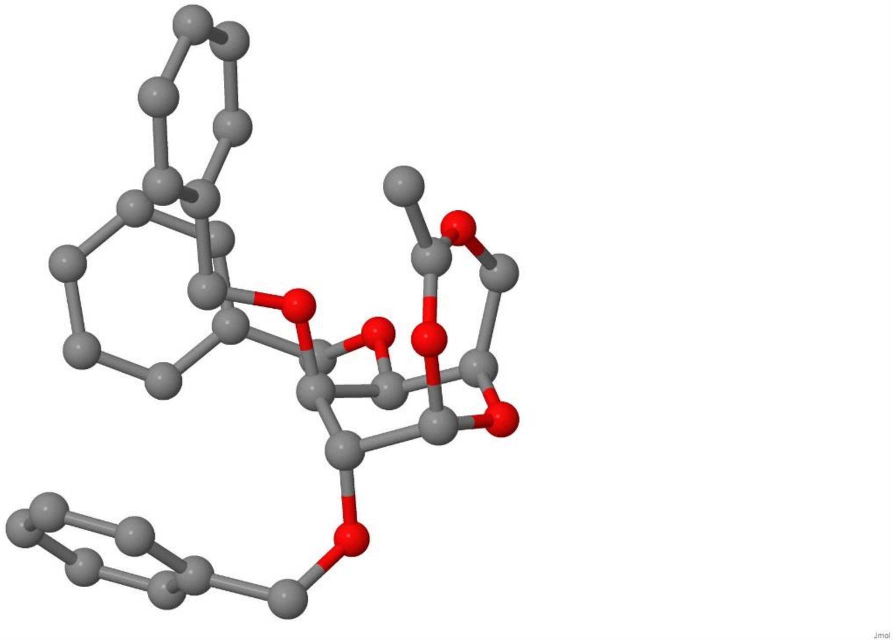

Figure S7: Calculated structure of glycosyl cation **G** generated from the **6Ac** building block. Hydrogens are omitted for clarity.

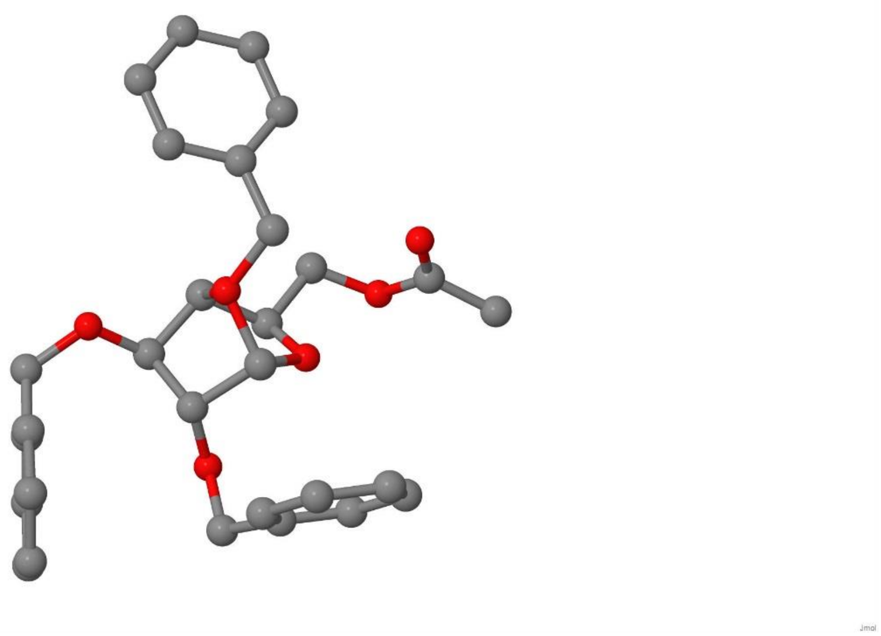

Figure S8: Calculated structure of glycosyl cation **H** generated from the **6Ac** building block. Hydrogens are omitted for clarity.

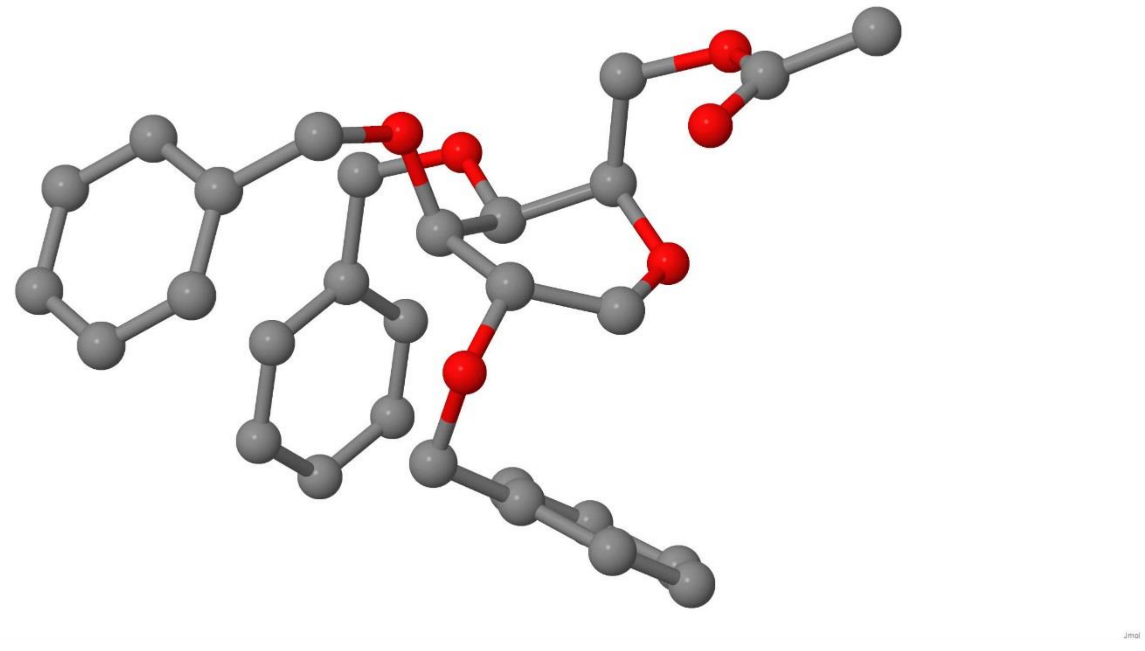

Figure S9: Calculated structure of glycosyl cation **I** generated from the **6Ac** building block. Hydrogens are omitted for clarity.

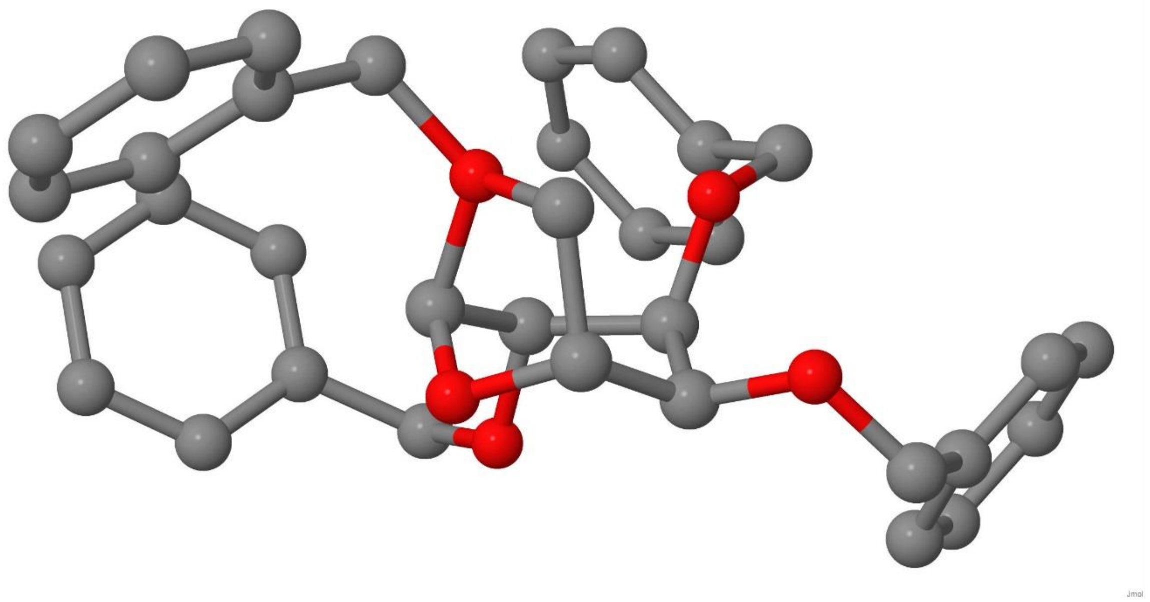

Figure S10: Calculated structure of glycosyl cation **J** generated from the **Bn** building block. Hydrogens are omitted for clarity.

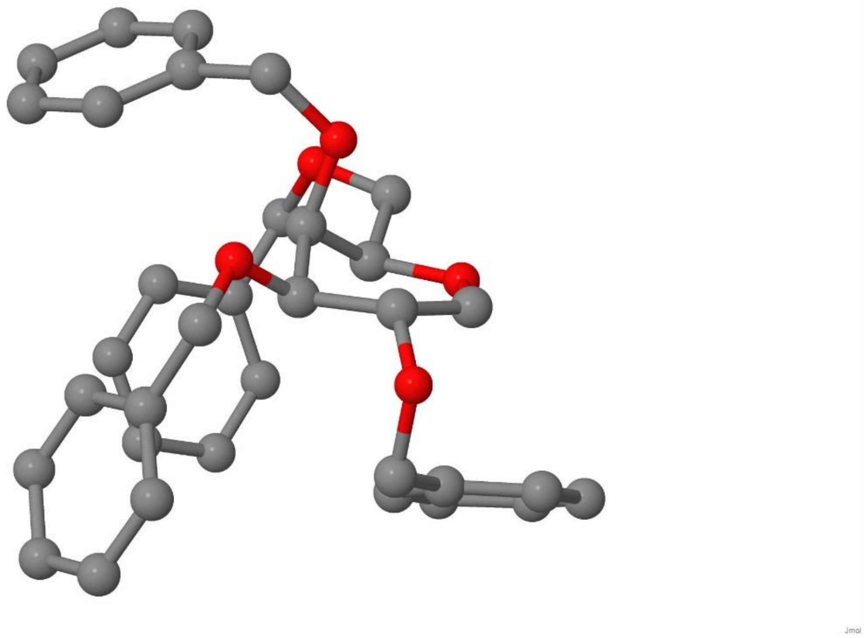

Figure S11: Calculated structure of glycosyl cation **K** generated from the **Bn** building block. Hydrogens are omitted for clarity.

Synthesis

General experimental details for preparing building blocks

Commercial grade solvents and reagents were used unless stated otherwise. Anhydrous solvents were obtained by **Procedure for drying of Solvents**. Unless otherwise noted, all other reagents and solvents were purchased from commercial suppliers and used without further purification. All reactions were carried out under an argon atmosphere. Analytical thin layer chromatography (TLC) was performed on Macherey-Nagel Pre-coated TLC-sheets, ALUGRAM Xtra SIL G/UV254 sheets and visualized with 254 nm light, 2,5-dinitrophenylhydrazine (DNPH) staining solutions followed by heating. Purification of the reaction products was carried out by flash chromatography using Macherey-Nagel Silica 60 M (0.04-0.063 mm) silica gel. Proton (^{1}H) NMR spectra were recorded using Agilent 400 (400 MHz) or Agilent 600 (600 MHz) in $CDCl_3$ and are reported in ppm relative to the residual solvent peaks ($CDCl_3$ at 7.26 ppm) Peaks are reported as: s = singlet, d = doublet, t = triplet, q = quartet, quint = quintet, m = multiplet. Carbon (^{13}C) NMR spectra were recorded with 1H-decoupling on Agilent 400 (101 MHz) or Agilent 600 (151 MHz) in $CDCl_3$ and reported in ppm relative to the residual solvent peak ($CDCl_3$ at 77.16 ppm). High-resolution mass spectral data were obtained using a Waters XEVO G2-XS 4K spectrometer (#186008532) with the XEVO G2-XS QTOF capability kit (#1860083535). Samples were prepared in LC-MS CHROMASOLV water and acetonitrile, and analyzed in the respective mixtures.

Procedure for drying of Solvents

Solvents used in this study such as dichloromethane (DCM) was dried using 3 Å molecular sieves, activated by heating under microware radiation of 500 W for 10 mins and subsequent cooling to ambient temperature under high vacuum. This procedure was repeated three times. The activated molecular sieves were added to the solvents and the solvents were kept under argon atmosphere for two days. The water content of the solvents was determined using Karl Fischer titration.

Preparation of Ethyl 4,6-di-O-acetyl-2,3-di-O-benzyl-1-thio-β-D-galactopyranoside (S2)[1]

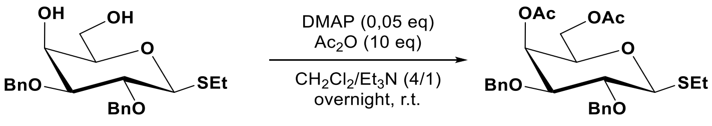

S1 **S2**

To a solution of compound **S1**[1] (93.8 mg, 0.23 mmol) in CH_2Cl_2/Et_3N (4/1) (2.5 mL) were added 4-dimethylaminopyridine (DMAP) (1.42 mg, 0.01 mmol) and Ac_2O (0.24 mL, 2.32 mmol) and the mixture was stirred at room temperature overnight. After the mixture was quenched with sat. aq.$NaHCO_3$ (15 mL), organic phase was extracted with DCM (3 × 30 mL) and the organic layers were washed with brine (2 × 50 mL). Combined organic phase was dried over $NaSO_4$, and evaporated in vacuo. The crude product was purified by column chromatography on silica gel (Elution: *n*-Hexane/ EtOAc = 10/1), **S2** (97.7 mg, 0.20 mmol) was obtained with 85% yield as a pale yellow oil (Rf: 0.56 in *n*-Hexane/ EtOAc = 3/1). ^{1}H NMR (400 MHz, Chloroform-*d*) δ 7.34 (dq, *J* = 16.9, 6.7, 6.2 Hz, 10H), 5.55 (s, 1H), 4.87 – 4.71 (m, 3H), 4.56 – 4.45 (m, 2H), 4.15 (d, *J* = 6.5 Hz, 2H), 3.79 (t, *J* = 6.5 Hz, 1H), 3.61 (q, *J* = 10.1, 8.9 Hz, 2H), 2.76 (qt, *J* = 13.3, 7.8 Hz, 2H), 2.16 (s, 3H), 2.07 (s, 3H), 1.33 (t, *J* = 7.4 Hz, 3H). This data is in accordance with those previously published.[1]

Preparation of 4,6-di-O-acetyl-2,3-di-O-benzyl-α-D-galactopyranosyl trichloroacetimidate (S4α)

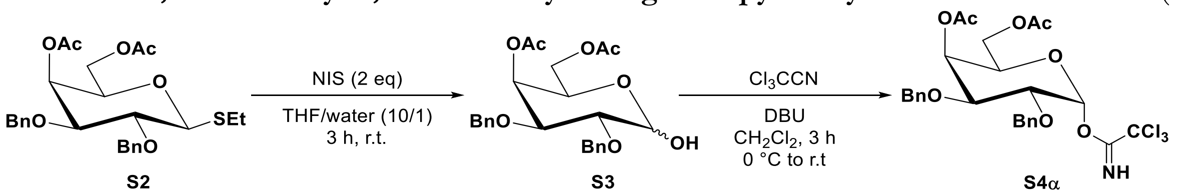

S2 **S3** **S4α**

To a solution **S2** (97.7 mg, 0.20 mmol) in THF (3 mL) and water (0.3 mL) were added N-iodosuccinimide (87.7 mg, 0.39 mmol) and 1M HCl 2 drops, and then stirred for 3 h at room temperature. The reaction mixture was quenched with with sat. aq. $NaHCO_3$ solution (10 mL) and DCM (10 mL). The organic layer was extracted with DCM (2 × 10 mL) and washed with brine (10 mL). The organic layer was dried over anhydrous Na_2SO_4, filtered and evaporated under reduced pressure for column chromatography purification (Elution: n-hexane/EtOAc = 4/1 to 2/1) and obtained as an inseparable α/β mixture **S3** (Rf: 0.15 in *n*-Hexane/ EtOAc = 3/1). Compound **S3** in dry DCM (10 mL) were added CCl_3CN (0.1 mL, 1 mmol) and DBU (0.03 mL, 0.02 mmol) at 0 °C. The dark solution was stirred at room temperature for 3 h, and then the reaction mixture was concentrated. The residue was purified by silica gel column chromatography (Elution: n-hexane/EtOAc = 10/1 containing 1% Et_3N) to give **S4α** (76.6 mg, 0.13 mmol) as pale yellow oil (Rf: 0.67 in *n*-Hexane/ EtOAc = 3/1). with 65% yield; ^{1}H NMR (600 MHz, Chloroform-*d*) δ 8.61 (s, 1H), 7.36 – 7.24 (m, 10H), 6.53 (d, *J* = 3.4 Hz, 1H), 5.63 (d, *J* = 3.2 Hz, 1H), 4.80 – 4.69

(m, 3H), 4.59 (d, *J* = 11.4 Hz, 1H), 4.33 (t, *J* = 6.5 Hz, 1H), 4.18 (dd, *J* = 11.4, 6.0 Hz, 1H), 4.08 – 4.01 (m, 2H), 3.98 (dd, *J* = 10.0, 3.5 Hz, 1H), 2.13 (s, 3H), 2.03 (s, 3H). This data is in accordance with those previously published.[2]

Preparation of 4,6-di-O-acetyl-2,3-di-O-benzyl-β-D-galactopyranosyl trichloroacetimidate (S4β)

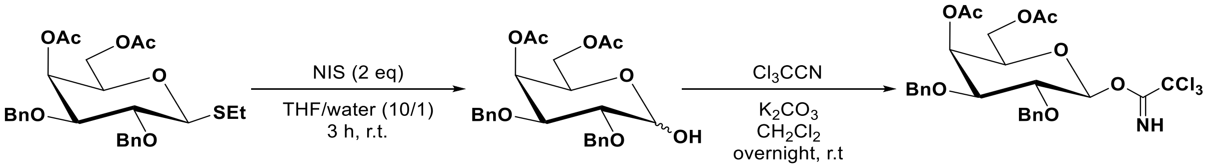

S2 S3 S4β

To a solution **S2** (97.7 mg, 0.20 mmol) in THF (3 mL) and water (0.3 mL) were added N-iodosuccinimide (87.7 mg, 0.39 mmol) and 1M HCl 2 drops, and then stirred for 3 h at room temperature. The reaction mixture was quenched with with sat. aq. $NaHCO_3$ solution (10 mL) and DCM (10 mL). The organic layer was extracted with DCM (2 × 10 mL) and washed with brine (10 mL). The organic layer was dried over anhydrous Na_2SO_4, filtered and evaporated under reduced pressure for column chromatography purification (Elution: n-hexane/EtOAc = 4/1 to 2/1) and obtained as an inseparable α/β mixture **S3** (Rf: 0.15 in *n*-Hexane/ EtOAc = 3/1). Compound **S3** in dry DCM (10 mL) were added CCl_3CN (0.1 mL, 1 mmol) and K_2CO_3 (138 mg, 1 mmol) at room temperature. The solution was stirred at room temperature overnight, and then the reaction mixture was concentrated. The residue was purified by silica gel column chromatography (Elution: n-hexane/EtOAc = 10/1 containing 1% Et_3N) to give **S4β** (67.1 mg, 0.11 mmol) as colorless oil (Rf: 0.60 in *n*-Hexane/ EtOAc = 3/1). with 57% yield; ^1H NMR (600 MHz, Chloroform-*d*) δ 8.69 (s, 1H), 7.34 – 7.27 (m, 10H), 5.79 (d, *J* = 8.1 Hz, 1H), 5.56 (dd, *J* = 3.5, 1.3 Hz, 1H), 4.87 (d, *J* = 10.7 Hz, 1H), 4.78 (dd, *J* = 10.9, 8.8 Hz, 2H), 4.54 (d, *J* = 11.2 Hz, 1H), 4.21 (dd, *J* = 11.3, 6.8 Hz, 1H), 4.16 (dd, *J* = 11.2, 6.5 Hz, 1H), 3.97 (td, *J* = 6.6, 1.3 Hz, 1H), 3.86 (dd, *J* = 9.6, 8.1 Hz, 1H), 3.71 (dd, *J* = 9.6, 3.5 Hz, 1H), 2.18 (s, 3H), 2.07 (s, 3H). This data is in accordance with those previously published.[2]

Preparation of 4-O-acetyl-2,3,6-tri-O-benzyl-α-D-galactopyranosyl trichloroacetimidate (S7α)

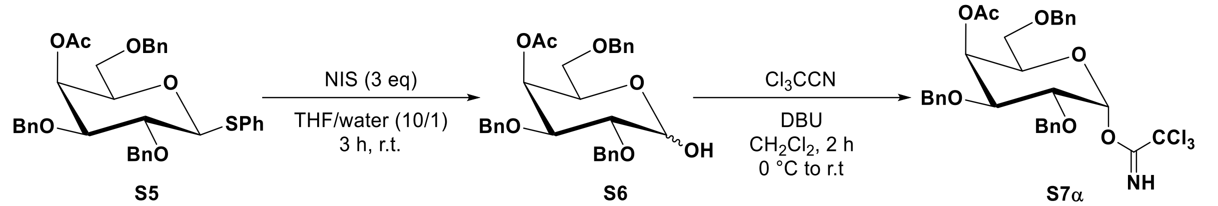

S5 S6 S7α

To a solution **S5**[3] (316 mg, 0.54 mmol) in THF (3 mL) and water (0.3 mL) were added N-iodosuccinimide (243 mg, 1.08 mmol) and 1M HCl 5 drops, and then stirred for 3 h at room temperature. The reaction mixture was quenched with with sat. aq. $NaHCO_3$ solution (10 mL) and DCM (10 mL). The organic layer was extracted with DCM (2 × 10 mL) and washed with brine (10 mL). The organic layer was dried over anhydrous Na_2SO_4, filtered and evaporated under reduced pressure for column chromatography purification (Elution: n-hexane/EtOAc = 4/1 to 2/1) and obtained as an inseparable α/β mixture **S6** (Rf: 0.15 in *n*-Hexane/ EtOAc = 3/1). Compound **S6** in dry DCM (10 mL) were added CCl_3CN (0.1 mL, 1 mmol) and DBU (0.03 mL, 0.02 mmol) at 0 °C. The dark solution was stirred at room temperature for 3 h, and then the reaction mixture was concentrated. The residue was purified by silica gel column chromatography (Elution: Toluene/EtOAc = 20/1 containing 1% Et_3N) to give **S7α** (179 mg, 0.28 mmol) as pale yellow oil (Rf: 0.20 in Toluene/EtOAc = 20/1). with 52% yield; ^1H NMR (600 MHz, Chloroform-*d*) δ 8.57 (s, 1H), 7.39 – 7.21 (m, 15H), 6.52 (d, *J* = 3.4 Hz, 1H), 5.72 (dd, *J* = 3.4, 1.3 Hz, 1H), 4.80 – 4.70 (m, 3H), 4.57 (d, *J* = 11.4 Hz, 1H), 4.53 (d, *J* = 11.8 Hz, 1H), 4.43 (d, *J* = 11.8 Hz, 1H), 4.30 (ddd, *J* = 7.1, 5.6, 1.4 Hz, 1H), 4.03 (dd, *J* = 10.0, 3.2 Hz, 1H), 3.96 (dd, *J* = 10.0, 3.5 Hz, 1H), 3.54 (dd, *J* = 9.7, 5.7 Hz, 1H), 3.47 (dd, *J* = 9.7, 7.1 Hz, 1H), 2.07 (s, 3H); ^{13}C NMR (151 MHz, Chloroform-*d*) δ 170.3, 161.3, 138.5, 138.0, 137.7, 128.6, 128.41, 128.36, 128.22, 128.16, 128.0, 127.7, 127.6, 127.5, 95.1 (C-1α, $^1J_{CH}$ = 180 Hz), 75.4, 75.0, 73.7, 73.3, 72.0, 70.6, 67.9, 67.6, 21.0; HRMS (ESI): $[M+Na]^+$ calcd for $C_{31}H_{32}Cl_3NO_7Na^+$ 658.1137, found 658.1141.

Preparation of 6- O-acetyl-2,3,4-tri-O-benzyl-α-D-galactopyranosyl trichloroacetimidate (S10α)

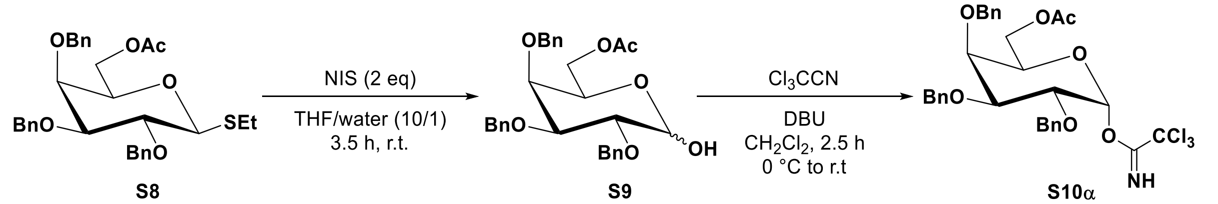

S8 S9 S10α

To a solution **S8**[4] (279 mg, 0.52 mmol) in THF (3 mL) and water (0.3 mL) were added N-iodosuccinimide (87.7 mg, 0.39 mmol) and 1M HCl 2 drops, and then stirred for 3 h at room temperature. The reaction mixture was quenched with with sat. aq. $NaHCO_3$ solution (10 mL) and DCM (10 mL). The organic layer was extracted with DCM (2 × 10 mL) and washed with brine (10 mL). The organic layer was dried over anhydrous Na_2SO_4, filtered and evaporated under reduced pressure for column chromatography purification (Elution: n-hexane/EtOAc = 6/1 to 2/1) and obtained as an inseparable α/β mixture **S8**

(Rf: 0.1 in *n*-Hexane/ EtOAc = 3/1). Compound **S8** in dry DCM (10 mL) were added CCl_3CN (0.1 mL, 1 mmol) and DBU (0.03 mL, 0.02 mmol) at 0 °C. The dark solution was stirred at room temperature for 3 h, and then the reaction mixture was concentrated. The residue was purified by silica gel column chromatography (Elution: Toluene/EtOAc = 20/1 containing 1% Et_3N) to give **S7α** (222 mg, 0.34 mmol) as white sold (Rf: 0.18 in Toluene/EtOAc = 20/1) with 67% yield; ^{1}H NMR (600 MHz, Chloroform-*d*) δ 8.56 (s, 1H), 7.42 – 7.23 (m, 15H), 6.55 (d, *J* = 3.5 Hz, 1H), 5.00 (d, *J* = 11.4 Hz, 1H), 4.88 (d, *J* = 11.8 Hz, 1H), 4.81 – 4.73 (m, 3H), 4.65 (d, *J* = 11.4 Hz, 1H), 4.26 (dd, *J* = 10.1, 3.5 Hz, 1H), 4.15 – 4.09 (m, 3H), 4.03 (dd, *J* = 10.0, 2.8 Hz, 1H), 3.95 (d, *J* = 2.7 Hz, 1H), 1.95 (s, 3H). ^{13}C NMR (151 MHz, Chloroform-*d*) δ 170.7, 161.3, 138.5, 138.4, 138.2, 128.6, 128.5, 128.4, 128.0, 127.81, 127.79, 127.7, 127.6, 95.1 (C-1α, $^1J_{CH}$ = 179 Hz), 78.2, 76.0, 74.8, 74.5, 73.6, 73.1, 71.5, 63.4, 20.9; HRMS (ESI): $[M+Na]^+$ calcd for $C_{31}H_{32}Cl_3NO_7Na^+$ 658.1137, found 658.1140.

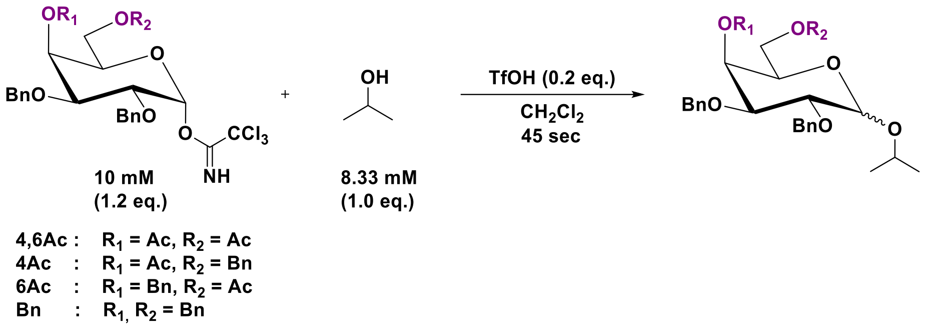

4,6Ac

Temp.	α ratio (%)	β ratio (%)	Total yield
-50	59,3	40,7	78,4
-30	72,0	28,0	88,5
-10	79,6	20,4	89,5
10	88,0	12,0	92,2
30	89,9	10,1	83,9

4Ac

Temp.	α ratio (%)	β ratio (%)	Total yield
-50	52,8	47,2	97,0
-30	69,0	31,0	100
-10	76,7	23,3	96,3
10	82,3	17,7	93,6
30	84,8	15,2	81,6

6Ac

Temp.	α ratio (%)	β ratio (%)	Total yield
-50	20,3	79,7	94,3
-30	27,4	72,6	95,0
-10	34,6	65,4	96,3
10	41,8	58,2	93,5
30	47,9	52,1	79,6

Bn

Temp.	α ratio (%)	β ratio (%)	Total yield
-50	19,3	80,7	98,2
-30	29,6	70,4	99,7
-10	41,0	59,0	96,4
10	46,4	53,6	96,9
30	51,4	48,6	78,0

Figure S12: Reactions of galactose building blocks to assess the impact of different protecting group combinations (Bn=benzyl, Ac=acetyl) on the stereochemical outcome of the glycosylation reaction. All reactions including **Bn** data were performed by following same methodology given in ref. 5.

Analysis Section:

The reactions were monitored using HPLC. The HPLC system used was a Knauer Platin Blue system, equipped with a UV detector (254 nm). The column used was YMC-Pack Diol Normal Phase diol column (DN12S05-2546WT) with particle size of 5 µm. The column has an I.D. of 4.6 mm and length of 250 mm. The column was housed inside a column oven, and was maintained at 20 °C for all analysis. The mobile phase was gradient mixture of HPLC grade ethyl acetate and hexane, which was pumped with a constant flowrate of 1 mL/min. The gradient system of the mobile phase was developed and programmed into the HPLC.

HPLC Method

Time [min]	Flow [ml/min]	EtOAc [%]	Hexane [%]
0	1	2	98
14	1	25	75
16	1	70	30
18	1	70	30
19	1	2	98
22	1	2	98

Characterization of Products

Isopropyl 4,6-di-O-acetyl-2,3-di-O-benzyl-αβ-D-galactopyranoside (P1)

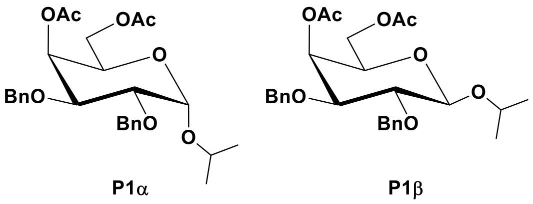

P1α P1β

α anomer - ^{1}H NMR (400 MHz, Chloroform-*d*) δ 7.45 – 7.20 (m, 10H), 5.56 (s, H-4, 1H), 4.93 (d, H-1α, *J* = 3.8 Hz, 1H), 4.81 (d, *J* = 12.0 Hz, 1H), 4.73 (d, *J* = 10.8 Hz, 1H), 4.62 (d, *J* = 12.0 Hz, 1H), 4.55 (d, *J* = 10.9 Hz, 1H), 4.21 (t, *J* = 6.5 Hz, 1H), 4.17 – 4.01 (m, 2H), 3.96 (dd, *J* = 9.9, 3.3 Hz, 1H), 3.88 (hept, *i*Pr, *J* = 6.2 Hz, 1H), 3.76 (dd, *J* = 9.7, 3.6 Hz, 1H), 2.11 (s, Ac, 3H), 2.05 (s, Ac, 3H), 1.24 (d, *i*Pr, *J* = 6.4 Hz, 3H), 1.20 (d, *i*Pr, *J* = 6.1 Hz, 3H); ^{13}C NMR (101 MHz, Chloroform-*d*) δ 170.71, 170.55, 138.73, 138.31, 128.47, 128.44, 128.13, 127.99, 127.81, 127.73, 96.18 (C-1α, $^{1}J_{CH}$ = 170 Hz), 76.31, 75.65, 73.57, 72.47, 70.39, 68.11, 66.69, 62.76, 23.21, 21.63, 21.05, 20.89; β anomer - ^{1}H NMR (400 MHz, Chloroform-*d*) δ 7.43 – 7.20 (m, 10H), 5.48 (d, H-4, *J* = 3.0 Hz, 1H), 4.90 (d, *J* = 10.8 Hz, 1H), 4.73 (dd, *J* = 13.8, 11.1 Hz, 2H), 4.53 (d, *J* = 11.4 Hz, 1H), 4.45 (d, H-1β, *J* = 6.8 Hz, 1H), 4.23 – 4.10 (m, 2H), 4.00 (hept, *i*Pr , *J* = 6.1 Hz, 1H), 3.75 (t, *J* = 6.7 Hz, 1H), 3.65 – 3.52 (m, 2H), 2.14 (s, Ac, 3H), 2.07 (s, Ac, 3H), 1.30 (d, *i*Pr, *J* = 6.2 Hz, 3H), 1.25 (d, *i*Pr, *J* = 6.2 Hz, 3H); ^{13}C NMR (101 MHz, Chloroform-*d*) δ 170.71, 170.67, 138.74, 137.97, 128.47, 128.42, 128.23, 128.16, 127.84, 127.76, 102.65 (C-1β, $^{1}J_{CH}$ = 159 Hz), 79.39, 78.95, 75.52, 73.26, 72.40, 70.75, 66.73, 62.28, 23.67, 22.44, 21.10, 20.91; HRMS (ESI): [M+Na] $^{+}$ calcd for $C_{27}H_{34}O_8Na^+$ 509.2146, found 509.2150.

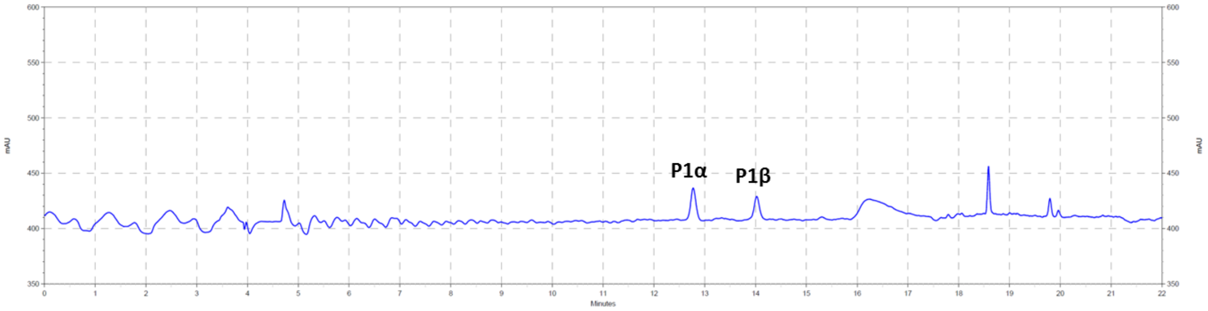

Figure S13: HPLC spectrum of **P1** (Galactosylation of **S4α** with *i*PrOH in DCM at -50°C).

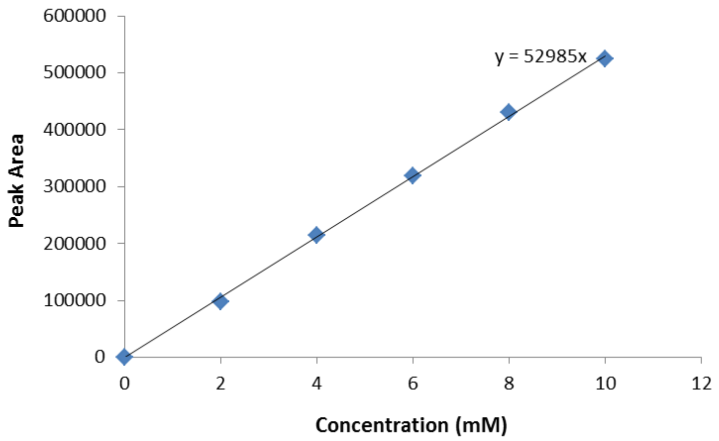

Figure S14: HPLC calibration curve of **P1**.

Isopropyl 4-O-acetyl-2,3,6-tri-O-benzyl-αβ-D-galactopyranoside (P2)

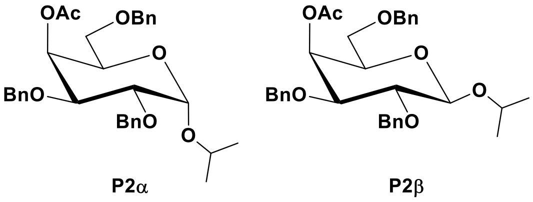

P2α **P2β**

α anomer - ^{1}H NMR (400 MHz, Chloroform-*d*) δ 7.39 – 7.26 (m, 15H), 5.63 (d, H-4, J = 1.9 Hz, 1H), 4.94 (d, H-1α, J = 3.8 Hz, 1H), 4.81 (d, J = 12.0 Hz, 1H), 4.76 (d, J = 10.8 Hz, 1H), 4.62 (d, J = 12.1 Hz, 1H), 4.58 – 4.50 (m, 2H), 4.45 (d, J = 11.9 Hz, 1H), 4.18 (t, J = 6.4 Hz, 1H), 3.96 (dd, J = 10.1, 3.3 Hz, 1H), 3.90 (hept, *i*Pr, J = 6.1 Hz, 1H), 3.75 (dd, J = 10.0, 3.8 Hz, 1H), 3.52 – 3.41 (m, 2H), 2.05 (s, Ac, 3H), 1.25 (d, *i*Pr, J = 6.4 Hz, 3H), 1.20 (d, *i*Pr, J = 6.1 Hz, 3H);
^{13}C NMR (151 MHz, Chloroform-*d*) 170.50, 138.85, 138.48, 137.94, 128.56, 128.44, 128.42, 128.19, 128.00, 127.99, 127.89, 127.75, 127.67, 95.99 (C-1α, $^{1}J_{CH}$ = 170 Hz), 76.61, 75.76, 73.75, 73.50, 72.35, 69.93, 68.71, 68.48, 67.74, 23.33, 21.49, 21.09;
β anomer - ^{1}H NMR (400 MHz, Chloroform-*d*) δ 7.42 – 7.18 (m, 15H), 5.57 (d, H-4, J = 2.4 Hz, 1H), 4.89 (d, J = 10.7 Hz, 1H), 4.76 (d, J = 11.4 Hz, 1H), 4.70 (d, J = 10.7 Hz, 1H), 4.56 (d, J = 11.8 Hz, 1H), 4.53 – 4.42 (m, 3H), 4.00 (hept, *i*Pr, J = 6.3 Hz, 1H), 3.71 (t, J = 6.4 Hz, 1H), 3.61 – 3.49 (m, 4H), 2.08 (s, Ac, 3H), 1.30 (d, *i*Pr, J = 6.2 Hz, 3H), 1.24 (d, *i*Pr, J = 6.1 Hz, 3H); ^{13}C NMR (101 MHz, Chloroform-*d*) δ 170.64, 138.83, 138.11, 137.86, 128.60, 128.44, 128.40, 128.22, 128.21, 128.13, 127.98, 127.78, 127.71, 102.58 (C-1β, $^{1}J_{CH}$ = 159 Hz), 79.63, 79.11, 77.48, 77.16, 76.84, 75.49, 73.84, 72.97, 72.26, 68.47, 67.11, 23.76, 22.39, 21.15; HRMS (ESI): [M+Na]$^{+}$ calcd for $C_{32}H_{38}O_7Na^+$ 557.2510, found 557.2514.

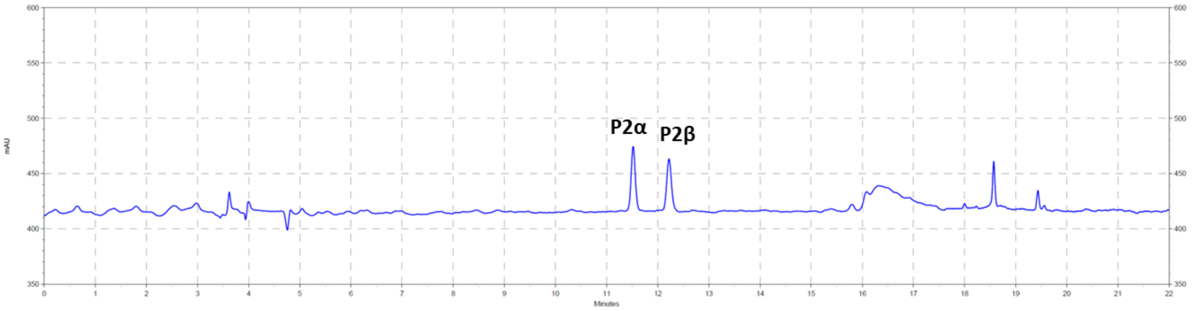

Figure S15: HPLC spectrum of **P2** (Galactosylation of **S7α** with *i*PrOH in DCM at -50°C).

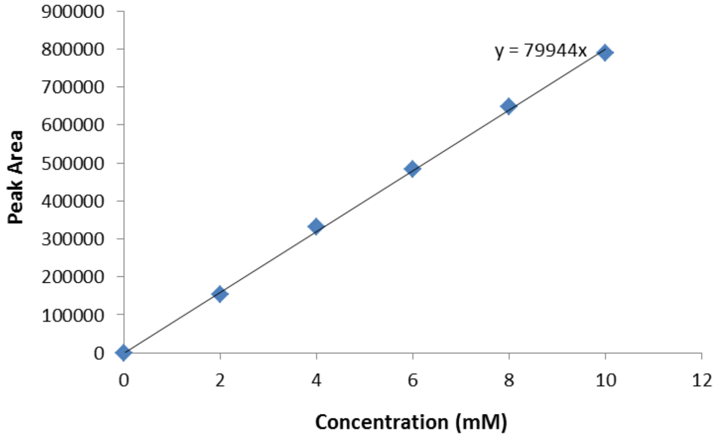

Figure S16: HPLC calibration curve of **P2**.

Isopropyl 6-O-acetyl-2,3,4-tri-O-benzyl-αβ-D-galactopyranoside (P3)

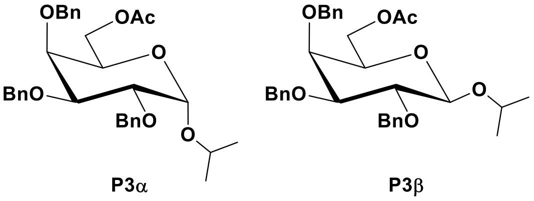

P3α **P3β**

α anomer - ^{1}H NMR (400 MHz, Chloroform-*d*) δ 7.44 – 7.24 (m, 15H), 4.97 (d, *J* = 11.5 Hz, 1H), 4.92 (d, H-1α, *J* = 3.8 Hz, 1H), 4.90 (d, *J* = 11.5 Hz, 1H), 4.81 (d, *J* = 12.0 Hz, 1H), 4.75 (d, *J* = 11.6 Hz, 1H), 4.67 (d, *J* = 12.0 Hz, 1H), 4.62 (d, *J* = 11.6 Hz, 1H), 4.11 (dd, *J* = 11.0, 7.2 Hz, 1H), 4.06 – 3.91 (m, 4H), 3.91 – 3.81 (m, 2H), 1.97 (s, Ac, 3H), 1.21 (d, *i*Pr, *J* = 6.4 Hz, 3H), 1.18 (d, *i*Pr, *J* = 6.1 Hz, 3H); ^{13}C NMR (151 MHz, Chloroform-*d*) δ 170.74, 139.03, 138.76, 138.48, 128.57, 128.54, 128.49, 128.47, 128.16, 127.88, 127.82, 127.66, 127.60, 95.88, 79.37, 76.66, 74.89, 74.65, 73.67, 73.46, 69.73, 68.39, 63.84, 23.21, 21.57, 20.93; β anomer - 1H NMR (400 MHz, Chloroform-*d*) δ 7.44 – 7.20 (m, 15H), 4.96 (dd, *J* = 11.2, 3.8 Hz, 2H), 4.82 (d, J = 11.8 Hz, 1H), 4.75 (dd, *J* = 11.4, 3.8 Hz, 2H), 4.67 (d, H-1β, *J* = 11.7 Hz, 1H), 4.41 (d, J = 7.7 Hz, 1H), 4.22 (dd, J = 11.1, 6.6 Hz, 1H), 4.08 – 3.91 (m, 2H), 3.82 (dd, *J* = 9.8, 7.7 Hz, 1H), 3.76 (d, *J* = 2.9 Hz, 1H), 3.56 – 3.42 (m, 2H), 1.95 (s, Ac, 3H), 1.27 (d, *i*Pr, *J* = 6.2 Hz, 3H), 1.23 (d, *i*Pr, *J* = 6.1 Hz, 3H); ^{13}C NMR (101 MHz, Chloroform-*d*) δ 170.70, 138.90, 138.67, 138.41, 128.74, 128.54, 128.43, 128.39, 128.36, 127.84, 127.76, 127.70, 127.69, 102.68 (C-1β, $^{1}J_{CH}$ = 160 Hz), 82.58, 79.68, 75.34, 74.33, 73.61, 73.04, 72.56, 72.04, 63.24, 23.73, 22.33, 20.94; HRMS (ESI): $[M+Na]^+$ calcd for $C_{32}H_{38}O_7Na^+$ 557.2510, found 557.2514.

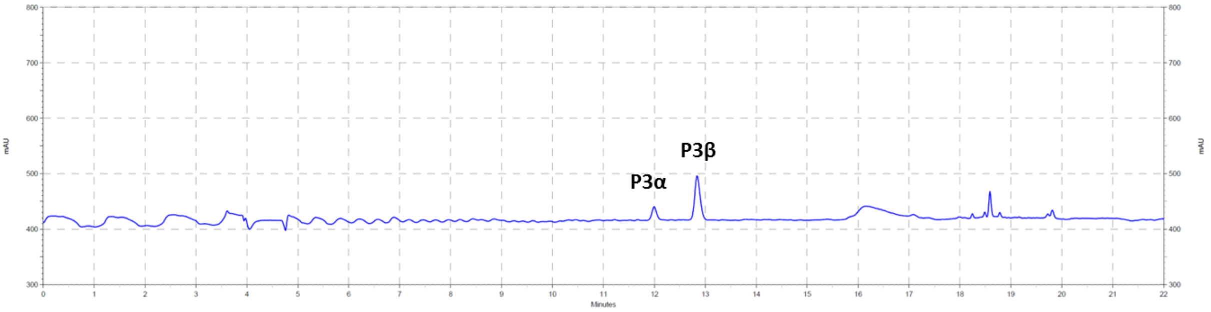

Figure S17: HPLC spectrum of **P3** (Galactosylation of **S10α** with *i*PrOH in DCM at -50°C).

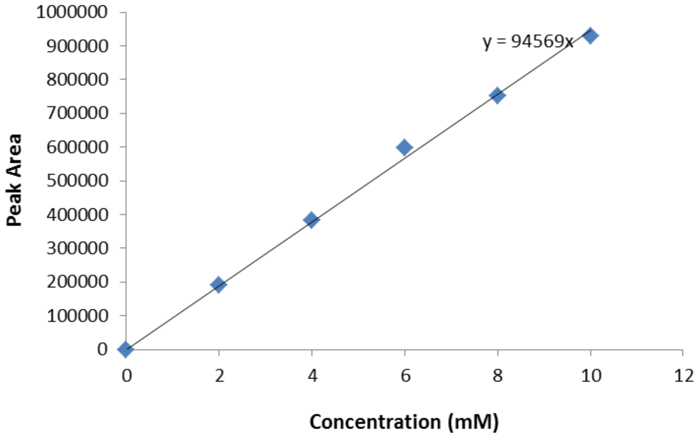

Figure S18: HPLC calibration curve of **P3**.

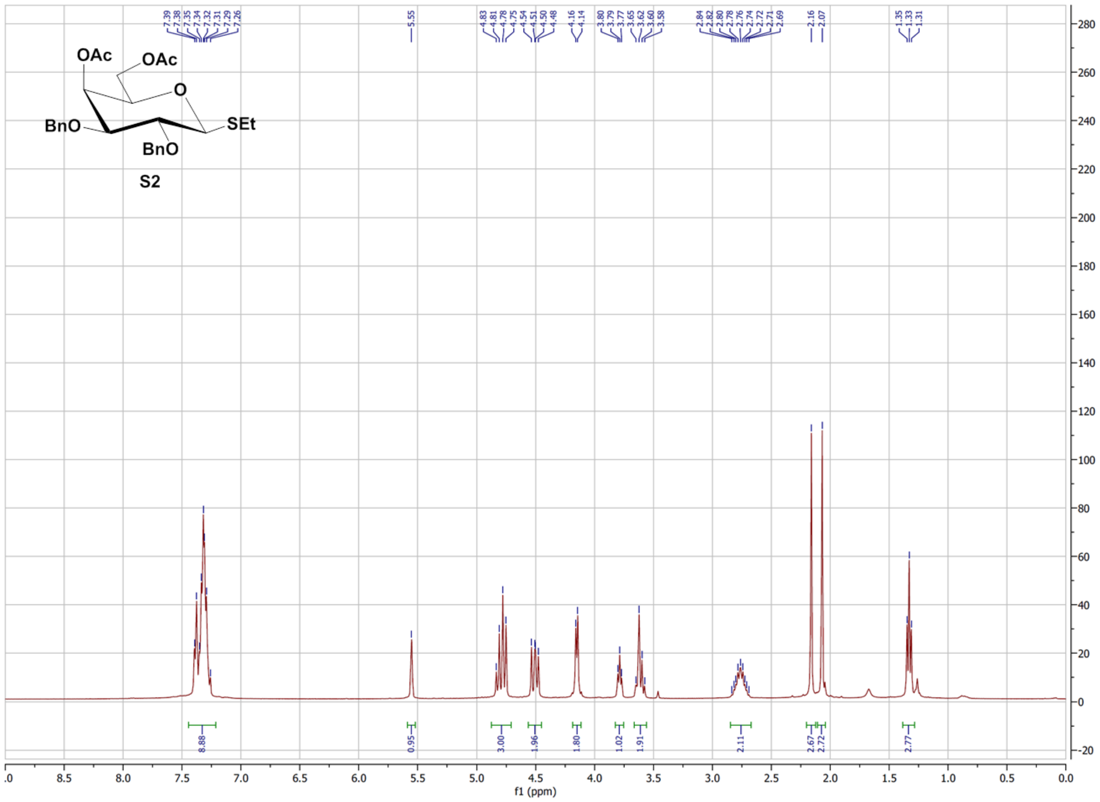

Figure S19: ^{1}H NMR spectrum of **S2**.

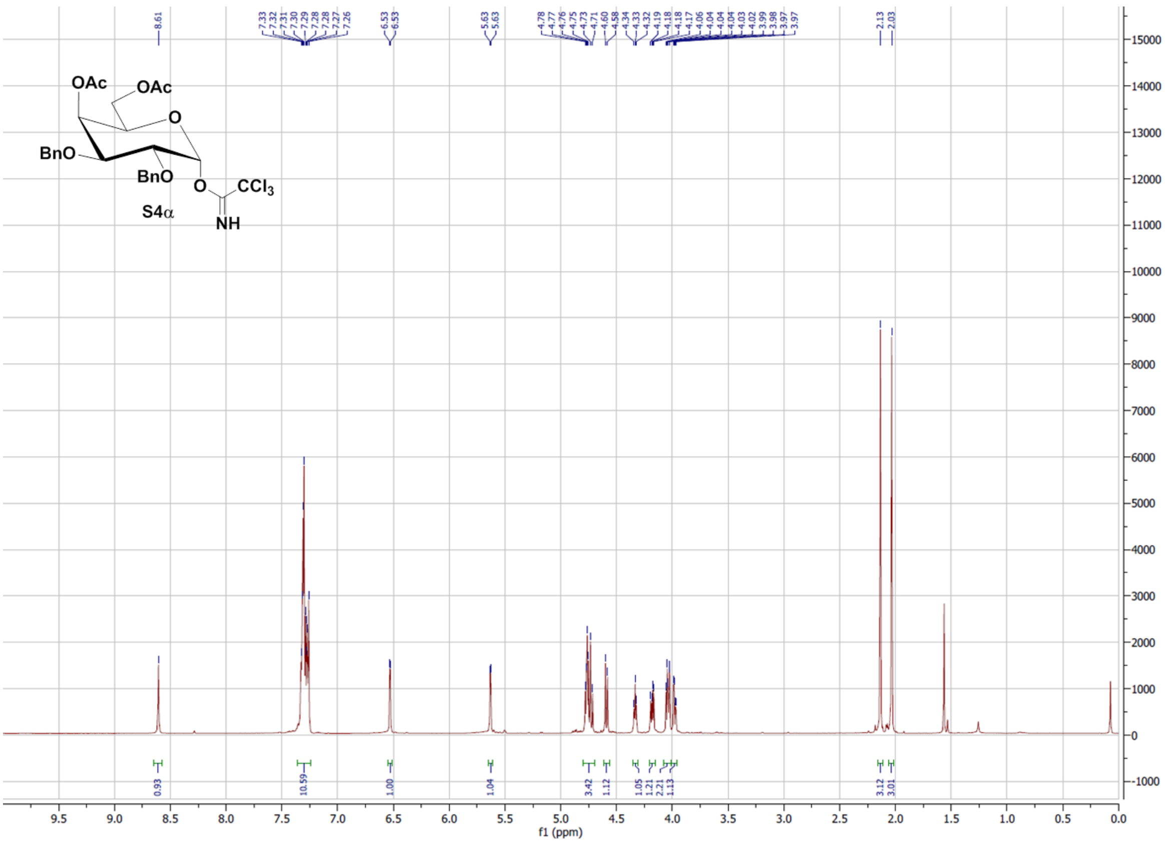

Figure S20: ^{1}H NMR spectrum of **S4α**.

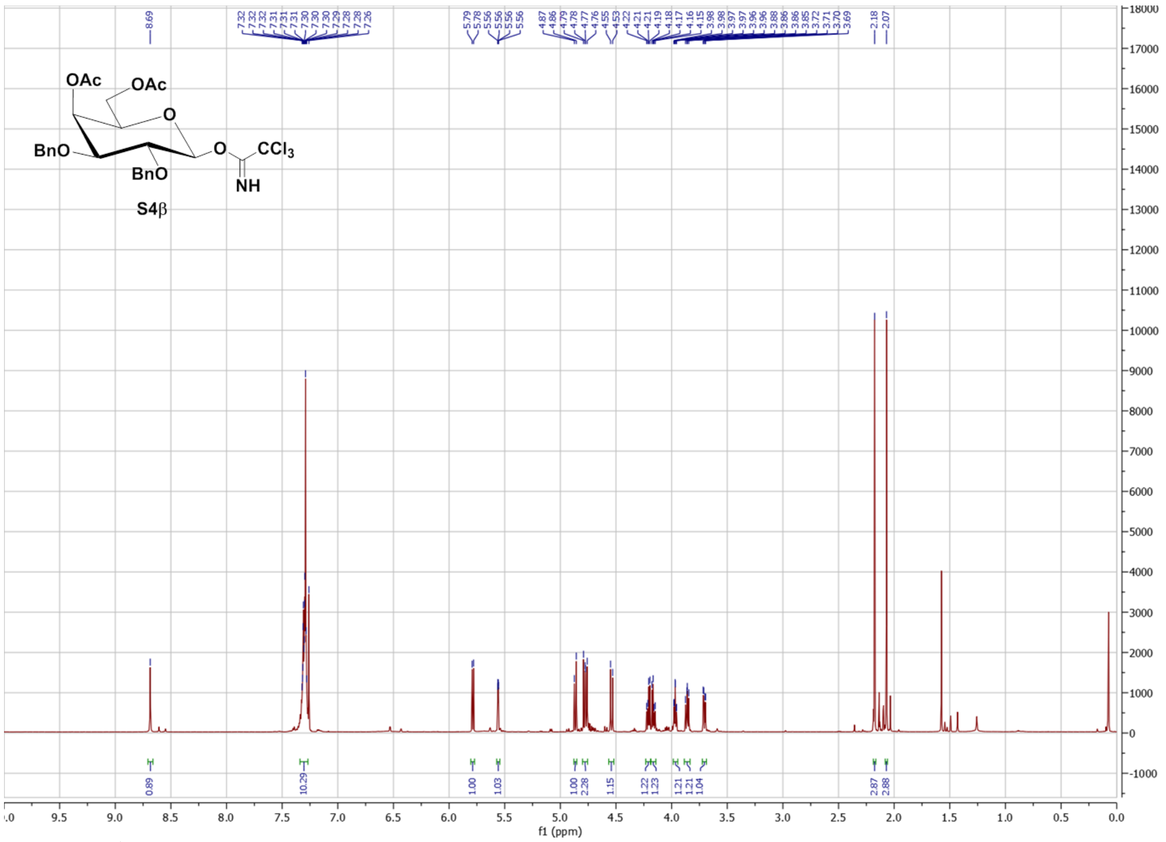

Figure S21: ^{1}H NMR spectrum of **S4β**.

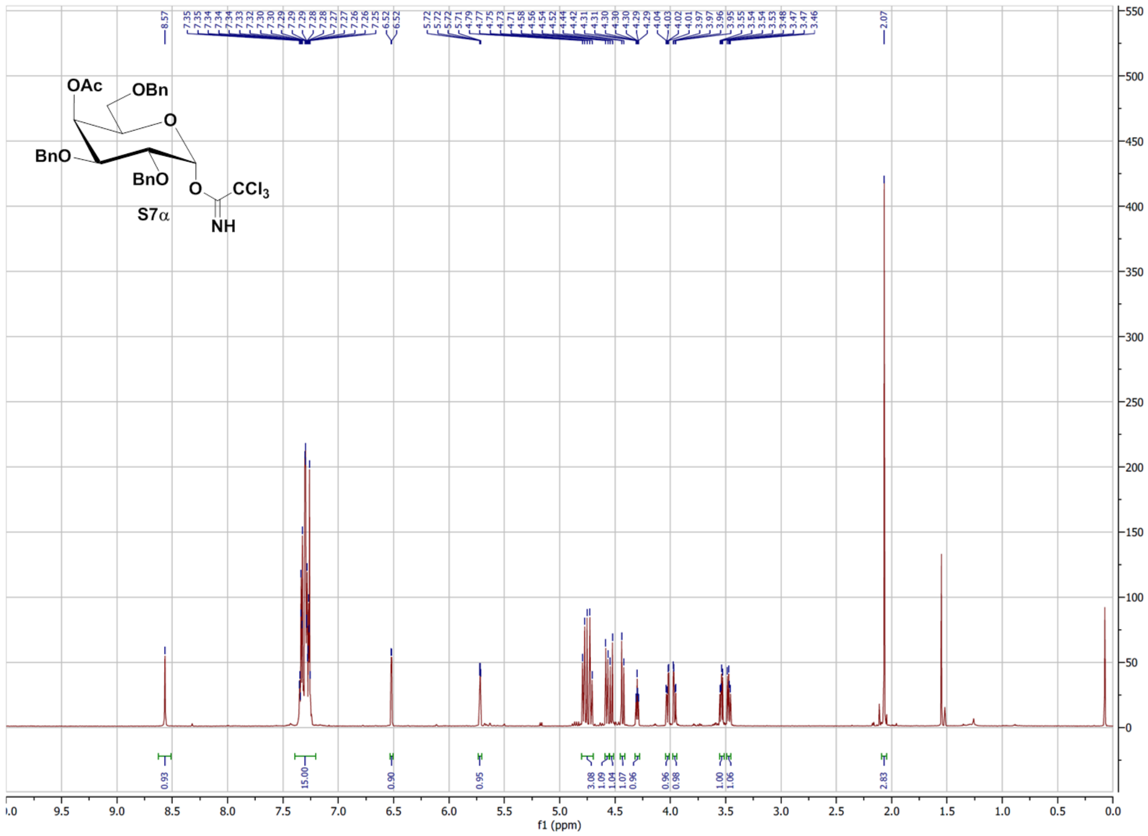

Figure S22: ^{1}H NMR spectrum of **S7α**.

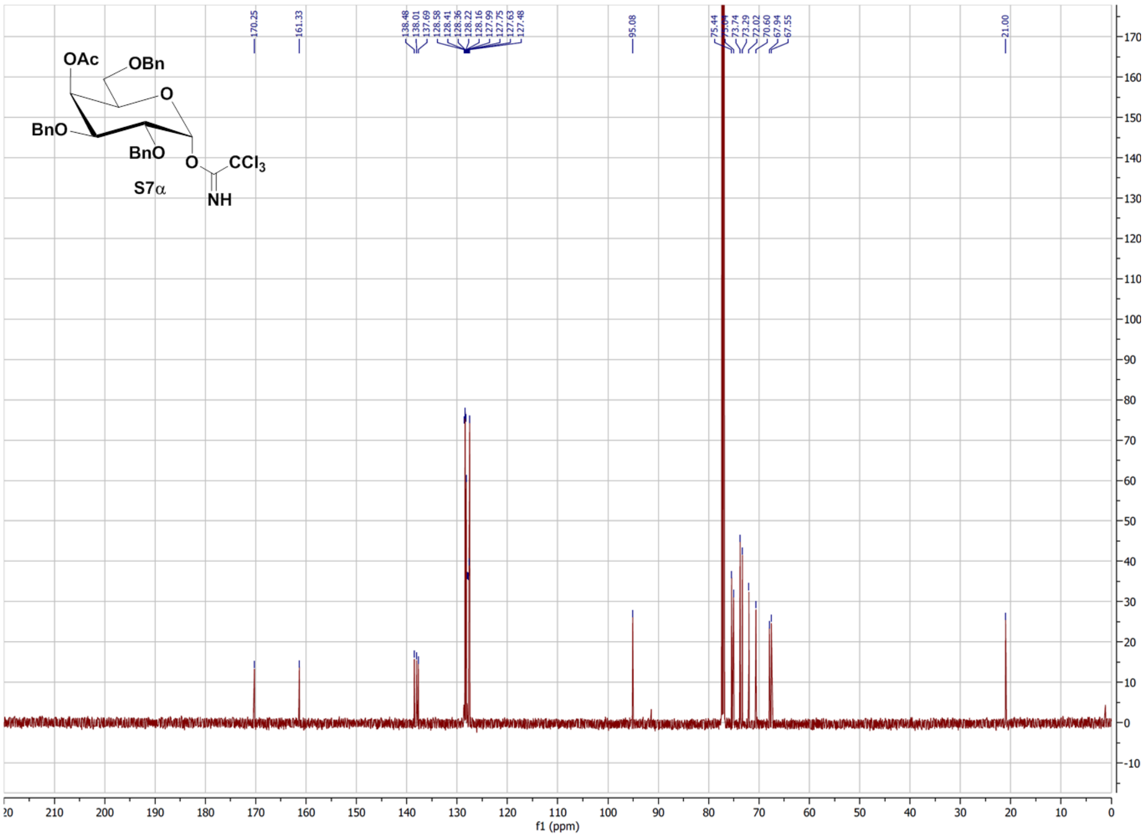

Figure S23: ^{13}C NMR spectrum of **S7α**.

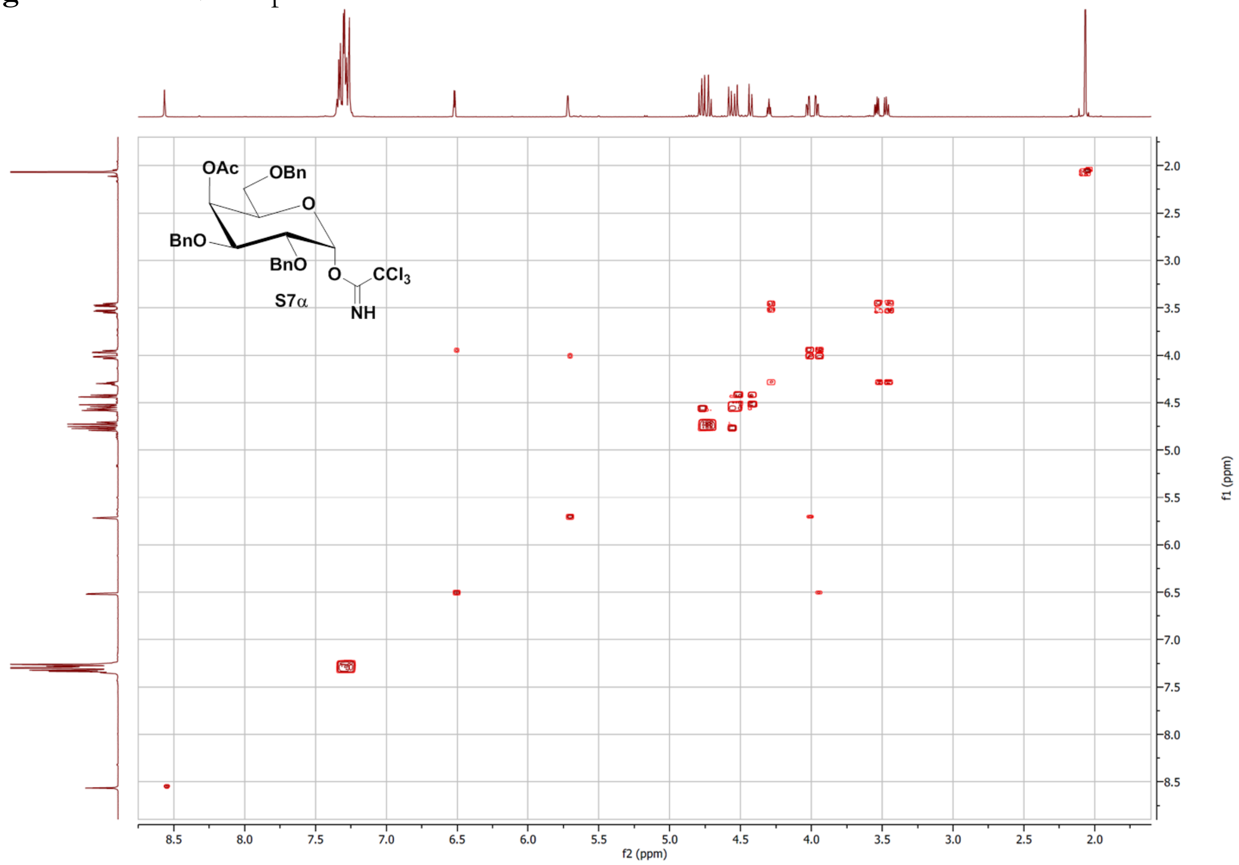

Figure S24: COSY NMR spectrum of **S7α**.

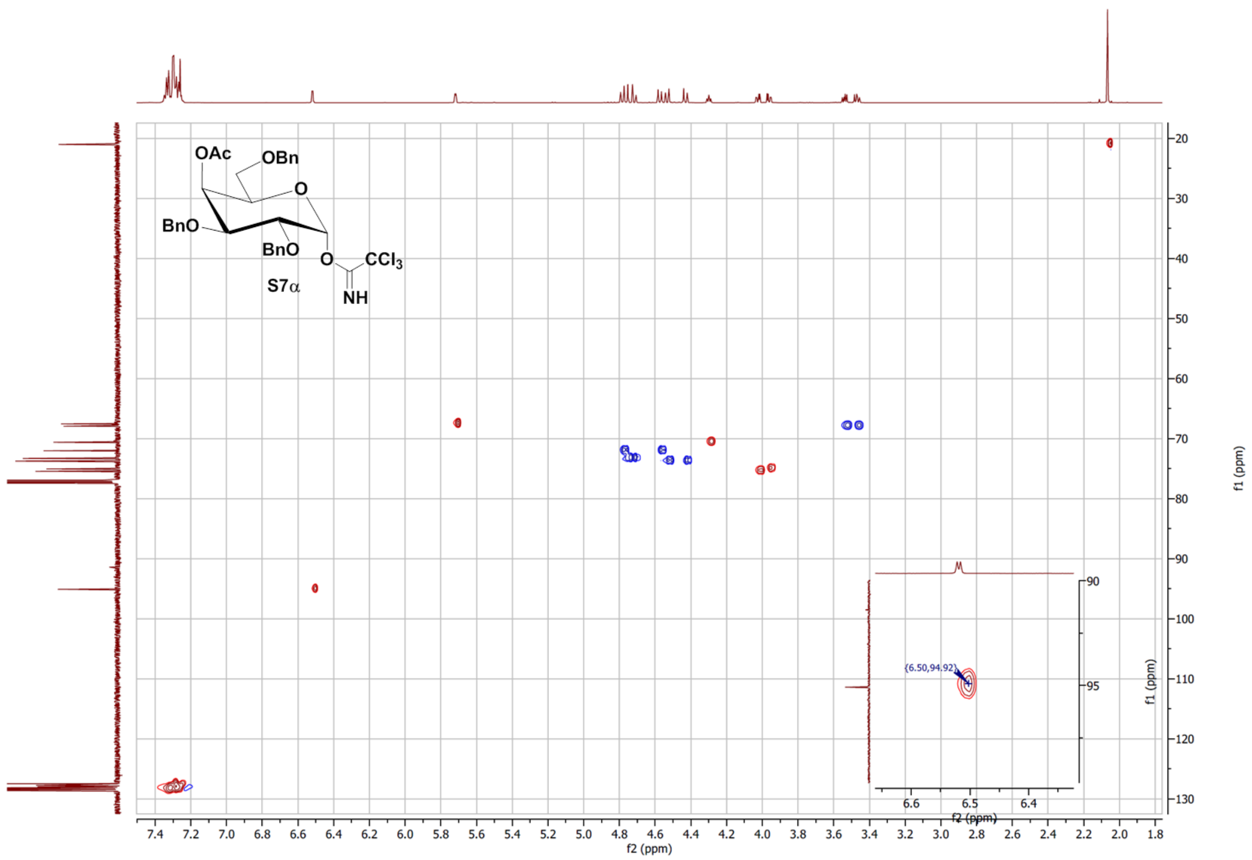

Figure S25: HSQC NMR spectrum of **S7α**.

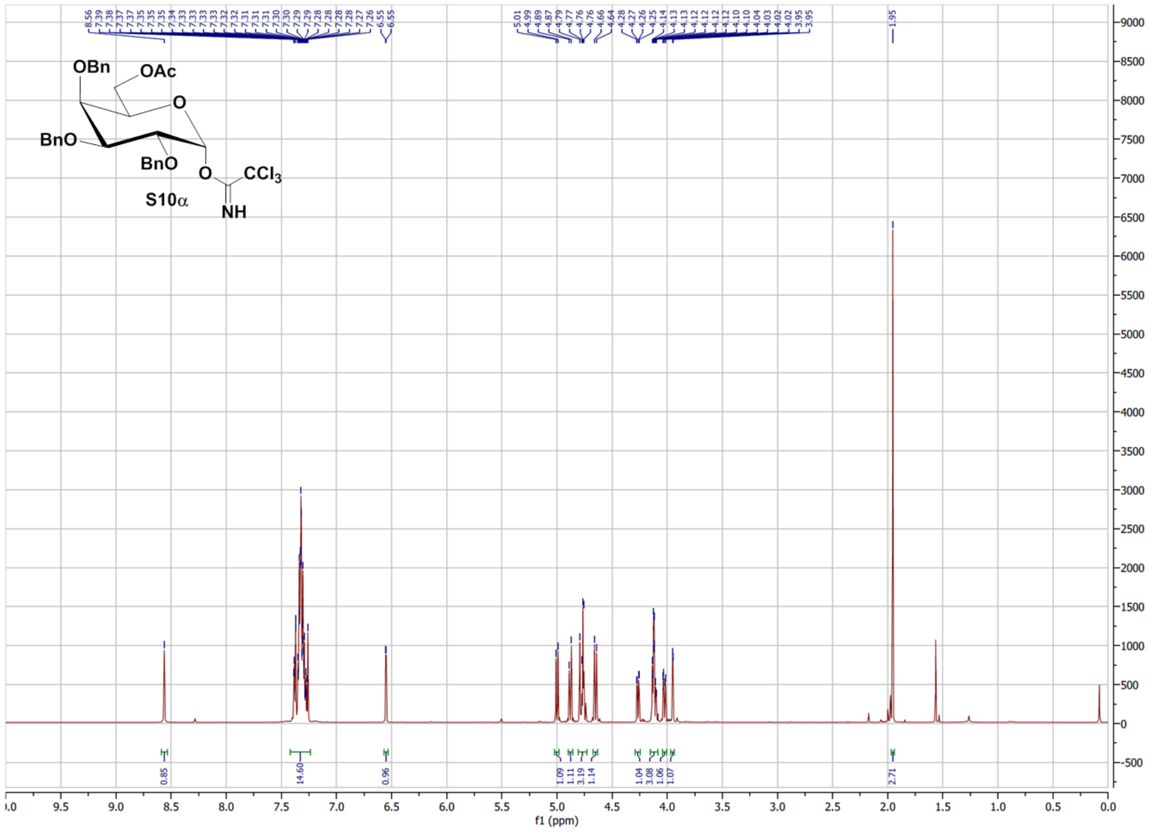

Figure S26: ^{1}H NMR spectrum of **S10α**.

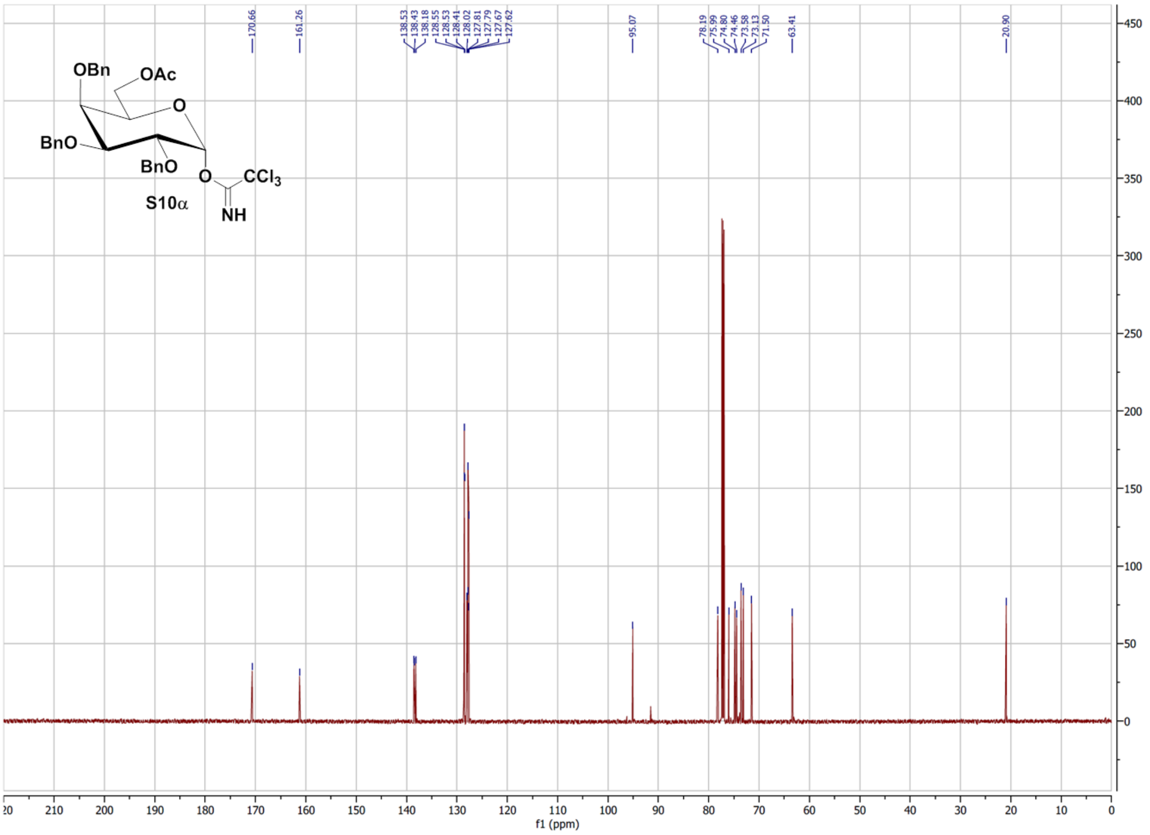

Figure S27: ^{13}C NMR spectrum of **S10α**.

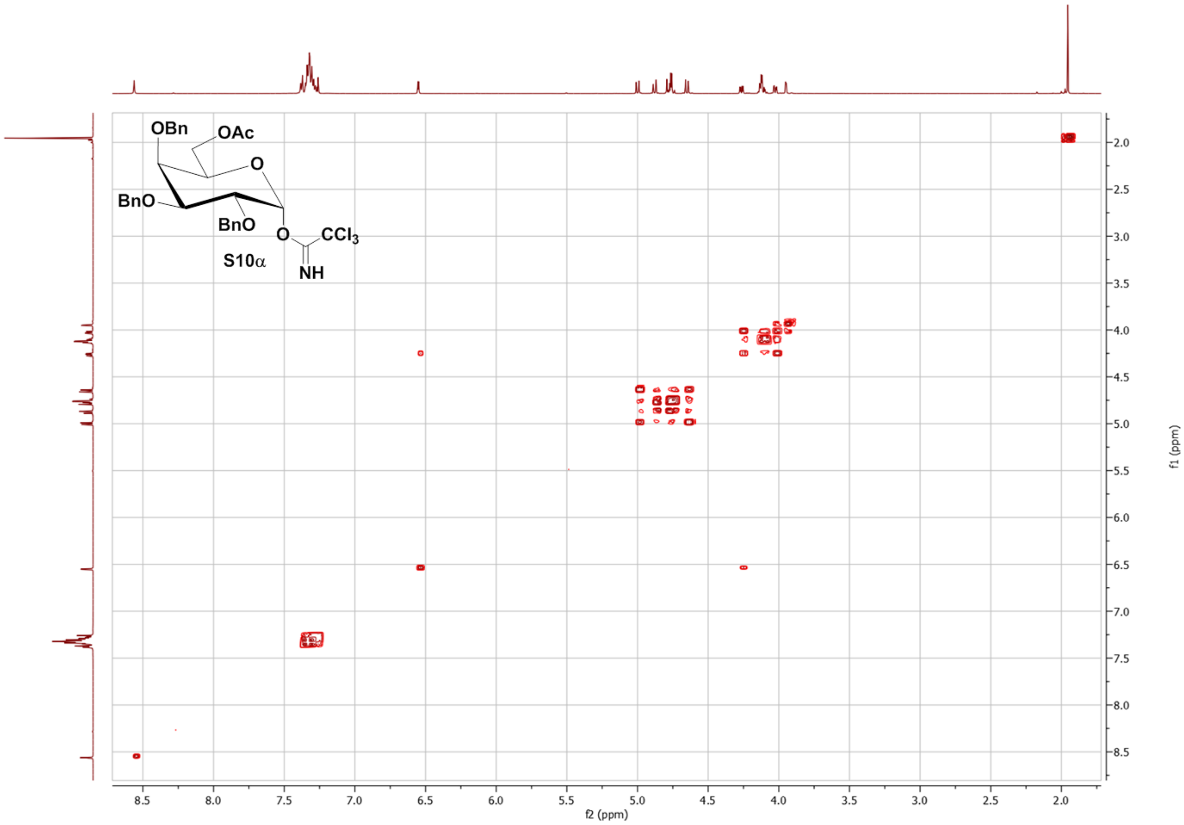

Figure S28: COSY NMR spectrum of **S10α**.

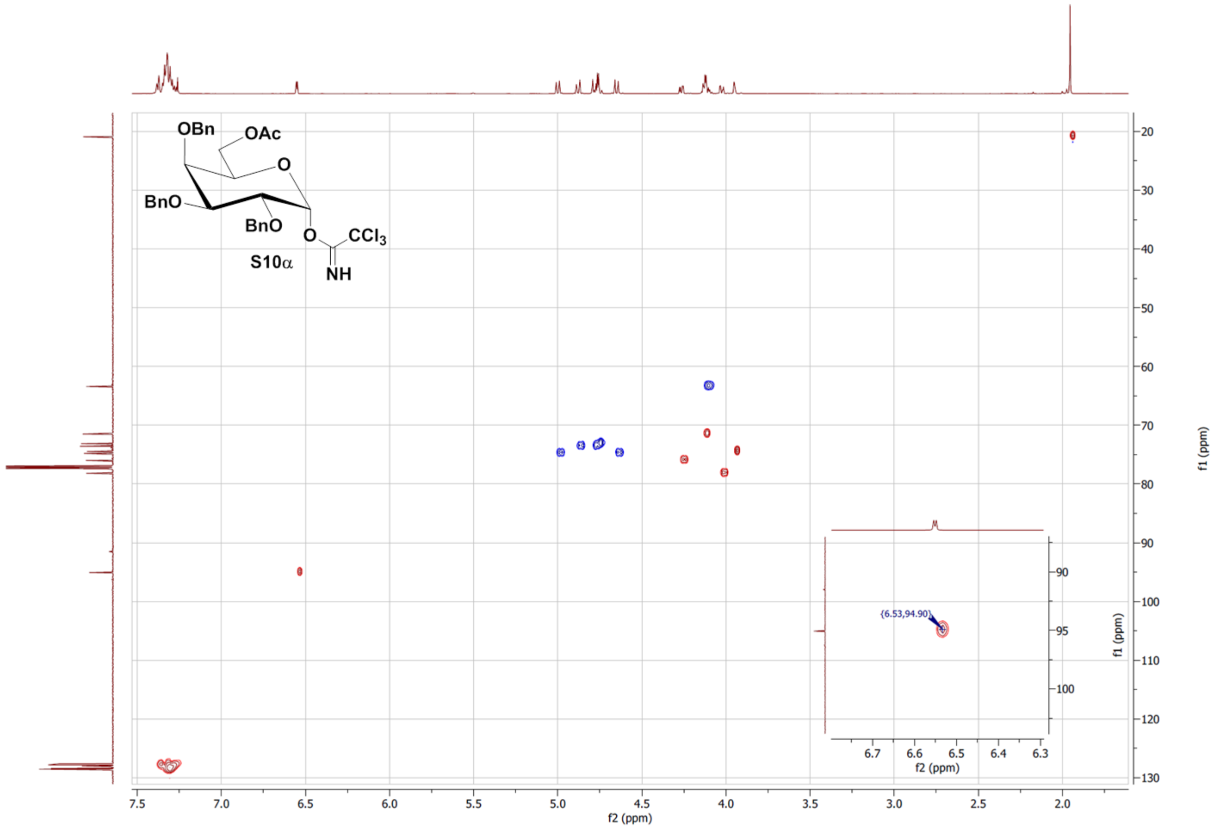

Figure S29: HSQC NMR spectrum of **S10α**.

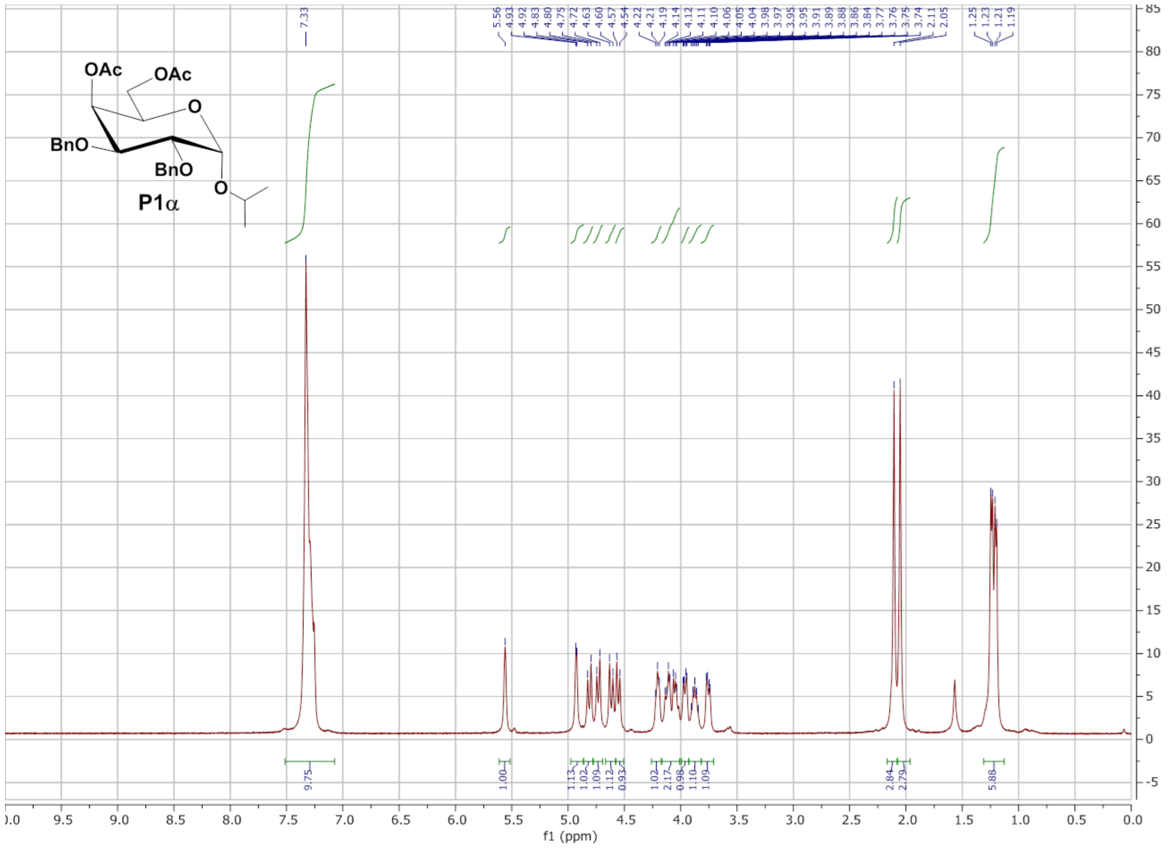

Figure S30: ^{1}H NMR spectrum of **P1α**.

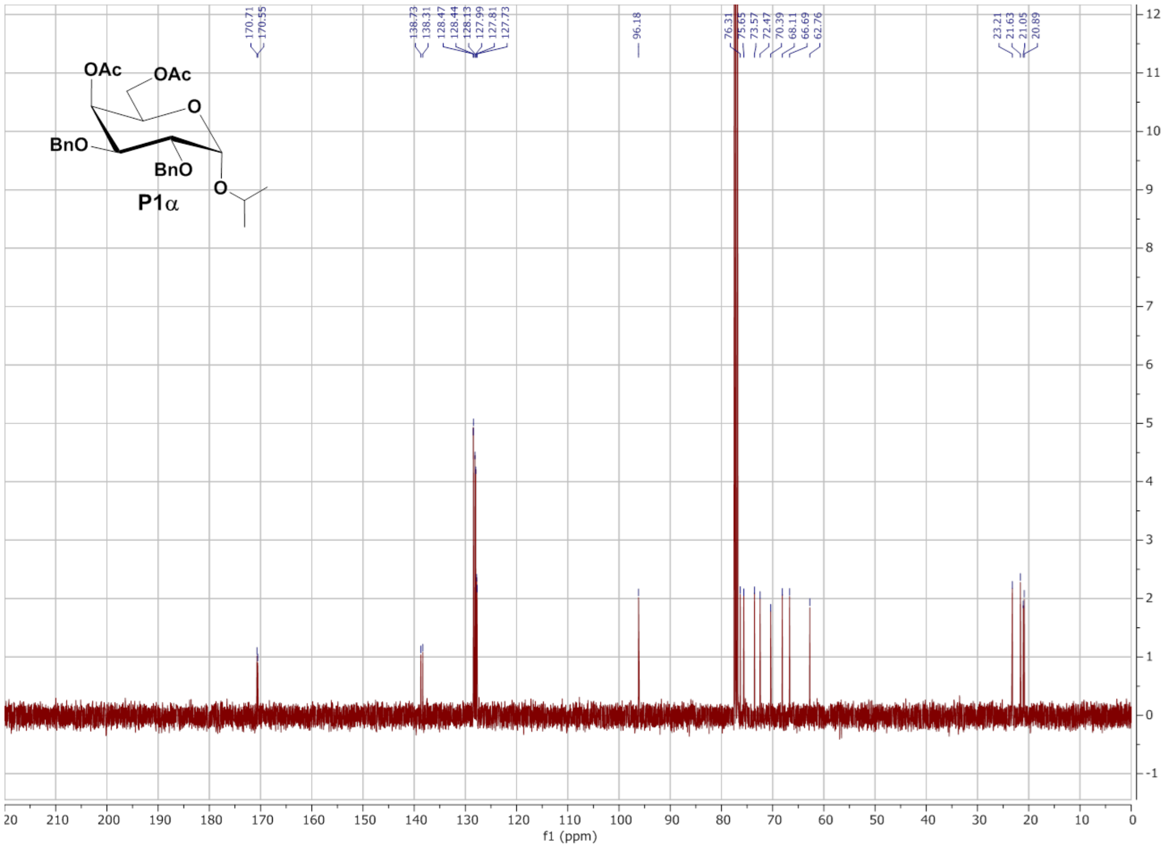

Figure S31: ^{13}C NMR spectrum of **P1α**.

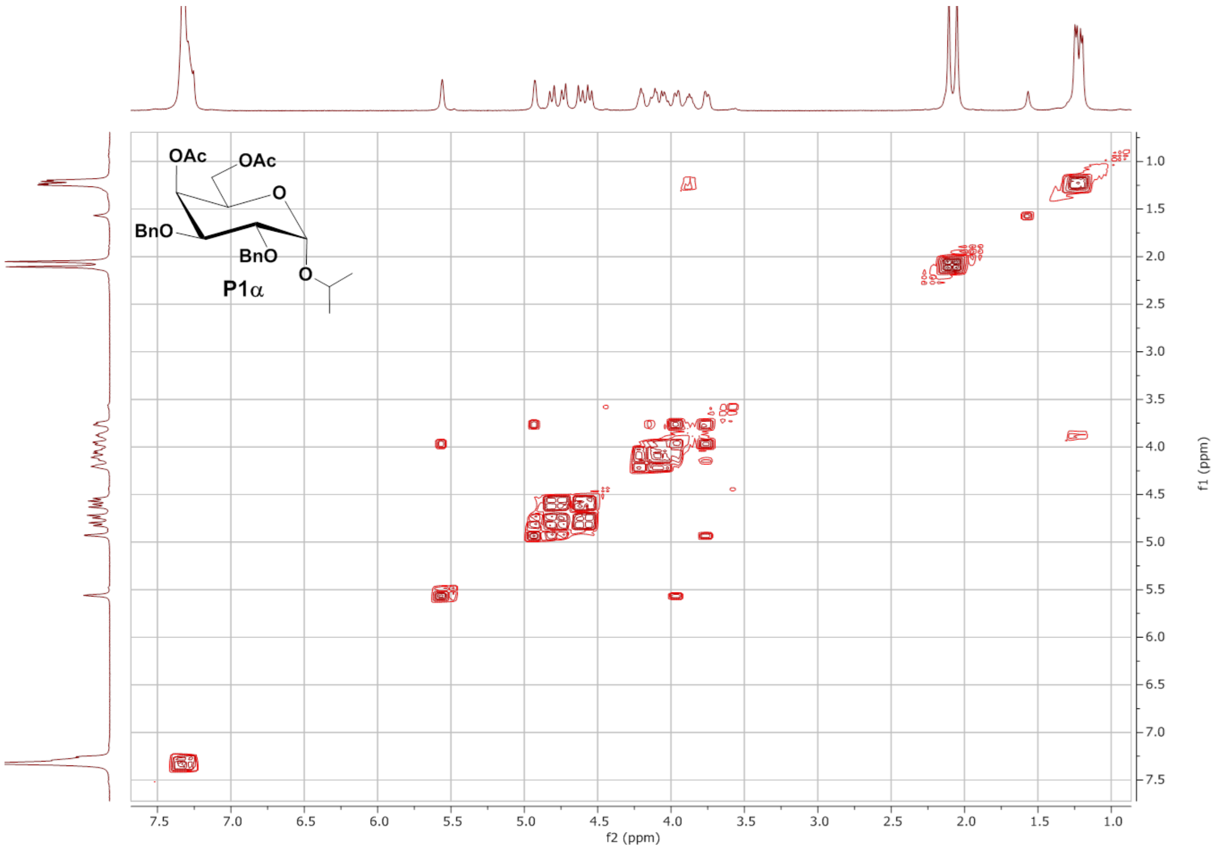

Figure S32: COSY NMR spectrum of **P1α**.

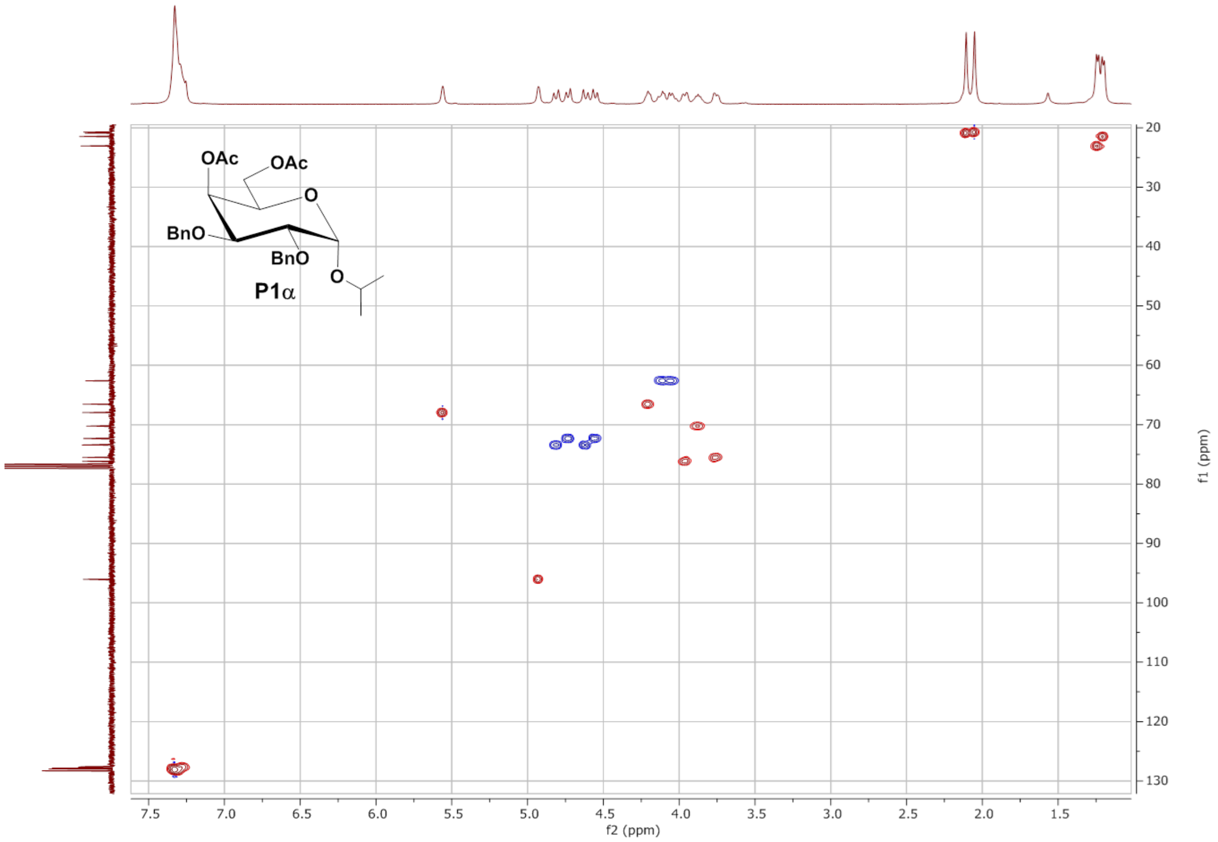

Figure S33: HSQC NMR spectrum of **P1α**.

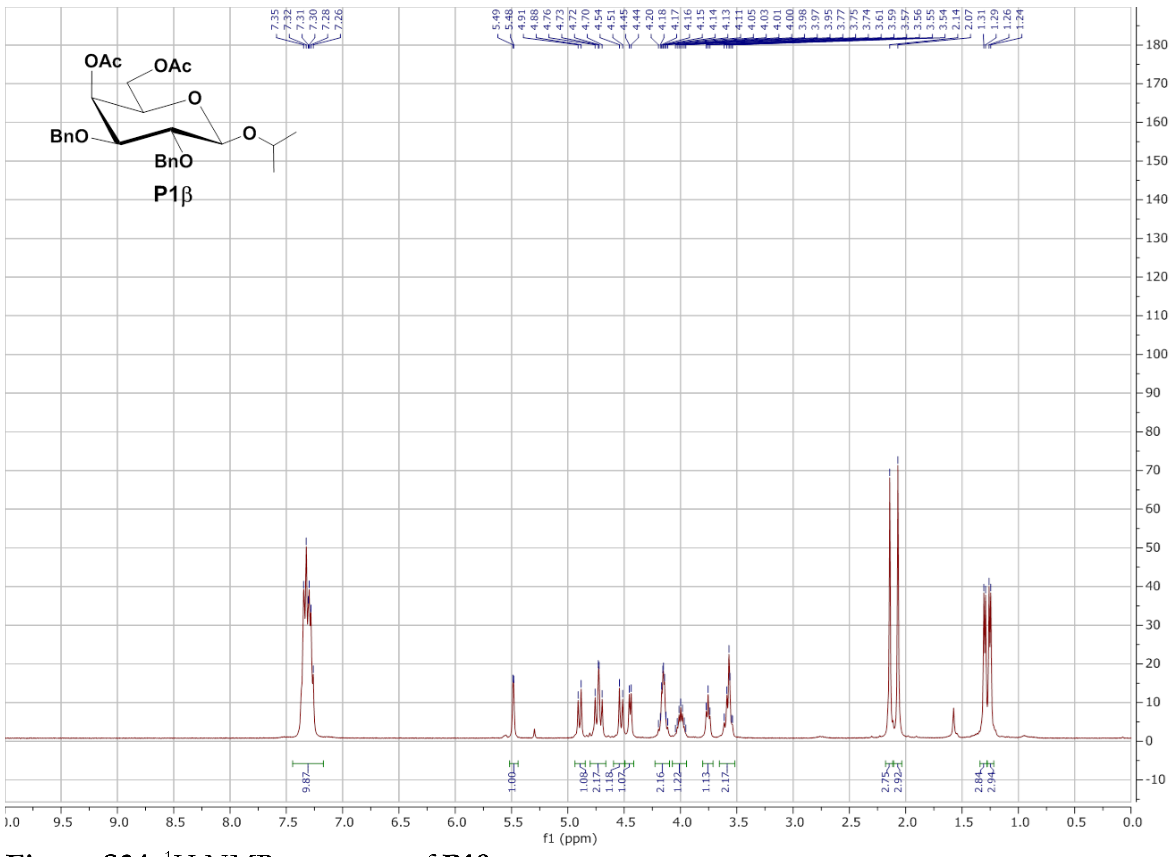

Figure S34: ^{1}H NMR spectrum of **P1β**.

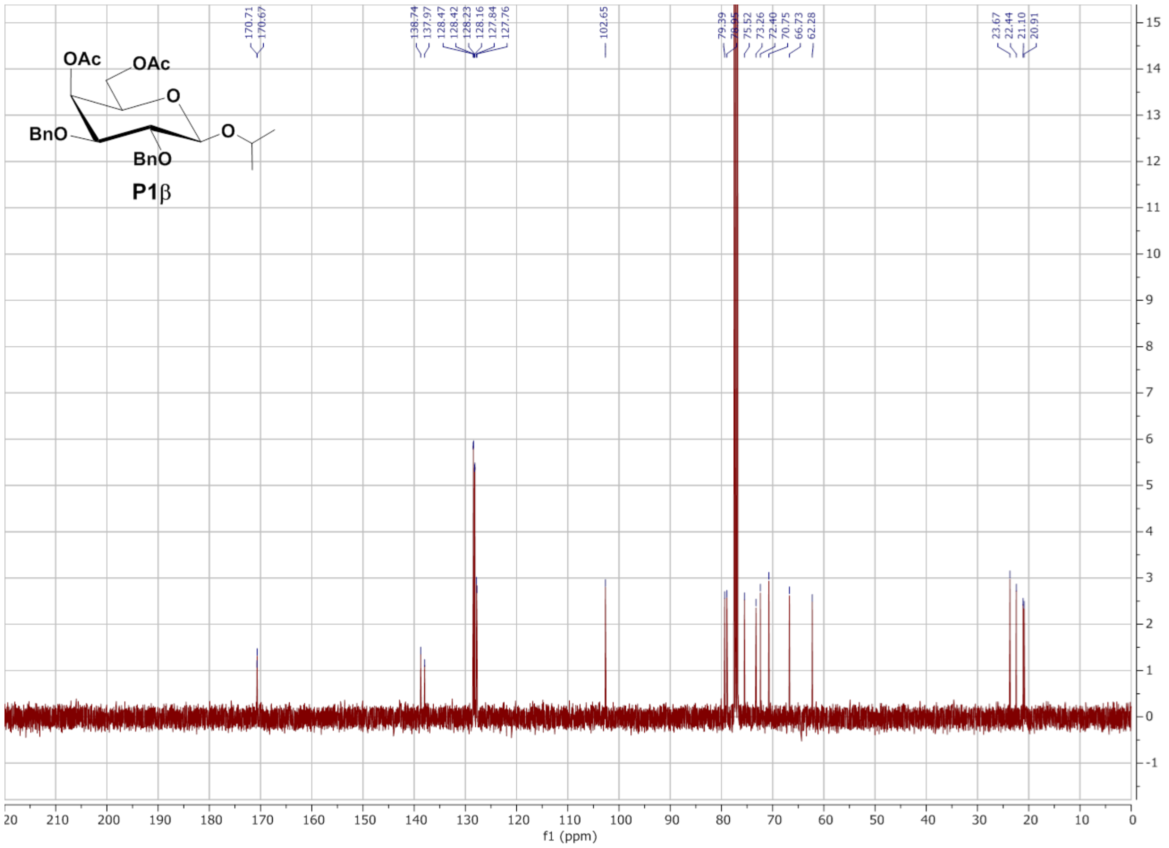

Figure S35: ^{13}C NMR spectrum of **P1β**.

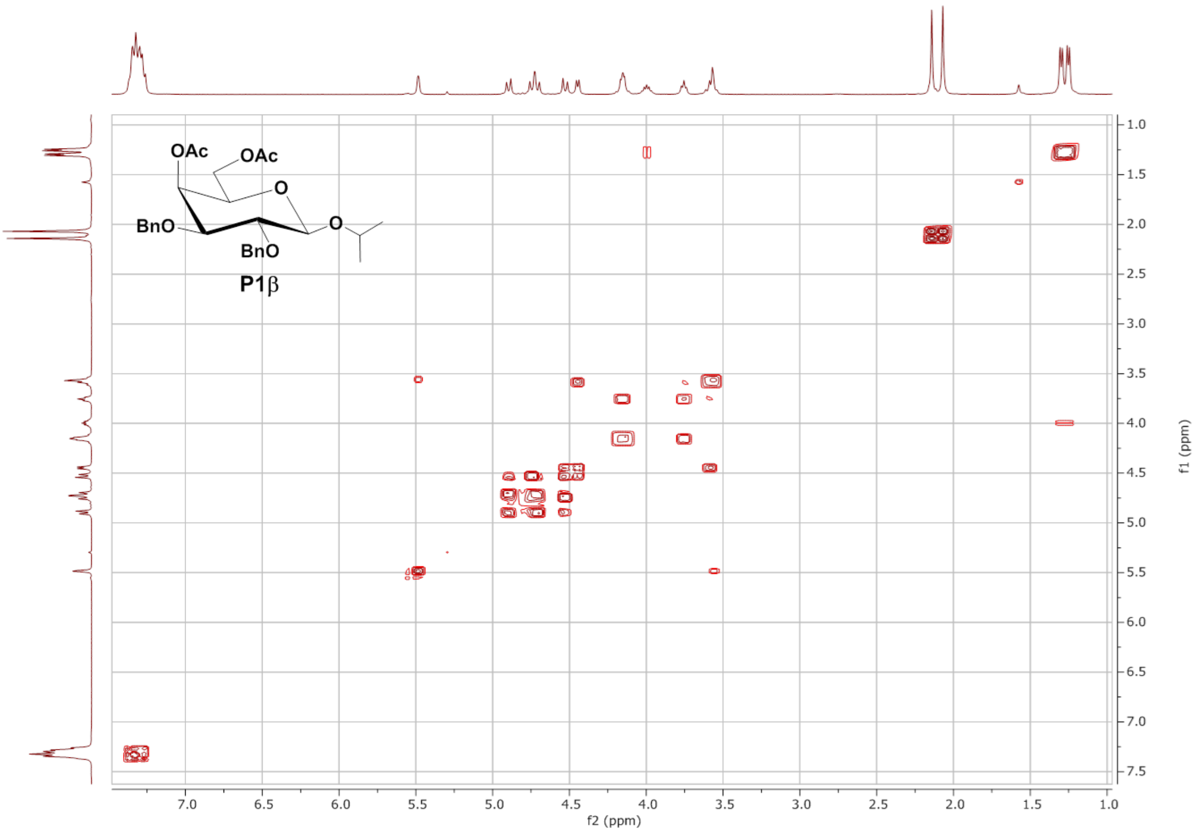

Figure S36: COSY NMR spectrum of **P1β**.

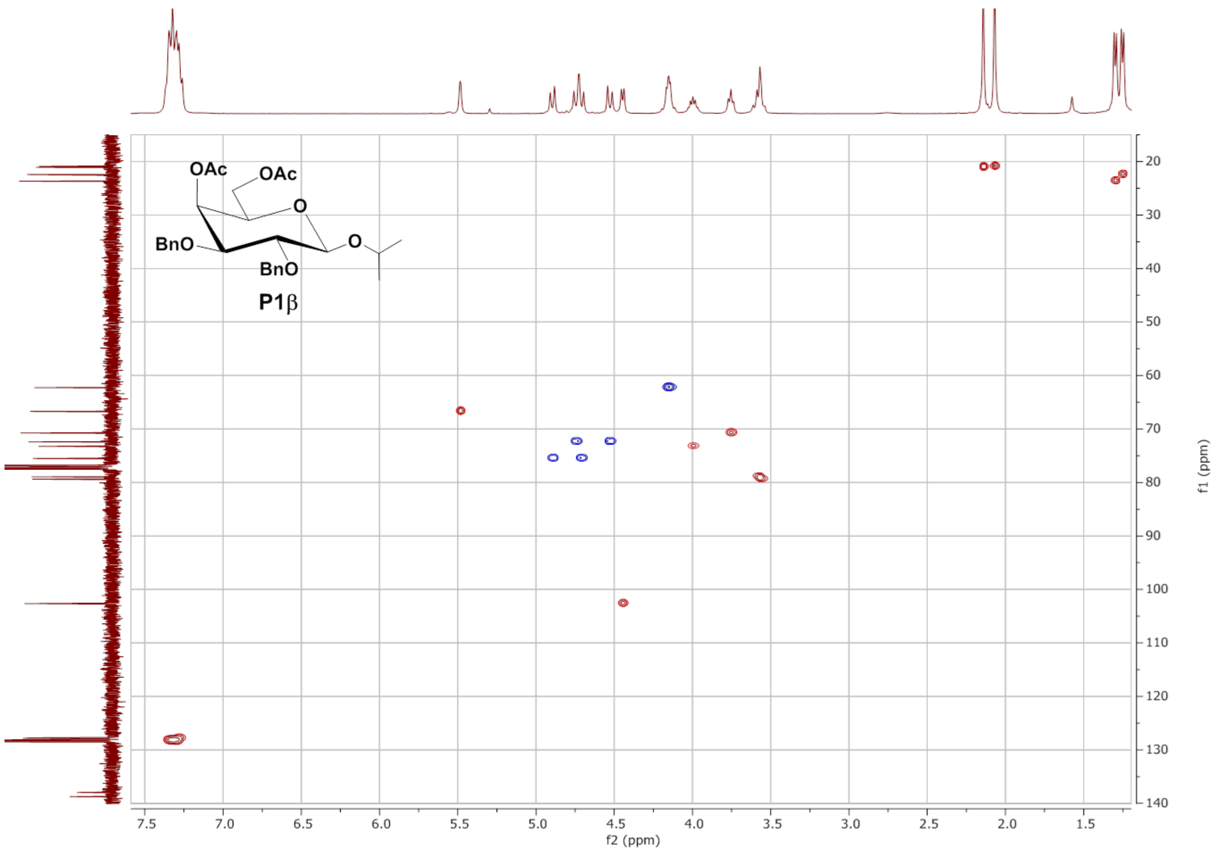

Figure S37: HSQC NMR spectrum of **P1β**.

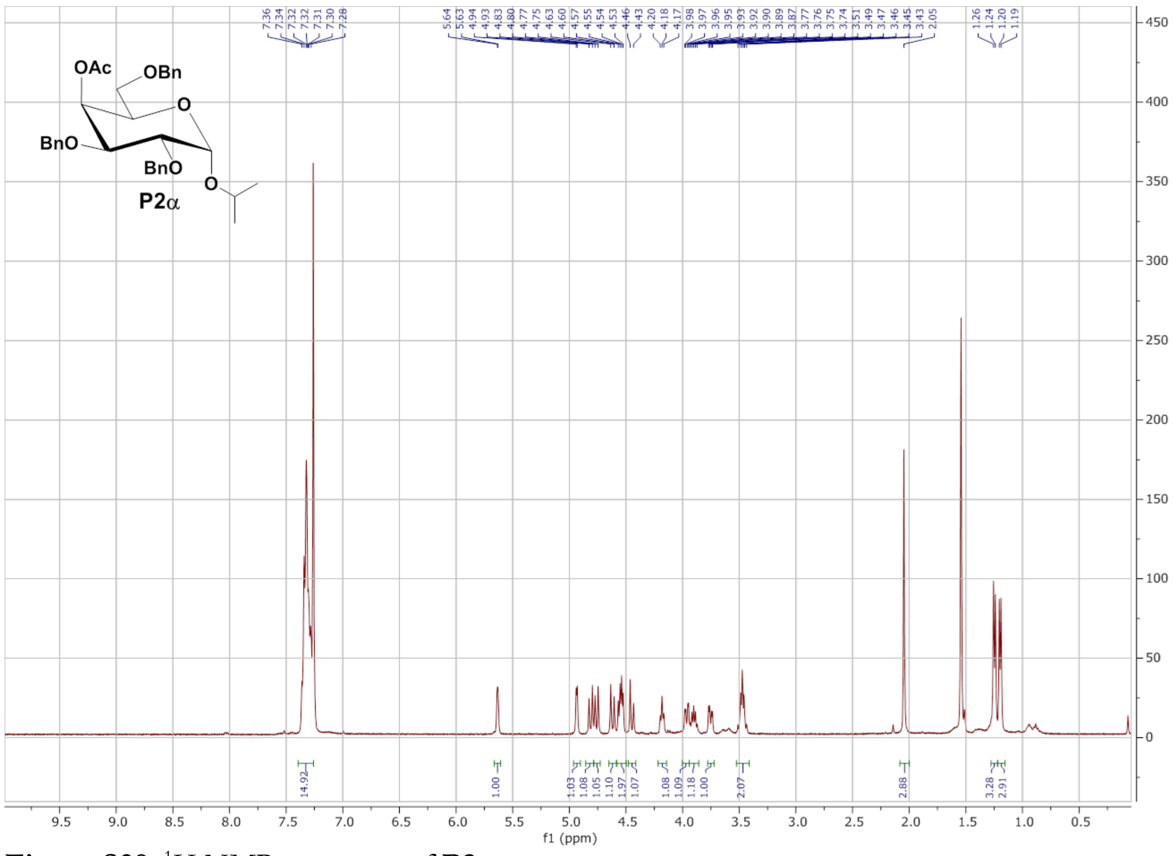

Figure S38: ^{1}H NMR spectrum of **P2α**.

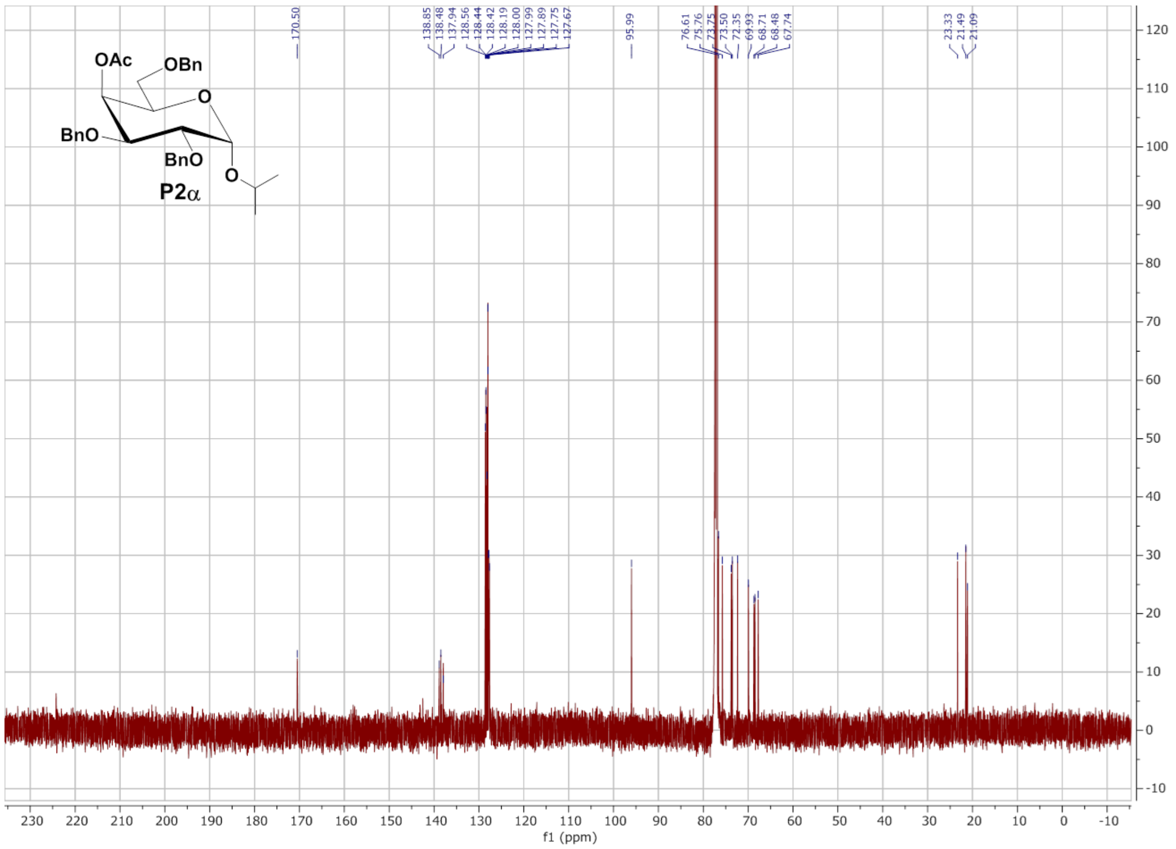

Figure S39: ^{13}C NMR spectrum of **P2α**.

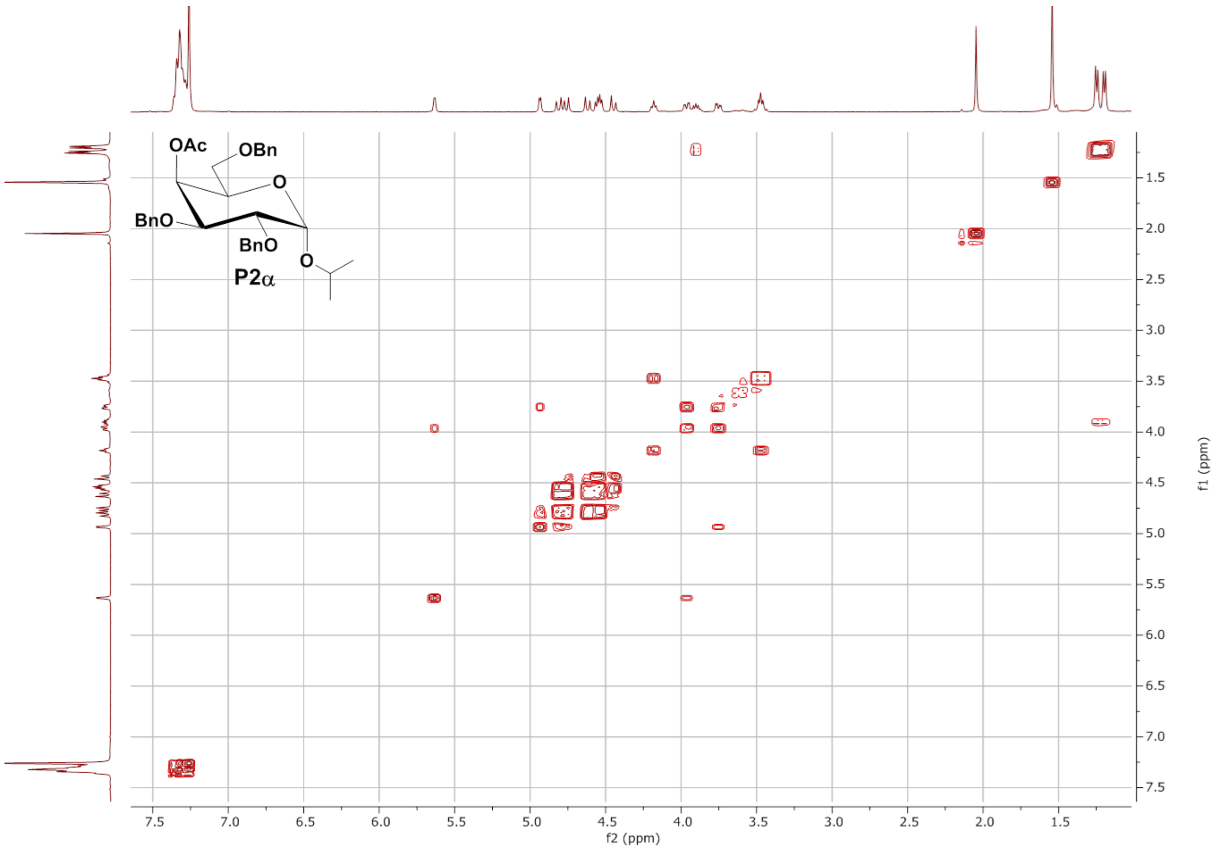

Figure S40: COSY NMR spectrum of **P2α**.

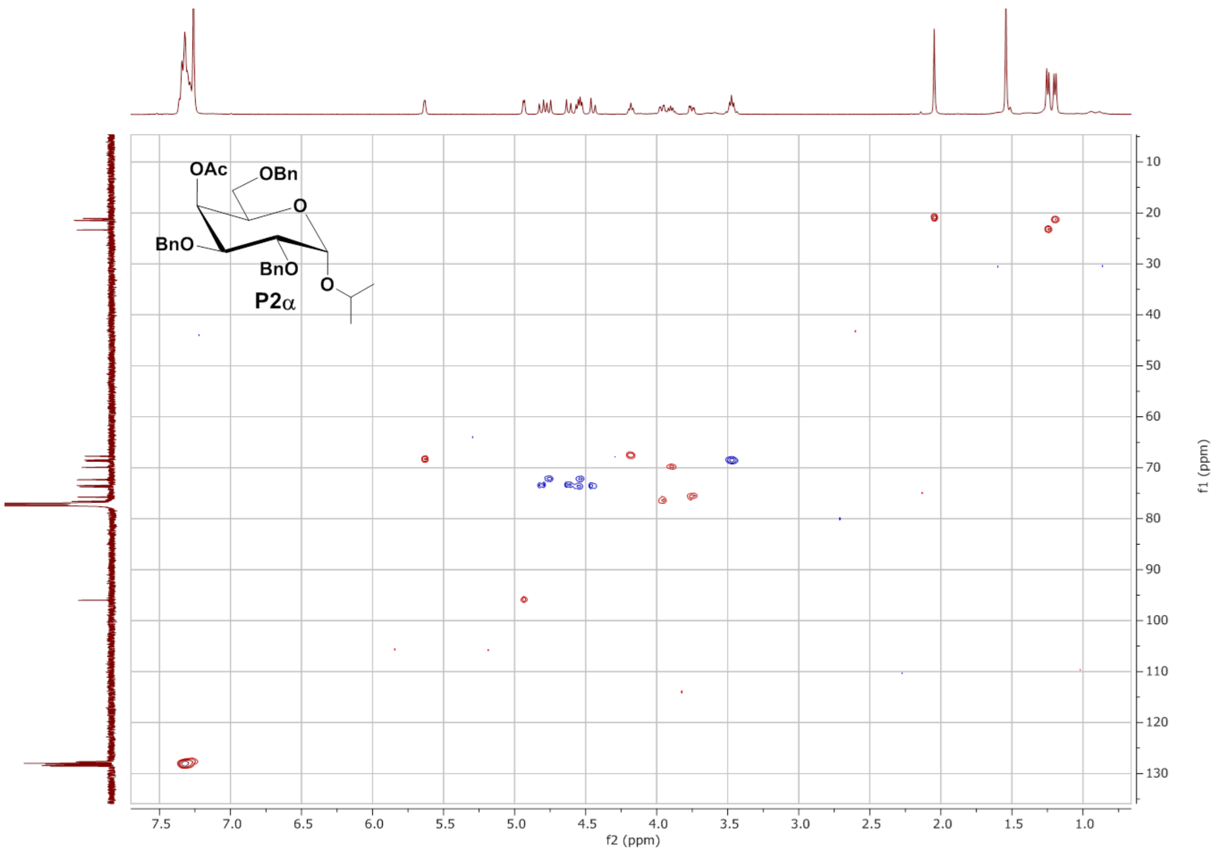

Figure S41: HSQC NMR spectrum of **P2α**.

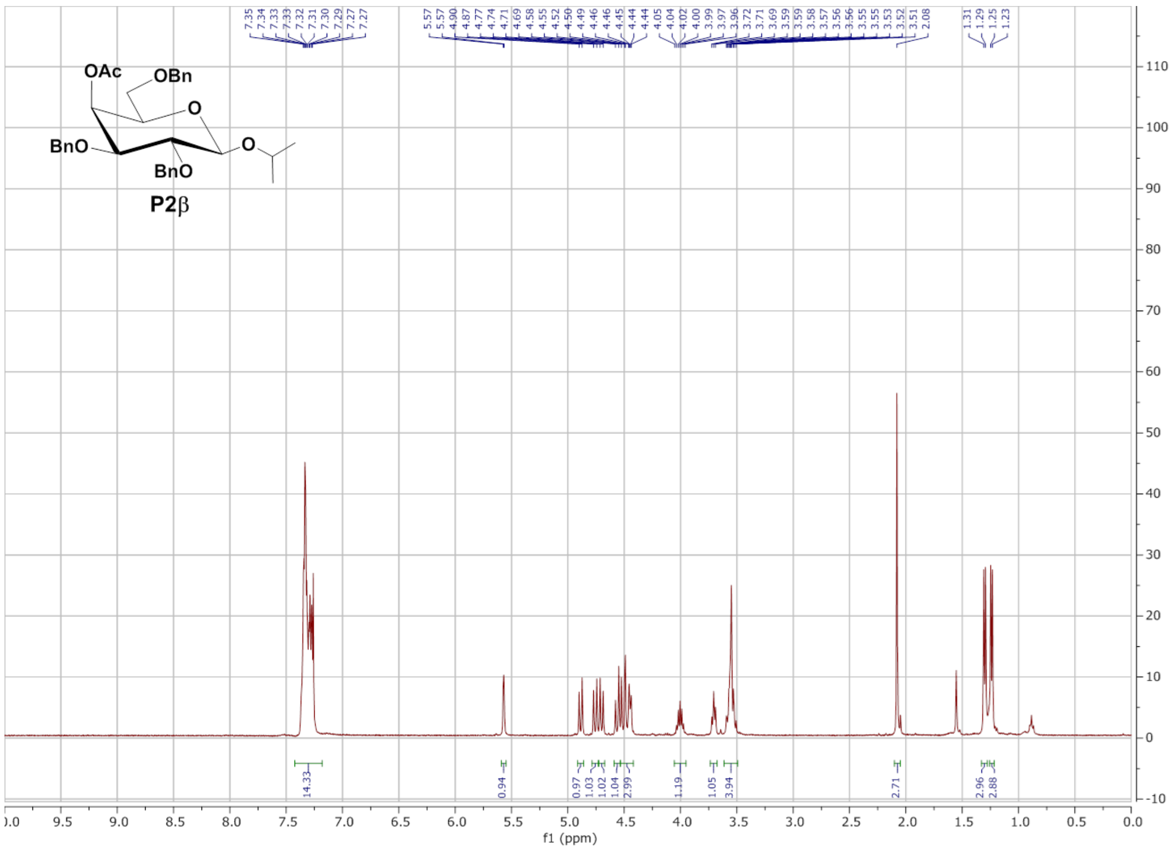

Figure S42: ^{1}H NMR spectrum of **P2 β**.

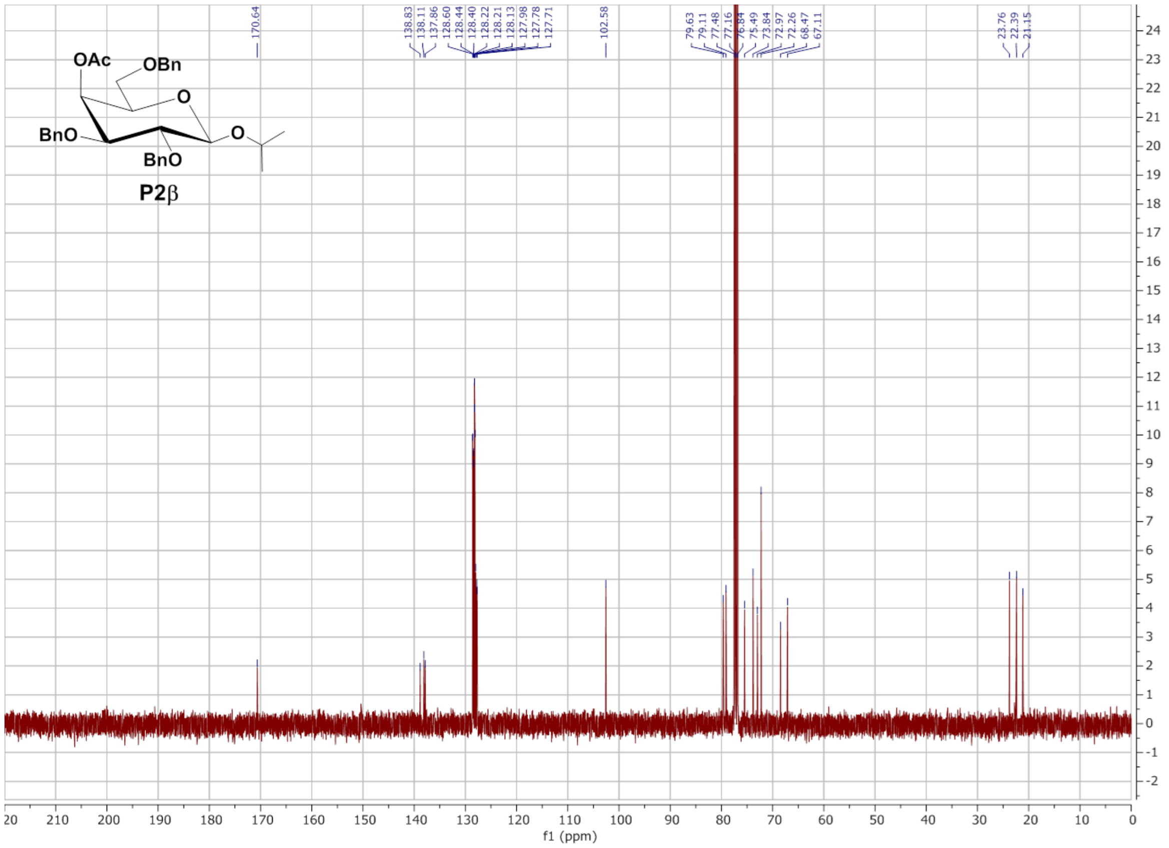

Figure S43: ^{13}C NMR spectrum of **P2β**.

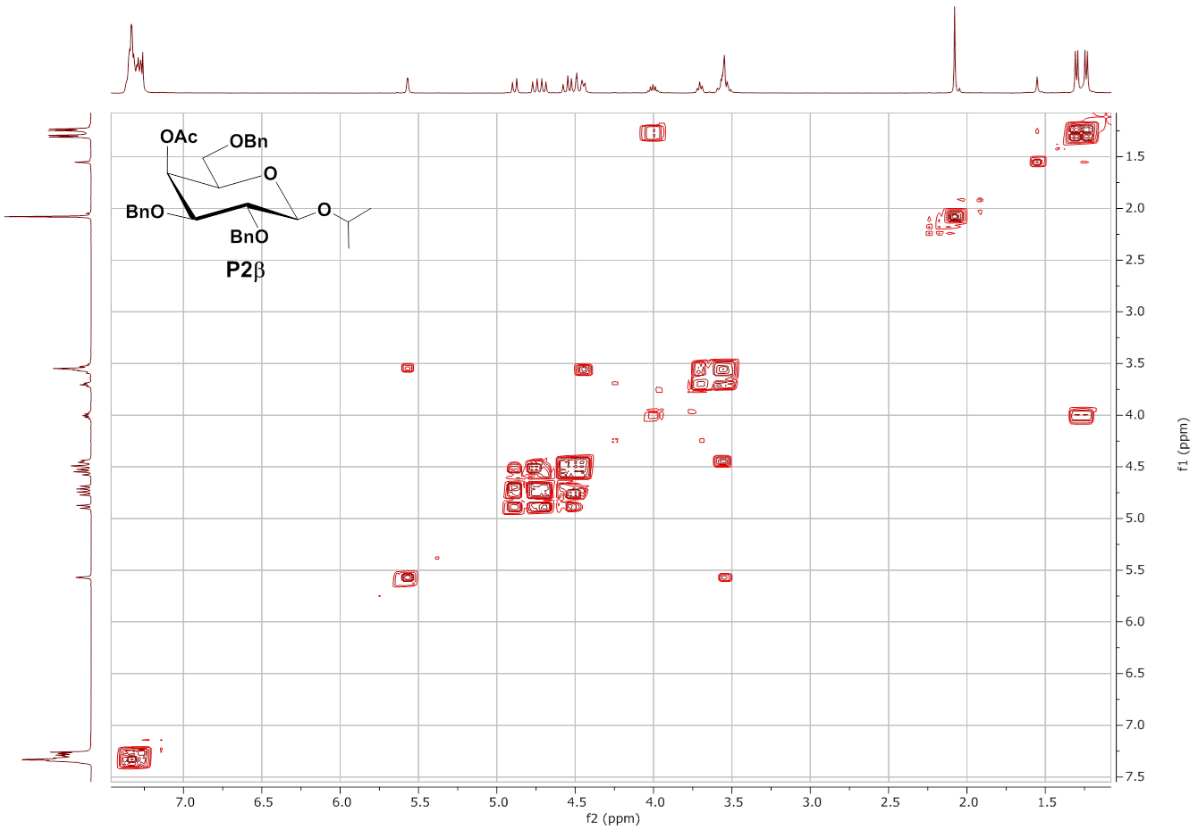

Figure S44: COSY NMR spectrum of **P2β**.

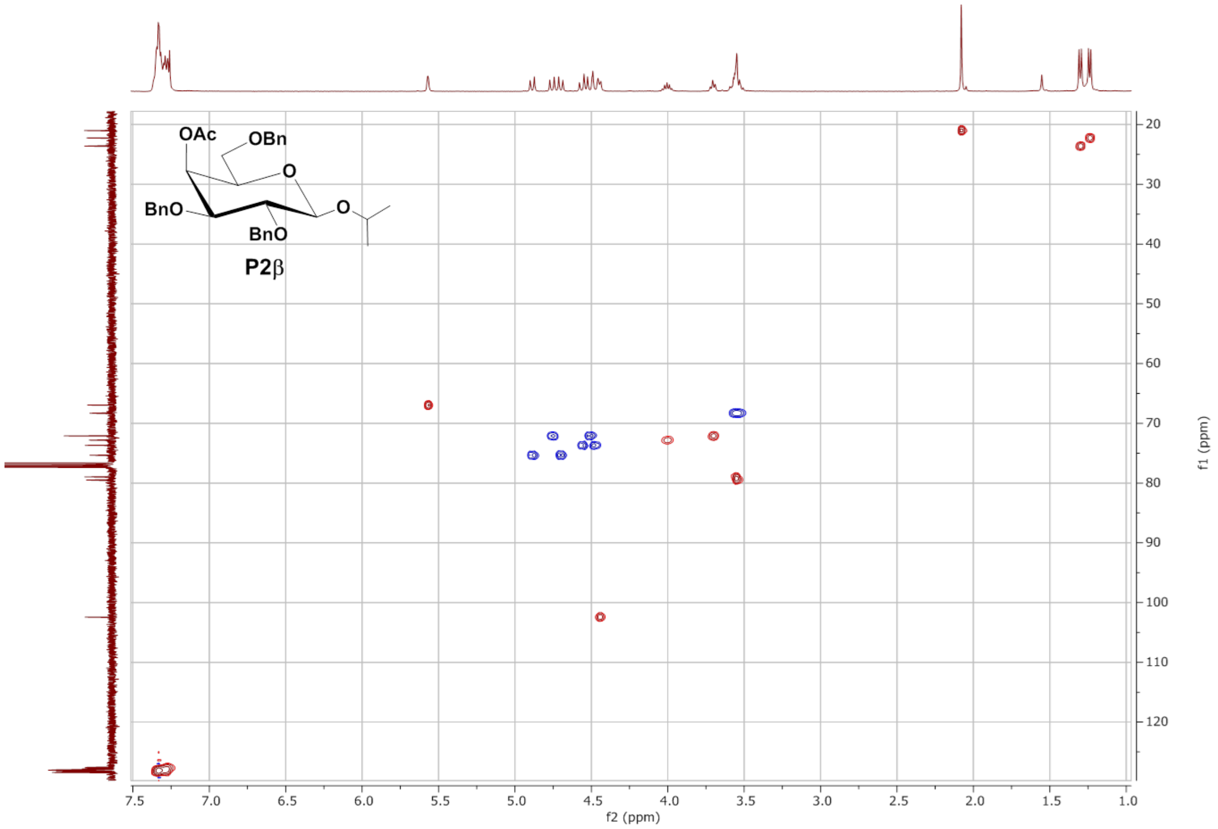

Figure S45: HSQC NMR spectrum of **P2β**.

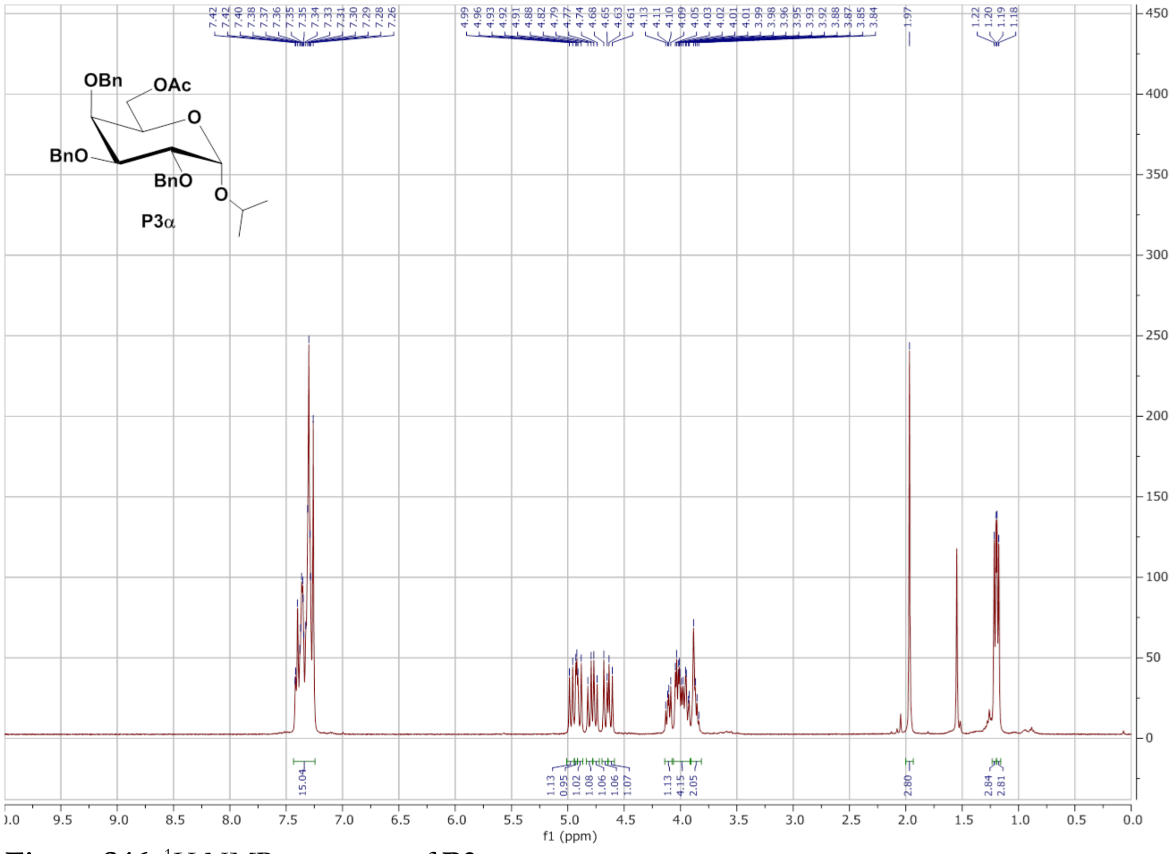

Figure S46: ^{1}H NMR spectrum of **P3α**.

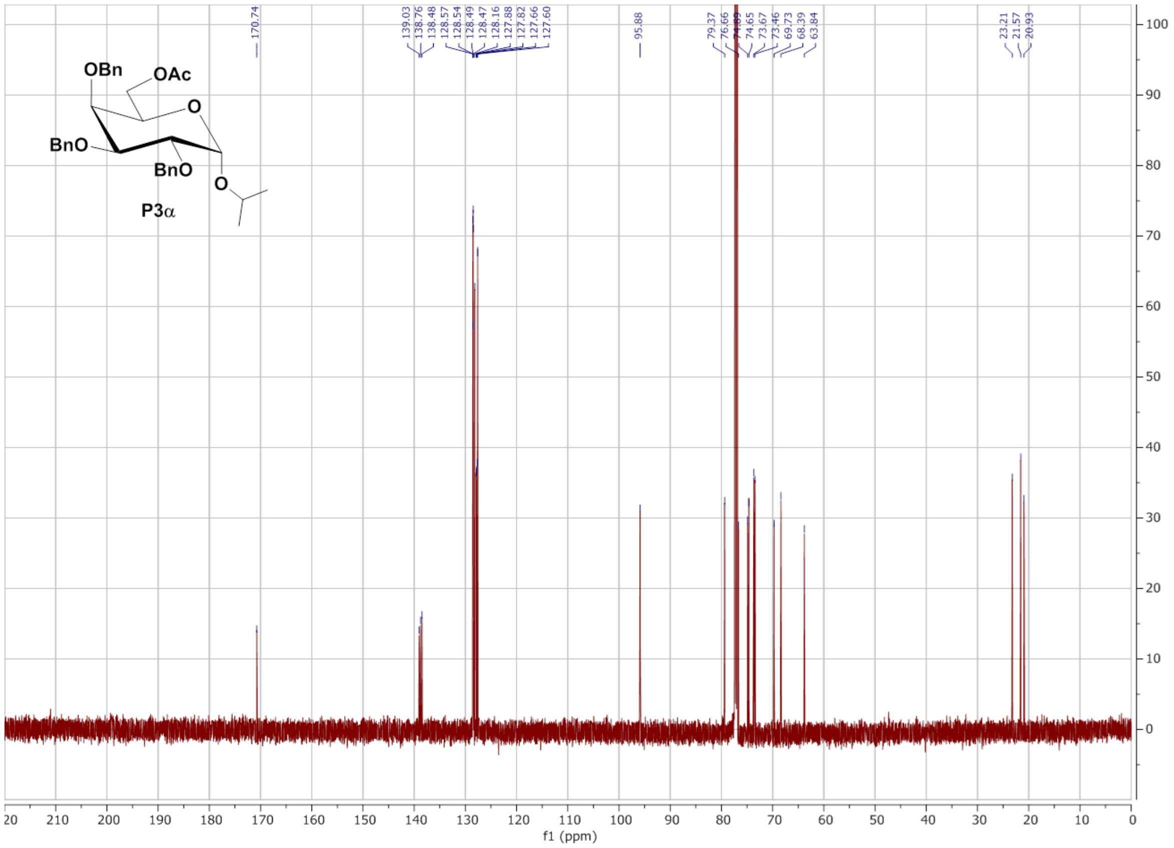

Figure S47: ^{13}C NMR spectrum of **P3α**.

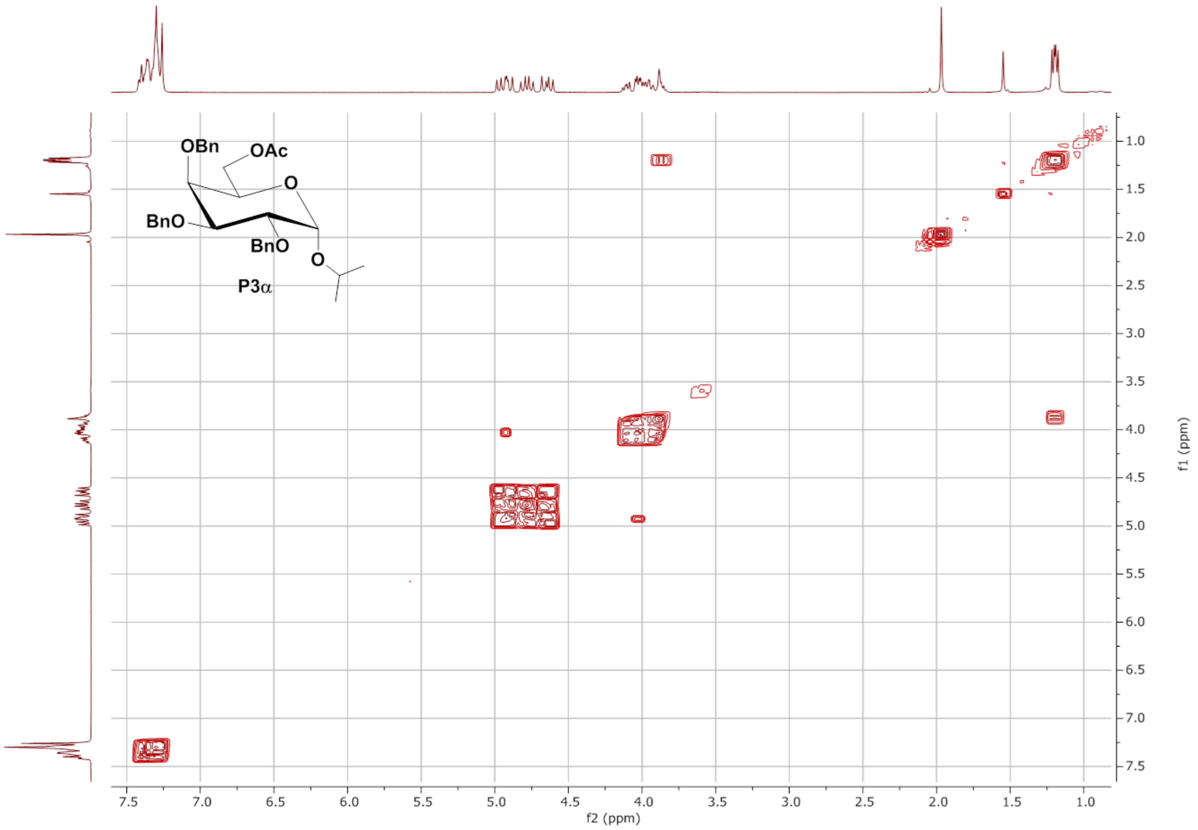

Figure S48: COSY NMR spectrum of **P3α**.

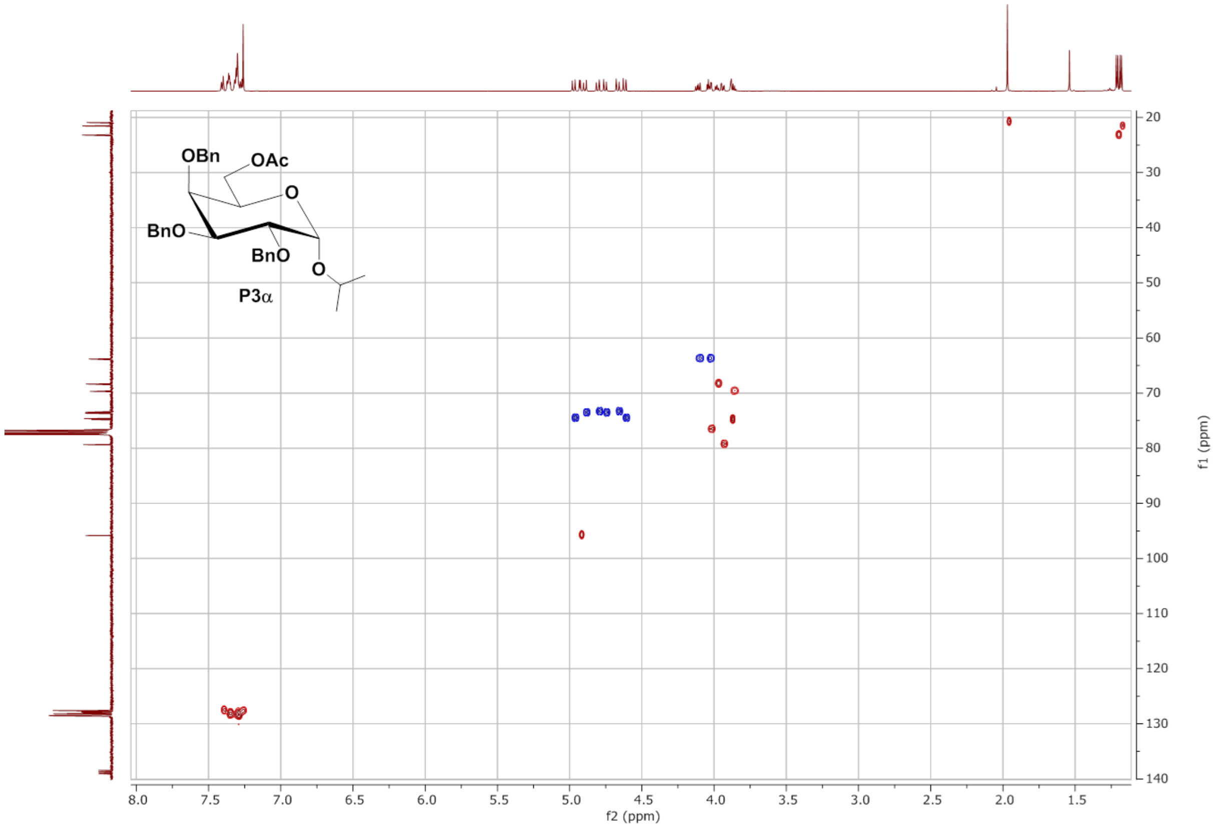

Figure S49: HSQC NMR spectrum of **P3α**.

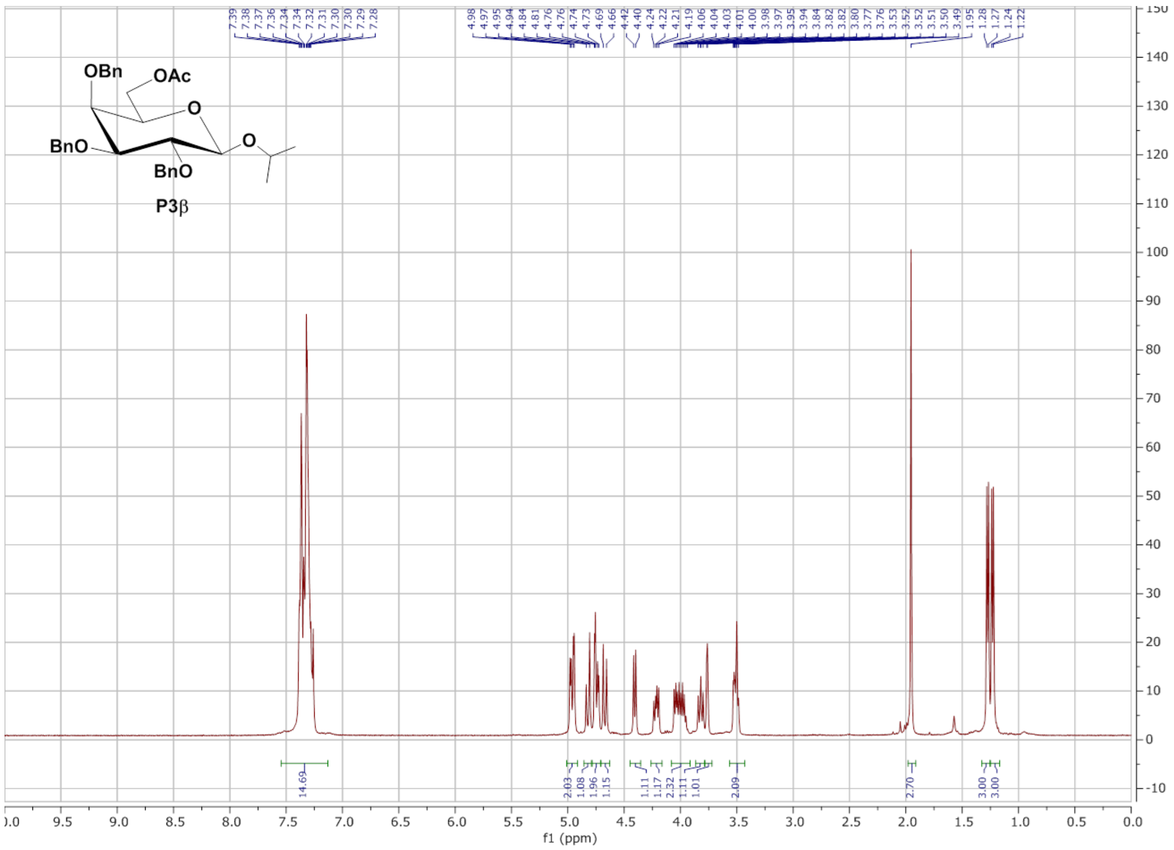

Figure S50: ^{1}H NMR spectrum of **P3β**.

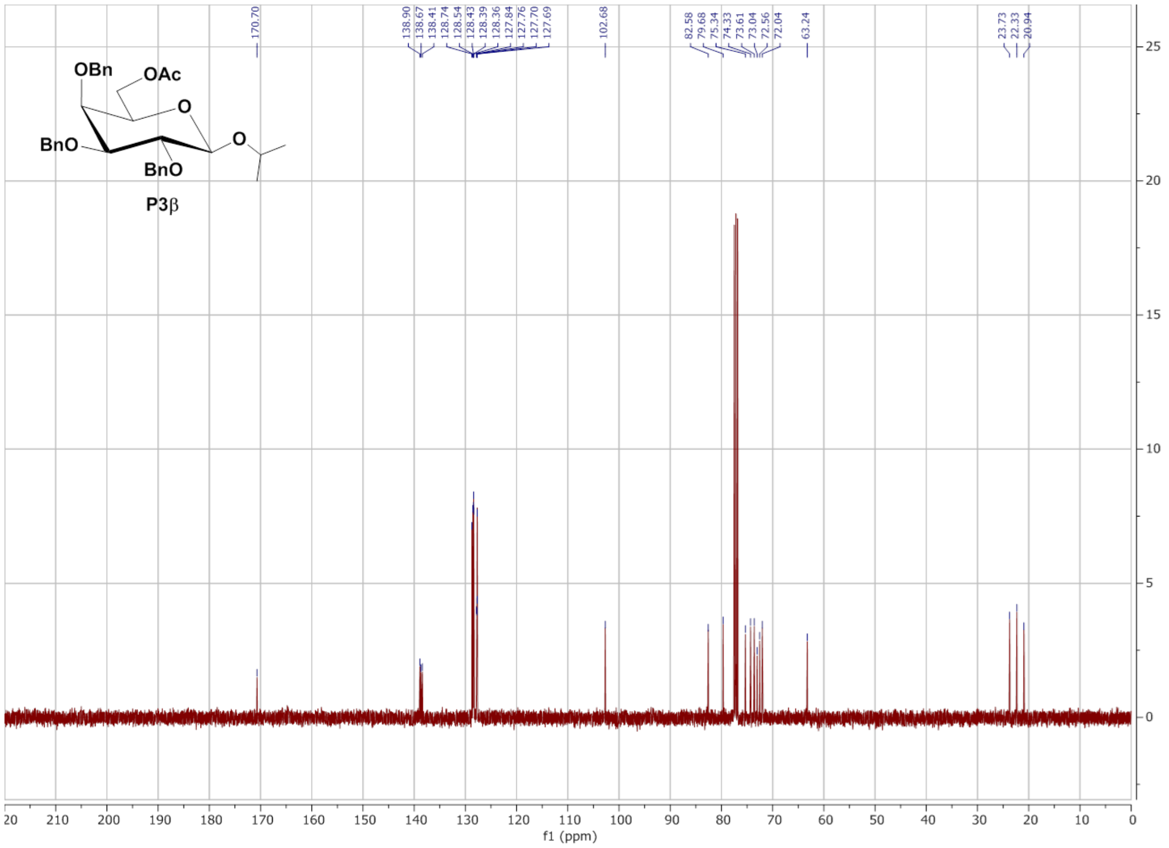

Figure S51: ^{13}C NMR spectrum of **P3β**.

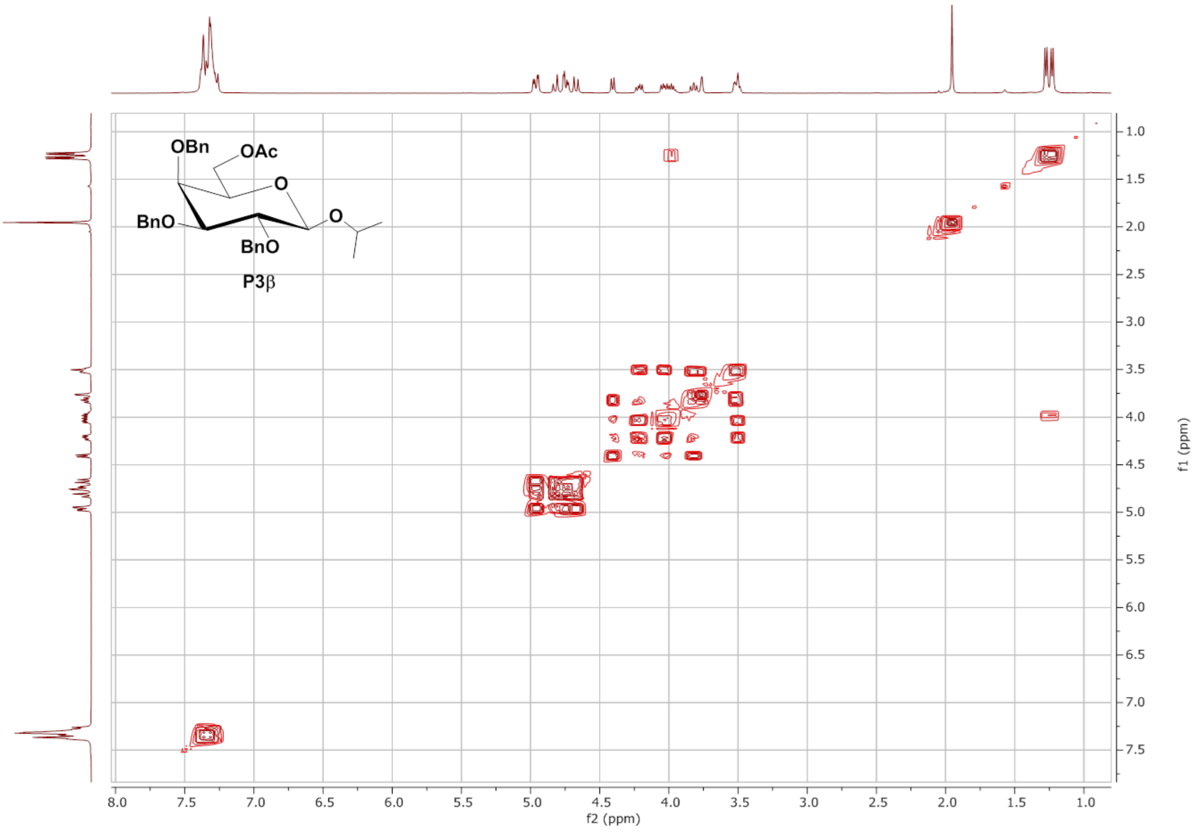

Figure S52: COSY NMR spectrum of **P3β**.

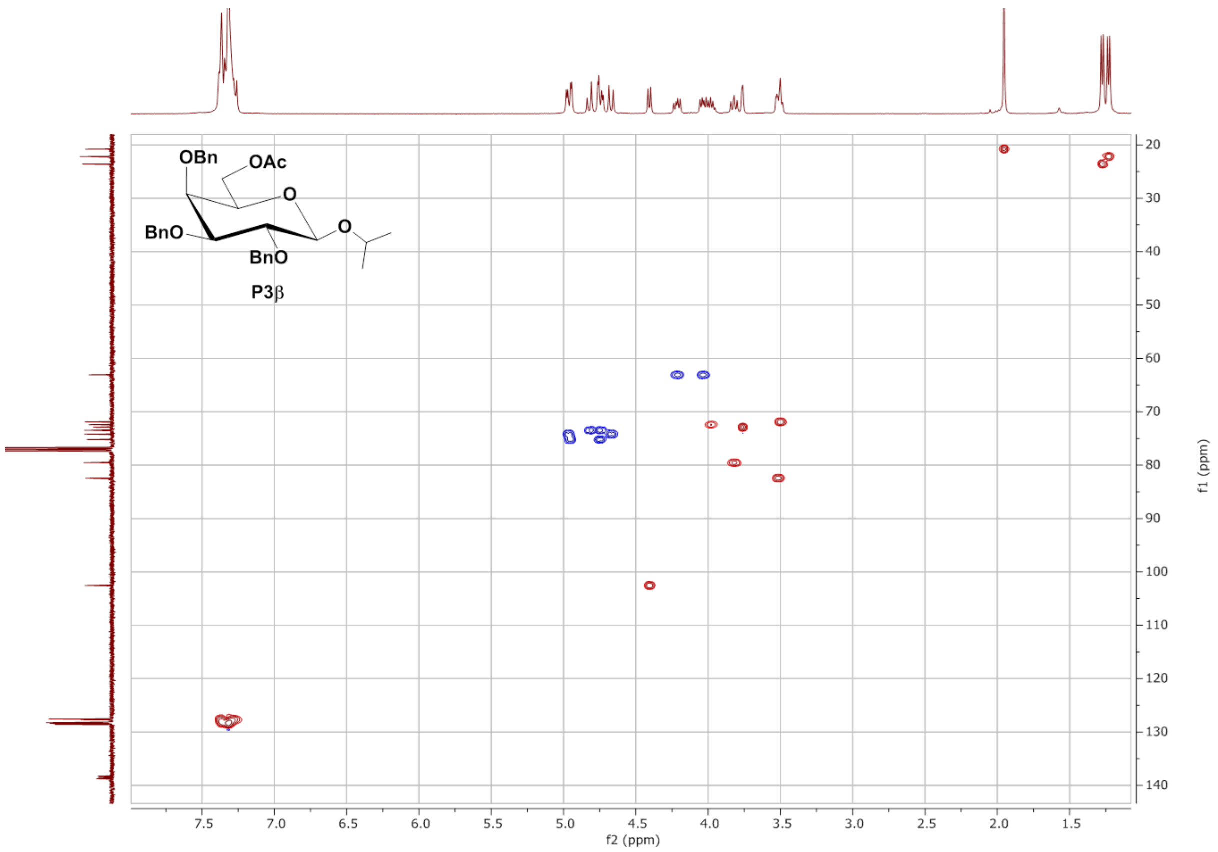

Figure S53: HSQC NMR spectrum of **P3β**.

Experimental Details

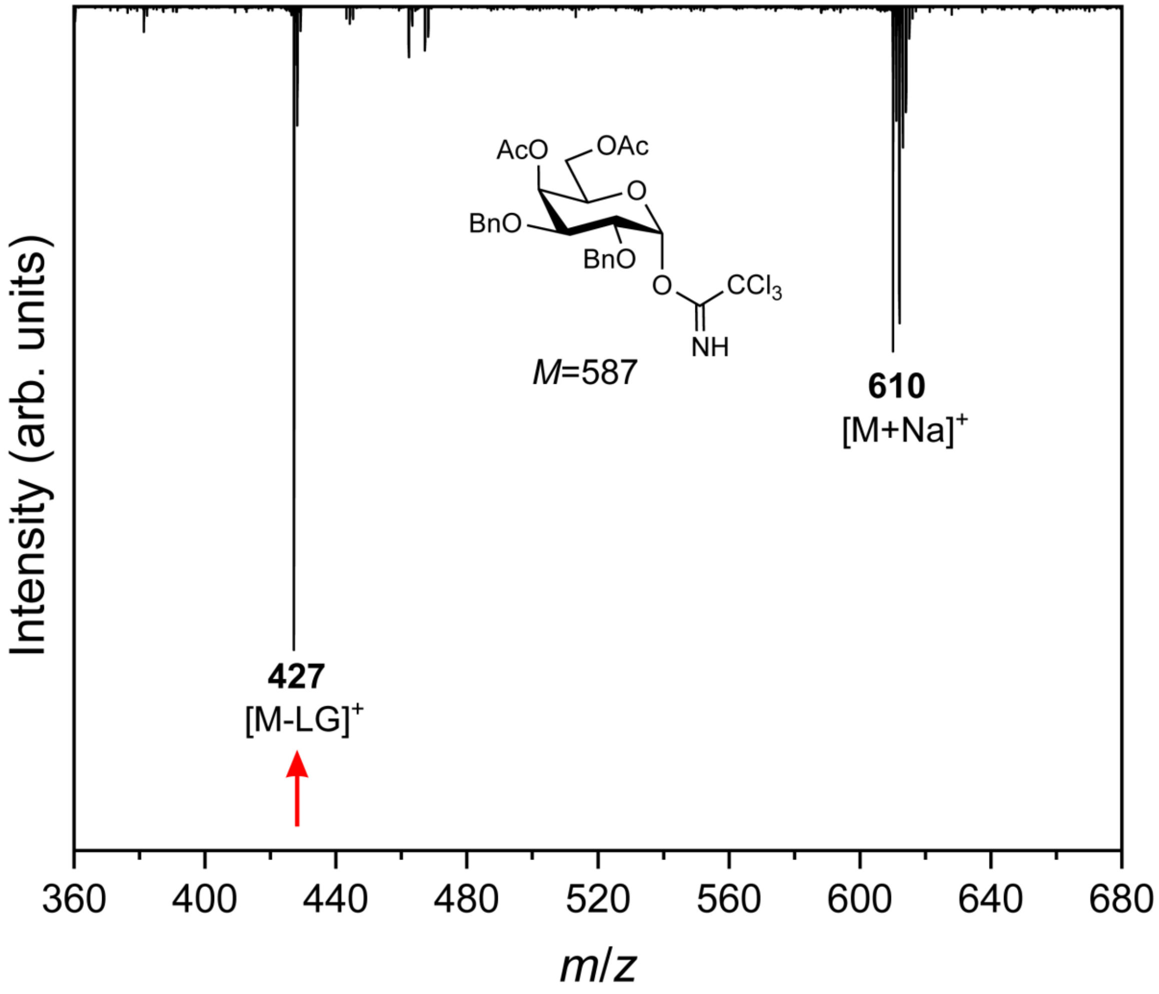

Figure S54. Exemplary mass spectrum of 2,3-di-benzyl-4,6-di-acetyl galactose (α imidate). Glycosyl cations (m/z = 427) were generated using in-source fragmentation of the respective precursor ions.

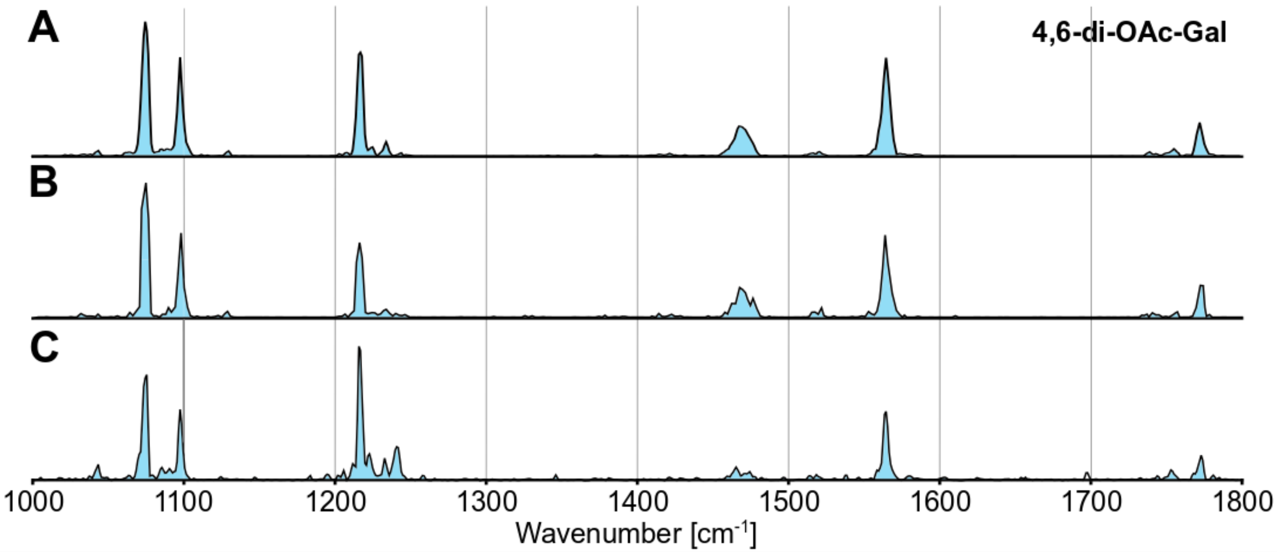

Figure S55. Comparison between experimental spectra of 2,3-di-benzyl-4,6-di-acetyl Galactose with α (A) and β imidate (B) and β thioether (C) as leaving group.

Theory

The initial screening of the conformational space of 2,3,4,6-tetra-O-benzyl-D-galactopyranose (**Bn**), 4-O-acetyl-2,3,6-tri-O-benzyl-D-galactopyranose (**4Ac**), 6-O-acetyl-2,3,4-tri-O-benzyl-D-galactopyranose (**6Ac**) and 4,6-di-O-acetyl-2,3-di-O-benzyl-D-galactopyranose (**46Ac**) cations has been performed with a FAFOOM genetic algorithm-based (GA) search tool interfaced to FHI-aims full electron numerical atomic orbitals code (version 171003). All available rotatable bonds and a ring pucker have been selected as degrees of freedom. We performed 10 individual GA runs for each investigated carbocation. The settings of each GA run are shown in Table 1. The local geometry optimizations were done using dispersion-corrected PBE+vdW^{TS} generalized gradient approximation density-functional theory (GGA-DFT) and in *light* basis set settings implemented in FHI-aims. Number of individual DFT optimizations are shown in table 2.

Supplementary Tab. 1 Parameters. GA parameters used in initial search.

	Parameter	Value
Molecule	Distance_cutoff_1	1.2
	Distance_cutoff_2	2.15
	Rmsd_cutoff_uniq	0.25
GA settings	Popsize	10
	Prob_for_crossing	0.95
	Prob_for_mut_pyranosering	0.6
	Prob_for_mut_torsion_	0.8
	Fitness_sum_limit	1.2
	Selection	Roulette wheel
	Max_mutations_torsion	3
	max_iter	30

Supplementary Tab. 2. Number of selected structures. Total number of structures at different steps.

Glycosyl cation	All generated structures	Unique structures	Selected oxo-carbenium-type	Selected dioxolenium- or oxonium type
**4,6OAc**	412	300	4	29
**4Ac**	685	378	9	10
**6Ac**	287	208	18	11
**Bn**	313	249	9	10

After completion of all GA runs, all structures generated for each carbocation were merged and clustered using root-mean square deviation (RMSD) metric for distances between heavy atoms. The tight RMSD=0.1 Å cutoff was selected to judge structural similarity. The two-fold symmetry of benzyl rings were included in similarity comparison. The RMSD calculations were performed in mdtraj-1.9.1 python module[6], while further hierarchical clustering was done with scipy-1.1.0 python module. Number of unique structures obtained for each cation is shown in Table 2. Next, we measured a distance between anomeric carbon C1 and the respective oxygen in the acetyl group and plotted the relative energy as a function of this distance (Fig 1).

All optimized structures seamlessly sort into three groups: the covalently bound dioxolenium-type cation (C=O—C1 distance below 2.0 Å), weakly interacting oxocarbenium-type cation (distance between 2.0 and 3.0 Å) and non-interaction oxocarbenium-type cation (C=O—C1 distance above 3.0 Å). Several lowest energy structures from dioxolenium and oxocarbenium-type structures were selected (number of selected conformers showed in Table 2) and further reoptimized at PBE0+D3/6-311+G(d,p) level of theory[23] with default convergence criteria in Gaussian09, RevD.01[7]. After each optimization, we extracted the C=O—C1 distance, assigned ring pucker to each structure and performed a frequency analysis within harmonic approximation. The presented IR spectra are normalized and scaled by 0.965 factor.

The potential energy of each molecule was finally computed using Resolution-of-Identity[8] MP2 method, extrapolated to the complete basis set. These calculations were performed using ORCA-4.1 software [9] The extrapolation was done using two-point extrapolation[10] with def2-TZVPP and def2-QZVPP basis sets and auxiliary def2-QZVPP/C basis set for RI. Our previous calculations on monosaccharides showed that RI yields virtually identical energies to those from canonical MP2 calculations. Grid5 settings and tight SCF convergence were requested to obtain full convergence. The reported conformational energies are corrected by free-energy contributions at 78K derived from harmonic vibrational calculations performed described in previous paragraph.

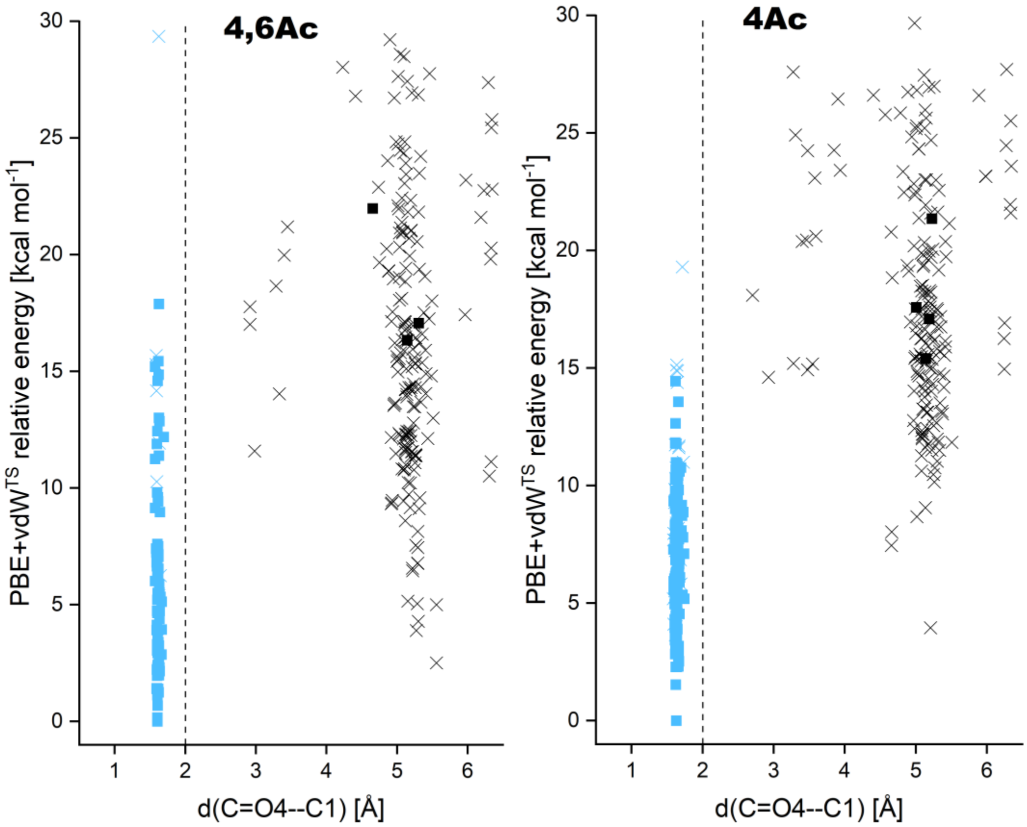

Figure S56. Energy hierarchys of the initial sampling for galactosyl cations generated from 4,6Ac (left) and 4Ac (right) precursor. The bold markers indicate structures bearing same ring pucker as the lowest energy structure.

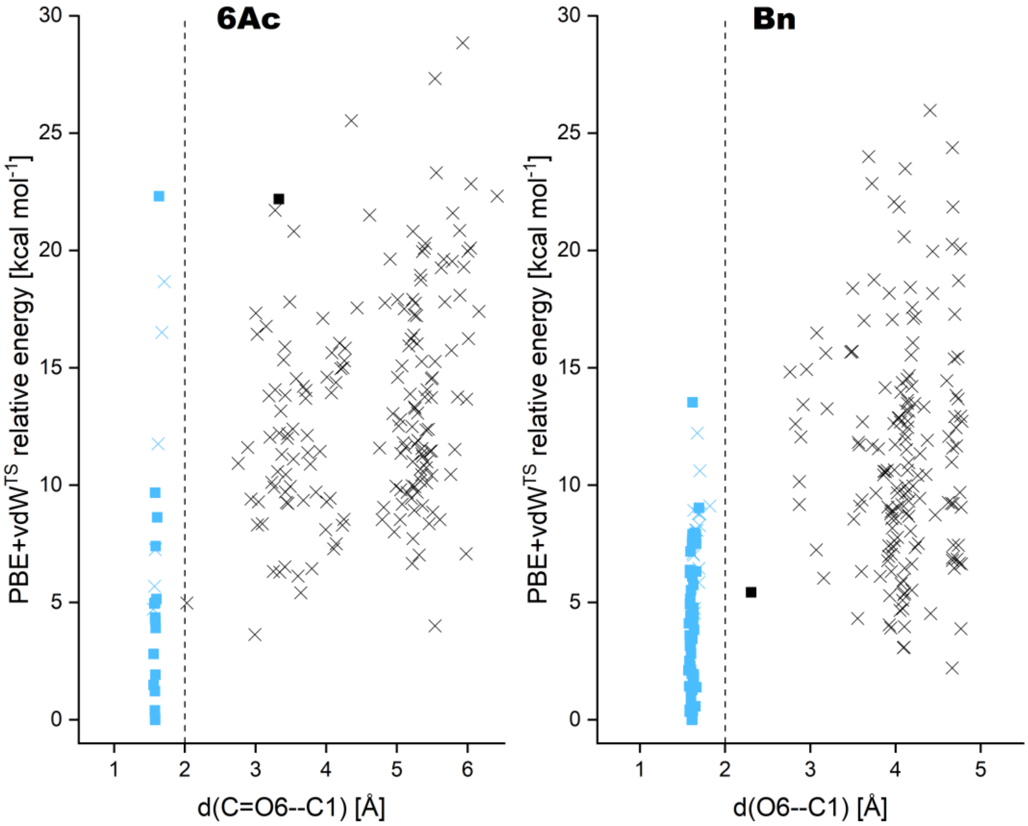

Figure S57. Energy hierarchies of the initial sampling for galactosyl cations generated from 6Ac (left) and Bn (right) precursor. The bold markers indicate structures bearing same ring pucker as the lowest energy structure.

Supplementary Tab. 3 List of conformations of **46Ac** reoptimized with PBE0+D3 functional: conformers' id labels, assigned ring puckers, distances between anomeric carbon and oxygen, energies computed at PBE0+D3/6-311+G(d,p) level of theory, harmonic free energy at 78K, RI-MP2 single-point energy extrapolated to the complete basis set from def2-TZVPP and def2-QZVPP basis sets and relative MP2/CBS energy corrected with harmonic free energy from DFT in kcal mol^{-1}. Conformers analysed in the paper are indicated with a label in parenthesis. The PBE0+D3/6-311+G(d,p) energies labelled with an asterisk (*) have been computed using Gaussian16, RevA.03[11] defaults.

id	Ring	d(O4—C1) [Å]	d(O6—C1) [Å]	E(PBE0+D3) [Ha]	F(78K) [Ha]	E(MP2/CBS(3,4)) [Ha]	$E_{MP2l}+F_{DFT}$ [kcal/mol]
o4/conf_0000	1S5	1.52	5.29	-1455.90250076	-1455.430780	-1455.11498488	0.00
**(A)** o4/conf_0001	1S5	1.52	5.29	-1455.90250061	-1455.430775	-1455.11498736	0.00
o4/conf_0002	1S5	1.53	5.27	-1455.90158401	-1455.430074	-1455.11397537	0.50
o4/conf_0003	1S5	1.52	5.29	-1455.90035750	-1455.429075	-1455.11232751	1.39
o4/conf_0004	1S5	1.53	5.19	-1455.90102763	-1455.429282	-1455.11275599	1.41
o4/conf_0005	1S5	1.52	3.58	-1455.90182656	-1455.429575	-1455.11325129	1.42
o4/conf_0006	1S5	1.53	5.19	-1455.90102756	-1455.429276	-1455.11275746	1.42
o4/conf_0007	1S5	1.52	3.58	-1455.90182660	-1455.429583	-1455.11325096	1.42
o4/conf_0008	1S5	1.52	3.58	-1455.90182662	-1455.429578	-1455.11325024	1.42
o4/conf_0012	1S5	1.54	5.12	-1455.89944175	-1455.428394	-1455.11140658	1.82
o4/conf_0016	1S5	1.54	5.12	-1455.89944185	-1455.428395	-1455.11140645	1.82
o4/conf_0010	1S5	1.53	3.58	-1455.90066409	-1455.428754	-1455.11191371	2.05
o4/conf_0011	1S5	1.52	5.25	-1455.89949999	-1455.428124	-1455.11101329	2.28
o4/conf_0014	1S5	1.52	3.98	-1455.89998257	-1455.428103	-1455.11151437	2.28
o4/conf_0015	1S5	1.52	3.98	-1455.89998257	-1455.428100	-1455.11151441	2.28
o4/conf_0017	1S5	1.52	3.98	-1455.89998269	-1455.428104	-1455.11151591	2.28
o4/conf_0013	1S5	1.52	5.32	-1455.89937829	-1455.428016	-1455.11067361	2.48
o4/conf_0009	1S5	1.52	5.37	-1455.89867062	-1455.427670	-1455.11018787	2.56
o4/conf_0018	1S5	1.53	5.29	-1455.89845809	-1455.427133	-1455.11008114	2.83
o4/conf_0019	1S5	1.53	5.29	-1455.89845806	-1455.427133	-1455.11008177	2.83
**(B)**o6/conf_0001	1C4	5.24	1.50	-1455.89692178	-1455.425808	-1455.10667221	4.84
o6/conf_0000	1C4	5.21	1.50	-1455.89677001	-1455.425271	-1455.10655561	5.15
o6/conf_0002	1C4	5.23	1.52	-1455.89563510	-1455.423738	-1455.10502042	6.36
o6/conf_0003	1C4	5.10	1.52	-1455.89466631	-1455.422787	-1455.10435725	6.77
o6/conf_0007	1C4	5.22	1.51	-1455.89240240	-1455.421225	-1455.10225765	7.65
o6/conf_0006	1C4	5.22	1.51	-1455.89240244	-1455.421170	-1455.10225695	7.68
o6/conf_0005	1C4	5.11	1.50	-1455.89234094	-1455.420775	-1455.10236049	7.82
o6/conf_0008	1C4	5.22	1.51	-1455.89252813	-1455.421058	-1455.10181685	8.11
o6/conf_0009	1C4	5.22	1.51	-1455.89042355	-1455.419434	-1455.09953190	9.24
open/conf_0000*	4E	5.18	4.95	-1455.90874340	-1455.455611	-1455.09624411	9.23
**(C)**open/conf_0001*	3E	5.05	3.57	-1455.90759157	-1455.453774	-1455.09587973	9.89
open/conf_0002*	4H3	5.24	3.97	-1455.90578899	-1455.453046	-1455.09270194	11.21
open/conf_0003*	5H4	4.93	2.80	-1455.90684990	-1455.453013	-1455.09313436	11.62
o4/conf_0000*	1S5	1.52	5.29	-1455.92610970	-1455.468942	-1455.11498488	0.00

Tab. 4 List of conformations of **4Ac** reoptimized with PBE0+D3 functional: conformers' id labels, assigned ring puckers, distances between anomeric carbon and oxygen, energies computed at PBE0+D3/6-311+G(d,p) level of theory, harmonic free energy at 78K, RI-MP2 single-point energy extrapolated to the complete basis set from def2-TZVPP and def2-QZVPP basis sets and relative MP2/CBS energy corrected with harmonic free energy from DFT in kcal mol^{-1}. Conformers analysed in the paper are indicated with a label in parenthesis.

id	Ring	d(O4—C1) [Å]	E(PBE0+D3) [Ha]	F(78K) [Ha]	E(MP2/CBS(3,4)) [Ha]	$E_{MP2l}+F_{DFT}$ [kcal/mol]
closed/conf_0000	1S5	1.54	-1573.47152557	-1572.928058	-1572.55198718	0.00
closed/conf_0003	1S5	1.54	-1573.47152595	-1572.928059	-1572.55198892	0.00
**(D)**closed/conf_0001	1S5	1.53	-1573.47100865	-1572.927341	-1572.55101773	0.74
closed/conf_0008	1S5	1.54	-1573.46880105	-1572.925616	-1572.54905841	1.66
closed/conf_0005	1S5	1.55	-1573.46872542	-1572.925303	-1572.54918355	1.73
closed/conf_0002	1S5	1.53	-1573.46890537	-1572.925660	-1572.54884936	1.83

**(D')**closed/conf_0006	1S5	1.53	-1573.46803198	-1572.925425	-1572.54799371	1.97
closed/conf_0007	1S5	1.53	-1573.46794329	-1572.924809	-1572.54849298	1.98
closed/conf_0009	1S5	1.55	-1573.46826954	-1572.925200	-1572.54819533	2.13
closed/conf_0004	1S5	1.53	-1573.46810878	-1572.925787	-1572.54685184	2.50
**(E)**open/conf_0001	1C4	4.60	-1573.46161308	-1572.918588	-1572.54540097	3.86
open/conf_0002	1C4	4.61	-1573.46117228	-1572.918000	-1572.54419725	4.70
open/conf_0003	1C4	5.04	-1573.45984535	-1572.918236	-1572.54235782	4.88
open/conf_0004	1C4	5.20	-1573.45709629	-1572.915323	-1572.54082813	5.94
open/conf_0005	1C4	5.19	-1573.45708092	-1572.914385	-1572.54016288	6.94
open/conf_0000	1C4	5.16	-1573.46330046	-1572.921808	-1572.53866988	7.12
open/conf_0006	1C4	5.18	-1573.45389717	-1572.911640	-1572.53602956	9.26
**(F)**open/conf_0007	4E	5.15	-1573.45013008	-1572.909881	-1572.52972590	11.95
open/conf_0009	4H3	5.24	-1573.44864103	-1572.908104	-1572.52777324	13.36

Supplementary Tab. 5 List of conformations of **6Ac** reoptimized with PBE0+D3 functional: conformers' id labels, assigned ring puckers, distances between anomeric carbon and oxygen, energies computed at PBE0+D3/6-311+G(d,p) level of theory, harmonic free energy at 78K, RI-MP2 single-point energy extrapolated to the complete basis set from def2-TZVPP and def2-QZVPP basis sets and relative MP2/CBS energy corrected with harmonic free energy from DFT in kcal mol^{-1}. Conformers analysed in the paper are indicated with a label in parenthesis.

id	Ring	d(O6—C1) [Å]	E(PBE0+D3) [Ha]	F(78K) [Ha]	E(MP2/CBS(3,4)) [Ha]	$E_{MP2l}+F_{DFT}$ [kcal/mol]
**(G)**closed/conf_0002	1C4	1.50	-1573.46697000	-1572.923204	-1572.54588247	0.00
closed/conf_0001	1C4	1.51	-1573.46673808	-1572.923699	-1572.54423621	0.58
open/conf_0002	1C4	1.51	-1573.46710953	-1572.922886	-1572.54523730	0.69
closed/conf_0003	1C4	1.50	-1573.46649874	-1572.923142	-1572.54427233	0.75
closed/conf_0000	1C4	1.51	-1573.46666794	-1572.922779	-1572.54479703	0.76
closed/conf_0004	1C4	1.50	-1573.46541919	-1572.921192	-1572.54435259	1.25
closed/conf_0005	1C4	1.51	-1573.46501048	-1572.921582	-1572.54260832	1.84
closed/conf_0006	1C4	1.50	-1573.46397366	-1572.920676	-1572.54203452	2.12
**(H)**open/conf_0012	1,4B	3.99	-1573.45444847	-1572.912296	-1572.53828010	3.76
closed/conf_0009	1C4	1.51	-1573.46137495	-1572.918704	-1572.53867005	3.84
closed/conf_0007	1C4	1.51	-1573.46126866	-1572.917903	-1572.53935165	3.85
closed/conf_0008	1C4	1.51	-1573.46050496	-1572.917456	-1572.53845015	4.21
**(I)**open/conf_0000	5S1	2.82	-1573.45739139	-1572.915071	-1572.53691353	4.72
open/conf_0001	4H3	5.40	-1573.45560014	-1572.914401	-1572.53525742	5.06
open/conf_0003	3E	3.53	-1573.45270547	-1572.911898	-1572.53241137	6.60
open/conf_0005	3E	3.50	-1573.45275014	-1572.912086	-1572.53215861	6.67
open/conf_0006	5H4	2.90	-1573.45166305	-1572.910598	-1572.53010670	8.20
open/conf_0009	4E	5.11	-1573.45056553	-1572.909919	-1572.52955850	8.29
open/conf_0007	3E	3.69	-1573.45057261	-1572.909339	-1572.52990838	8.43
open/conf_0008	3H4	3.18	-1573.45042155	-1572.909906	-1572.52829603	9.00
open/conf_0014	4E	5.12	-1573.44887469	-1572.908912	-1572.52696682	9.48
open/conf_0004	3E	3.52	-1573.45023216	-1572.909058	-1572.52802296	9.58
open/conf_0011	3H4	5.94	-1573.44799493	-1572.907770	-1572.52606392	10.21
open/conf_0017	4E	3.89	-1573.44724788	-1572.907668	-1572.52523941	10.33
open/conf_0018	5S1	2.86	-1573.44754870	-1572.906369	-1572.52669315	10.42
open/conf_0010	E3	5.21	-1573.44731905	-1572.907385	-1572.52464547	10.92
open/conf_0019	3E	5.28	-1573.44527274	-1572.905109	-1572.52470164	11.03
open/conf_0015	5H4	4.70	-1573.44755993	-1572.906616	-1572.52534251	11.12
open/conf_0016	4H5	3.89	-1573.44524319	-1572.903665	-1572.52424856	12.20

Supplementary Tab. 6 List of conformations of **Bn** reoptimized with PBE0+D3 functional: conformers' id labels, assigned ring puckers, distances between anomeric carbon and oxygen, energies computed at PBE0+D3/6-311+G(d,p) level of theory, harmonic free energy at 78K, RI-MP2 single-point energy extrapolated to the complete basis set from def2-TZVPP and def2-QZVPP basis sets and relative MP2/CBS energy corrected with harmonic free energy from DFT in kcal mol^{-1}. Conformers analysed in the paper are indicated with a label in parenthesis.

id	Ring	d(O6—C1) [Å]	E(PBE0+D3) [Ha]	F(78K) [Ha]	E(MP2/CBS(3,4)) [Ha]	$E_{MP2l}+F_{DFT}$ [kcal/mol]
**(J)**o6/conf_0004	1C4	1.53	-1691.03209838	-1690.416952	-1689.98351567	0.00
o6/conf_0007	1C4	1.53	-1691.03209836	-1690.416949	-1689.98351554	0.00
o6/conf_0002	1C4	1.52	-1691.03189435	-1690.417331	-1689.98286468	0.04
o6/conf_0009	1C4	1.53	-1691.03123341	-1690.415731	-1689.98366221	0.13
o6/conf_0001	1C4	1.53	-1691.03194198	-1690.416826	-1689.98324136	0.15
o6/conf_0000	1C4	1.52	-1691.03270731	-1690.417405	-1689.98317426	0.31
o6/conf_0006	1C4	1.52	-1691.03227938	-1690.416413	-1689.98297242	0.79
o6/conf_0003	1C4	1.52	-1691.03227945	-1690.416408	-1689.98297235	0.80
o6/conf_0008	1C4	1.54	-1691.03057152	-1690.416216	-1689.98144407	0.80
open/conf_0009	B0,3	1.55	-1691.02503757	-1690.410803	-1689.97614198	4.05
**(K)**open/conf_0003	4E	4.71	-1691.02537192	-1690.412055	-1689.97430628	4.63
open/conf_0000	4H3	4.61	-1691.02396679	-1690.410990	-1689.97327687	5.06
open/conf_0001	4H3	4.01	-1691.02185630	-1690.409426	-1689.97041644	6.52
open/conf_0002	4H3	3.94	-1691.02024749	-1690.408090	-1689.96755051	8.14
open/conf_0007	5H4	3.51	-1691.01850723	-1690.405373	-1689.96819309	8.35
open/conf_0005	4H3	3.98	-1691.01829419	-1690.406365	-1689.96525575	9.44
open/conf_0008	4E	4.13	-1691.01654970	-1690.404186	-1689.96476184	10.02
open/conf_0004	3H4	3.87	-1691.01749369	-1690.404094	-1689.96517795	10.41
open/conf_0006	E4	3.89	-1691.01722149	-1690.404107	-1689.96313963	11.51

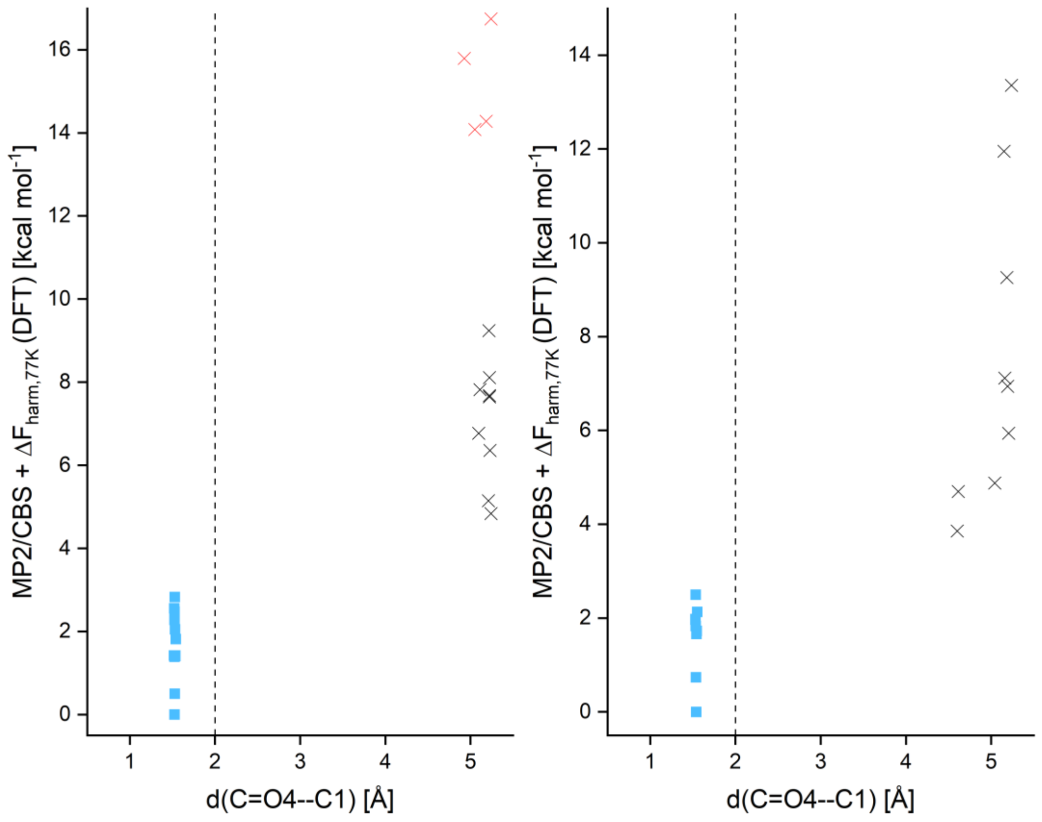

Figure S58. Energy hierarchies of the reoptimized structures for galactosyl cations generated from **4,6Ac** (left) and **4Ac** (right) precursor. The bold markers indicate structures bearing same ring pucker as the lowest energy structure. In case of the **4,6Ac** cation, black crosses indicate dioxolenium ions with C6-participation, whereas red crosses indicate oxonium structures with two unbound acetyl groups.

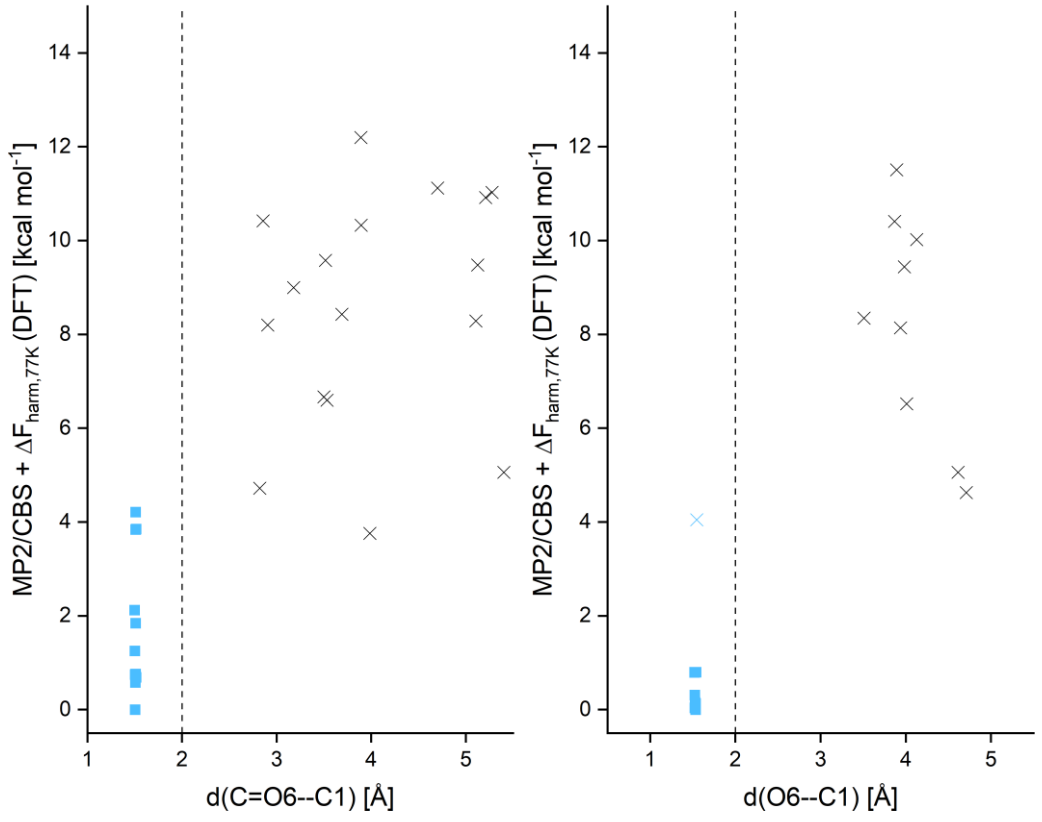

Figure S59. Energy hierarchies of the reoptimized structures for galactosyl cations generated from **6Ac** (left) and **Bn** (right) precursor. The bold markers indicate structures bearing same ring pucker as the lowest energy structure.

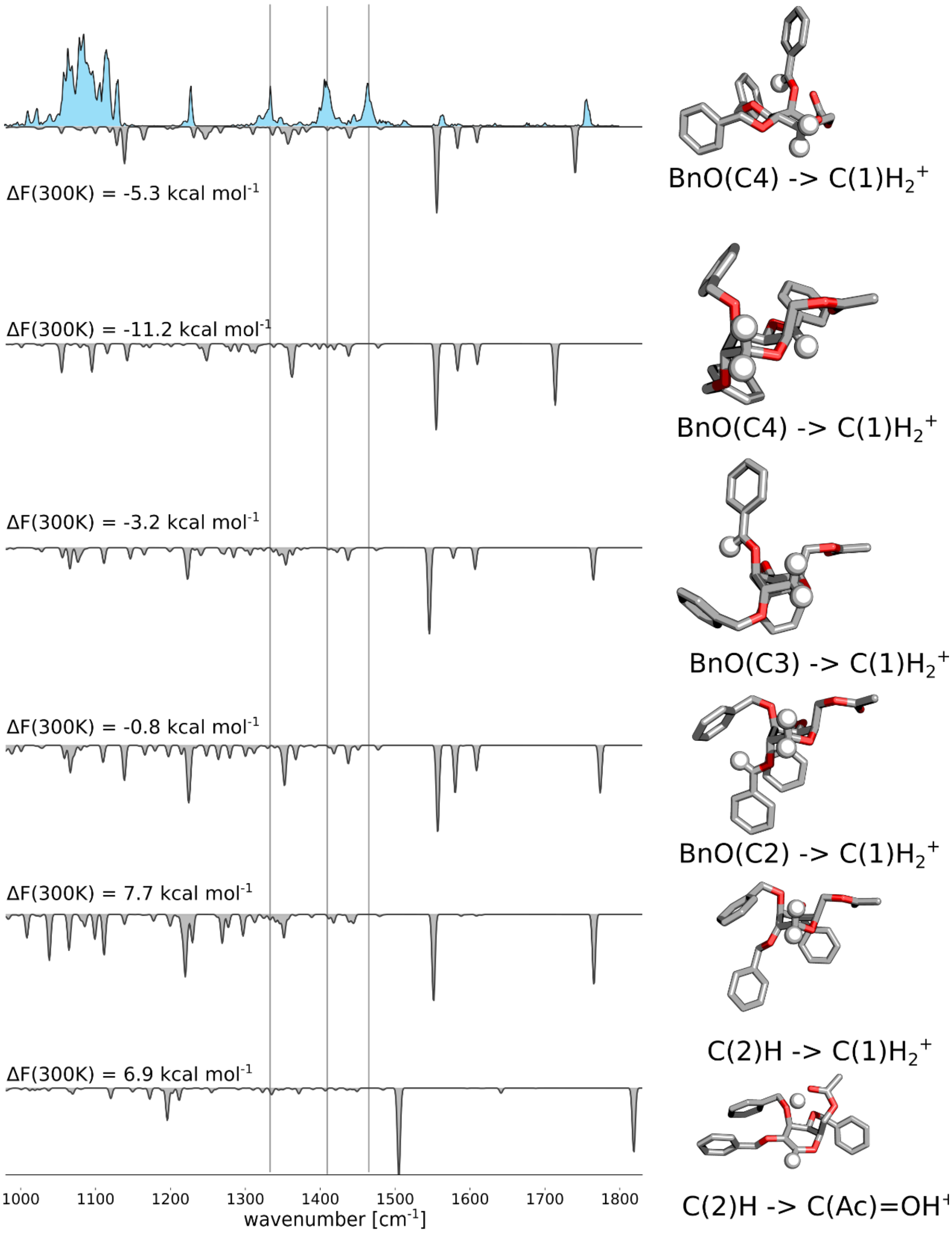

Figure S60. Different tautomers of **Ac6.** The numbers indicate relative free energy at 300K with respect to the conformer **G**. The structures are shown in the right, hydrogens at the anomeric carbon and at the carbon the they are transferred to are sown with white balls. The labels underneath represent from where and where to they were transferred. C2H means the carbon 2 in the pyranose ring, the BnO(CX) means a methylene carbon in the respective benzyl group. Although some of the structures have lower energy, none of them explains three bands at 1300 cm^{-1} and 1600 cm^{-1}.

References

1. Hahm, H. S.; Hurevich, M.; Seeberger, P. H. Automated assembly of oligosaccharides containing multiple cis-glycosidic linkages. *Nat. Commun.* **2016**, *7*, 12482.
2. Aly, M. R.; Rochaix, P.; Amessou, M.; Johannes, L.; Florent, J. C. Synthesis of globo- and isoglobotriosides bearing a cinnamoylphenyl tag as novel electrophilic thiol-specific carbohydrate reagents. *Carbohydr. Res.* **2006**, *341*, 2026.
3. Baek, J. Y.; Kwon, H. W.; Myung, S. J.; Park, J. J.; Kim, M. Y.; Rathwell, D. C.; Jeon, H. B.; Seeberger, P. H.; Kim, K. S. Directing effect by remote electron-withdrawing protecting groups at O-3 or O-4 position of donors in glucosylations and galactosylations. *Tetrahedron* **2015**, *71*, 5315.
4. Lourenço, E. C.; Ventura, M. R. The synthesis of compatible solute analogues—solvent effects on selective glycosylation. *Carbohydr. Res.* **2011**, *346*, 163.
5. Chatterjee, S.; Moon, S.; Hentschel, F.; Gilmore, K.; Seeberger, P. H. An Empirical Understanding of the Glycosylation Reaction. *J. Am. Chem. Soc.* **2018**, *140*, 11942.
6. Robert T. McGibbon, Kyle A. Beauchamp, Matthew P. Harrigan, C. Klein, Jason M. Swails, Carlos X. Hernández, Christian R. Schwantes, L.-P. Wang, Thomas J. Lane, Vijay S. Pande, MDTraj: A Modern Open Library for the Analysis of Molecular Dynamics Trajectories. *Biophys. J.* **2015**, 109, 1528-1532.
7. Gaussian 09, Revision D.01, M. J. Frisch, G. W. Trucks, H. B. Schlegel, G. E. Scuseria, M. A. Robb, J. R. Cheeseman, G. Scalmani, V. Barone, G. A. Petersson, H. Nakatsuji, X. Li, M. Caricato, A. Marenich, J. Bloino, B. G. Janesko, R. Gomperts, B. Mennucci, H. P. Hratchian, J. V. Ortiz, A. F. Izmaylov, J. L. Sonnenberg, D. Williams-Young, F. Ding, F. Lipparini, F. Egidi, J. Goings, B. Peng, A. Petrone, T. Henderson, D. Ranasinghe, V. G. Zakrzewski, J. Gao, N. Rega, G. Zheng, W. Liang, M. Hada, M. Ehara, K. Toyota, R. Fukuda, J. Hasegawa, M. Ishida, T. Nakajima, Y. Honda, O. Kitao, H. Nakai, T. Vreven, K. Throssell, J. A. Montgomery, Jr., J. E. Peralta, F. Ogliaro, M. Bearpark, J. J. Heyd, E. Brothers, K. N. Kudin, V. N. Staroverov, T. Keith, R. Kobayashi, J. Normand, K. Raghavachari, A. Rendell, J. C. Burant, S. S. Iyengar, J. Tomasi, M. Cossi, J. M. Millam, M. Klene, C. Adamo, R. Cammi, J. W. Ochterski, R. L. Martin, K. Morokuma, O. Farkas, J. B. Foresman, and D. J. Fox, Gaussian, Inc., Wallingford CT, **2016**.
8. Kossmann, S., and Neese, F., Comparison of two efficient approximate Hartree-Fock approaches *Chem. Phys. Lett.* **2009**, 481, 240-243.
9. Neese, F., The ORCA Program System *WIREs Comput. Mol. Sci.* **2012**, 2, 73-78.
10. Neese, F., and Valeev, E. F., Revisiting the Atomic Natural Orbital Approach for Basis Sets: Robust Systematic Basis Sets for Explicitly Correlated and Conventional Correlated ab initio Methods? *J. Chem. Theory Comput.* **2011**, 7, 33-43.
11. Gaussian 16, Revision A.03, M. J. Frisch, G. W. Trucks, H. B. Schlegel, G. E. Scuseria, M. A. Robb, J. R. Cheeseman, G. Scalmani, V. Barone, G. A. Petersson, H. Nakatsuji, X. Li, M. Caricato, A. V. Marenich, J. Bloino, B. G. Janesko, R. Gomperts, B. Mennucci, H. P. Hratchian, J. V. Ortiz, A. F. Izmaylov, J. L. Sonnenberg, D. Williams-Young, F. Ding, F. Lipparini, F. Egidi, J. Goings, B. Peng, A. Petrone, T. Henderson, D. Ranasinghe, V. G. Zakrzewski, J. Gao, N. Rega, G. Zheng, W. Liang, M. Hada, M. Ehara, K. Toyota, R. Fukuda, J. Hasegawa, M. Ishida, T. Nakajima, Y. Honda, O. Kitao, H. Nakai, T. Vreven, K. Throssell, J. A. Montgomery, Jr., J. E. Peralta, F. Ogliaro, M. J. Bearpark, J. J. Heyd, E. N. Brothers, K. N. Kudin, V. N. Staroverov, T. A. Keith, R. Kobayashi, J. Normand, K. Raghavachari, A. P. Rendell, J. C. Burant, S. S. Iyengar, J. Tomasi, M. Cossi, J. M. Millam, M. Klene, C. Adamo, R. Cammi, J. W. Ochterski, R. L. Martin, K. Morokuma, O. Farkas, J. B. Foresman, and D. J. Fox, Gaussian, Inc., Wallingford CT, 2016.
